# Plant heteropolysaccharides and their bioadhesion to animal glycocalyx visualized at the nanoscale

**DOI:** 10.1038/s42003-026-10872-y

**Published:** 2026-09-22

**Authors:** Irene Wacker, Willi Wagner, Ronald Curticean, Anna Schoch, Endre Majorovits, Jennifer M. Pan, Steven J. Mentzer, Mirko Bunzel, Rasmus R. Schröder

**Affiliations:** 1 BioQuant, Universität Heidelberg, Heidelberg, Germany; 2 3DMM2O, Cluster of Excellence (EXC-2082/1 – 390761711), Heidelberg, Germany; 3 Department of Diagnostic and Interventional Radiology, University Hospital of Heidelberg, Heidelberg, Germany; 4 Translational Lung Research Center Heidelberg (TLRC), German Center for Lung Research (DZL), University of Heidelberg, Heidelberg, Germany; 5 Department of Food Chemistry and Phytochemistry, Karlsruhe Institute of Technology (KIT), Karlsruhe, Germany; 6 Carl Zeiss Microscopy GmbH, Oberkochen, Germany; 7 Laboratory of Adaptive and Regenerative Biology, Brigham & Women’s Hospital, Harvard Medical School, Boston, MA USA; 8 Present Address: Diagnostic Radiology and Neuroradiology, Greifswald University Hospital, Greifswald, Germany

**Keywords:** 3-D reconstruction, Translational research, Polysaccharides

## Abstract

The plant-derived biopolymer pectin has been explored as sealant in lung surgery. Interpenetration of pectin’s heteropolysaccharide chains with sugar-chains on components of the mesothelial glycocalyx is thought to mediate adhesion. To visualize a potential adhesion complex, we first establish a method to preserve pectin ultrastructure by limiting hydration during sample preparation for electron microscopy. Depending on actual hydration states and pectic domains selected we find distinct morphologies: Citrus pectic polysaccharides, combining both, smooth homogalacturonan and hairy rhamnogalacturonan domains, form a loose network of thin filaments. Rhamnogalacturonan and xylogalacturonan domains from soy show a denser mesh, while pectic galactan from potato or pectic arabinan from sugar beet display more globular aspects. As proof of concept, we finally apply the filamentous lemon pectin to the surface of murine lung in a surgical setting. Ultrastructural visualization of the sealant-tissue assembly shows tight association between the carbohydrate fibers, possibly driven by polymer-typical chain interactions.

## Abstract (web summary)

Limited hydration during preparation of structurally different types of pectic polysaccharides allows nanoscale visualization of distinct morphologies and proof-of-principle analysis of bioadhesion of filamentous lemon pectin to murine lung surface

## Introduction

Usage of carbohydrate-based biopolymers such as cellulose^1^, chitosan^2^, or pectin^3,4^ in a biomedical context has a longstanding tradition. In particular, the chemical properties of pectins, a group of complex, branched polysaccharides, make them interesting, biocompatible candidates.

**Fig. 1:**
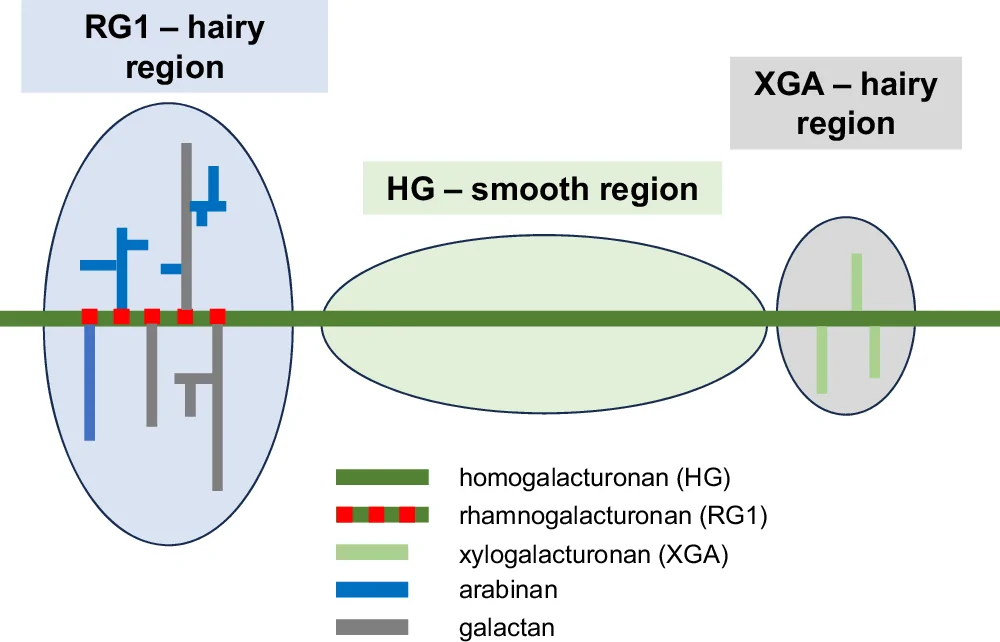
Schematic representation of a generalized pectin macromolecule. Shown are different possible domains (smooth vs hairy regions) and their polysaccharide composition.

Pectins are structurally diverse polymers, made up of several polymeric domains in varying quantities and being structurally different. The backbone consists of homogalacturonan (HG) and rhamnogalacturonan type I (RGI), with different types of polymeric side-chains attached. Branched arabinans and (arabin)galactans type I connect to RG1 regions in the backbone, xylose or short xylan chains to parts of the HG backbone (then denominated as xylogalacturonans (XGA)), while other parts of the HG backbone are devoid of substituents^5,6^. The traditional model of pectins differentiates “smooth”, that is unbranched, regions and “hairy”, that is branched, regions^5^. HG contributes to the smooth regions, whereas RGI (backbone and neutral side-chains) is usually the dominant structural unit of hairy regions (Fig. 1). In addition, the domains can largely differ from a structural perspective. For example, the galacturonic acid units of HG can be more or less esterified/acylated with methanol or acetic acid. In vivo, the structures of pectins depend on several factors, including taxonomy, plant tissue, and maturity, while in vitro, the extraction protocols largely affect the structures of the end products. Pectins are usually extracted under acidic conditions. Due to the different stability of glycosidic linkages in acid, specific domains are more severly degraded than others. Enzymes might also be used to more specifically degrade some pectin subunits^7^.

Applications of pectins in biomedicine range from gels for wound dressing to carriers for drug delivery^8^. Effects are usually mediated by bioadhesion or mucoadhesion^9^.

The usage of films fabricated from pectic polysaccharides (PPS) as potential sealants in surgery has been explored in the work of refs. ^10–12^. where interaction of a variety of PPS with the mesothelium of internal organs was investigated^10^. In a murine surgical model setting, it was found that a high methoxyl pectin film exhibited a strong bioadhesion, e.g., to lung pleura and could limit air leaks after pleural injury^11^, comparing favorably against commercially available surgical products.

When adhesion of the sealant to the pleura was inhibited by desiccation or removal of the glycocalyx from the pleura by enzyme treatment, sealant failure was observed^12^. This indicates a potential interaction mechanism by an initial swelling of the dry film when applied to the pleura, potentially allowing entanglement of pectins‘ carbohydrate chains with the sugar chains on the glycoproteins and glycolipids of the glycocalyx covering the mesothelial cells. The mechanism could therefore be based on a simple polymer-typical inter-chain entanglement, which—if verified—could be optimized further by future chemical modifications of the pectic material.

To prove the hypothesis that adhesion is caused by entanglement of pectin carbohydrate chains with the extracellular glycocalyx on visceral organs, the interaction of the different polysaccharides needs to be shown directly, preferably in an in vivo/in situ surgical setting.

Separate visualization of the individual components of the proposed interaction complex—pectins on one side and the glycocalyx on top of the mesothelial cell layer on the other side—has been achieved previously in ex situ, non-surgical experiments in different ways: The nanostructure of high methoxyl sugar acid pectin gels analyzed by AFM exposed curvilinear strands with junctions and branch points^13^. TEM analysis of pure and mixed high methoxyl and low methoxyl pectin gels with different chemical modifications and varying concentrations of the gelling agents Ca^2+^, sucrose and citrate, embedded in resin and sectioned before imaging, revealed highly porous networks of thin filaments^14,15^. Similar networks were shown in a study comparing TEM data from resin embedded pectin gels with SAXS measurements on native pectin gels, indicating that structural information above 20 nm is largely consistent between the two methods^16^. Super-resolution light microscopy of the glycocalyx of cell lines and primary human tumor cells enabled 10–20 nm precision in localization of two components of cell surface glycans after subjecting the cells to metabolic labeling and bioorthogonal chemistry^17^. Ultrastructural TEM imaging of the glycocalyx on mesothelium of visceral organs after high-pressure freezing and freeze substitution demonstrated a thick extracellular layer appearing as a filamentous network of high porosity^18^.

Despite of these promising results regarding visualization of pectin and glycocalyx as separate entities, their combination in a surgical setting remains challenging for several reasons: 1. The pectins we are using are not an already stabilized gel as used before^14,15^, but a glass-phase film without any additives that will experience a limited hydration in contact with the organ it is placed on. Addition of an aqueous chemical fixative would invariably lead to dissolution of the film. 2. Even freeze substitution of pectins alone after high-pressure freezing—which proved very successful for glycocalyx visualization^18^—did not work in previous experiments. Therefore, the alleged gold standard cryo-electron microscopy cannot be easily applied here. In addition, the surgical hybrid model (a plant-based pectin patch applied onto a mammalian visceral organ) cannot be vitrified without many additional sample preparation steps, such as the excision of the organ, sample punching for high-pressure freezing, cryo FIB-milling and lamella extraction for cryoTEM^19–21^.

Ultrastructural visualization of a pectin film in situ, i.e., adhering to a visceral surface, thus requires a sample preparation that fulfills both preservation of the pectin film itself in aqueous media and preservation of the tissue the film is interacting with. To achieve these combined goals, we sought to advance known protocols for biomaterial and tissue preparation, facilitating an optimal stabilization of the different material components. To limit the number of animals needed, we first established a more straightforward protocol for glass-phase pectin films alone using a chemical preparation method that allows to keep the films intact by limited hydration during processing for ultrastructural electron microscopy. The validity of this method was tested by analyzing different PPS of varying chemical structures. Finally, as proof of concept, we analyzed the adhesion of a pectin film displaying a loose network-like morphology to the lung surface at ultrastructural level. We revealed a tight interaction between pectin carbohydrate polymer chains and the glycocalyx of the mesothelial cell layer of visceral organs.

## Results

### Stabilization of pectin films in vitro by reducing free water

**Fig. 2:**
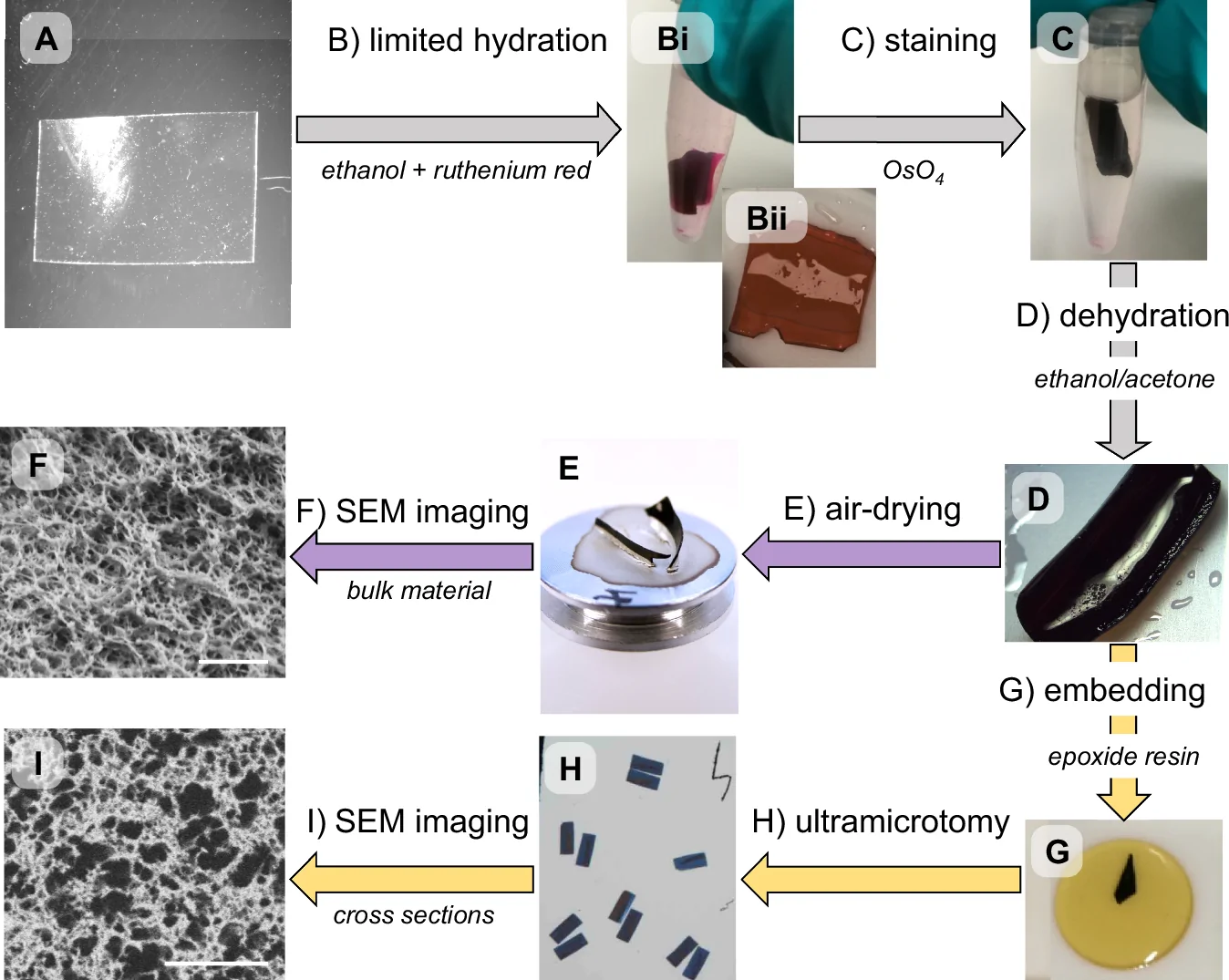
Stabilization of pectin films during preparation for high resolution SEM imaging. Glass phase pectin films (**A**) are partially hydrated (**B**) in 0.1 M pipes buffer containing 2.5% glutaraldehyde as fixative, 0.15% ruthenium red, and 15–40% ethanol (exact value depending on the type of pectin). To introduce contrast and conductivity for SEM imaging, OsO_4_ is used (**C**). After dehydration by an ascending ethanol series followed by acetone (**D**), samples are then either air-dried (**E**) or embedded in epoxy resin (**G**). Fracture faces of air dried samples mounted on aluminium stubs (**E**) are directly imaged in an SEM (**F**), while resin-embedded samples are cut into ultrathin sections and placed on pieces of silicon wafer (**H**) before SEM imaging (**I**). Scale bars 1 μm.

Since our glass-phase pectin films dissolve within a few minutes when brought into contact with aqueous solutions such as buffered fixatives, conventional embedding for ultrastructural analysis is impossible. To define conditions that would prevent dissolution of the pectin film, we systematically tested all solutions used during a typical chemical embedding procedure for their effect on pectin films (Table 1). We found that none of the components of the fixatives, applied individually or in different combinations together could prevent pectin dissolution. However, during the dehydration steps in organic solvents (15–100% ethanol, acetone), which are necessary before resin embedding, the films remained stable and even lost weight, presumably reducing their water content (above 70% ethanol). Therefore, we decided to add a low percentage of ethanol—the actual amount depending on the type of pectin being investigated (see below)—to the fixatives and all subsequent washing steps to keep pectin films intact. An exemplary workflow is illustrated in Fig. 2. Starting with a dry film (Fig. 2A), limited hydration is achieved by combining a fixative consisting of 2.5% glutaraldehyde and 0.15% ruthenium red in 0.1 M Pipes buffer with ethanol. The concentration of ethanol depends on the pectin type used and can vary from 15 to 40%. All aqueous solutions used in the following steps, including washing buffers, contained at least the initial concentration of ethanol (cf Suppl. Table 2). Ruthenium red is known to intercalate into certain binding sites present in pectins^22^, stabilizing it to some extent but primarily introducing a heavy metal as indicated by the red color in Fig. 2Bi and Bii. The Ru ions serve as seeds for another heavy metal, Os, which is added as aqueous OsO_4_ in the next step, leading to a blackening of the film (Fig. 2C). Together, both metals generate contrast for subsequent imaging in an electron microscope. Dehydration of the film in an ascending ethanol series followed by acetone produces a still partially hydrated, blackened film (Fig. 2D), which is then either air-dried for direct analysis or embedded in epoxide resin for sectioning. For direct analysis in a scanning electron microscope (SEM), the dried film is broken into small pieces to expose a fracture face and mounted on an aluminum stub (Fig. 2E). Alternatively, ultrathin sections are cut from the embedded sample (Fig. 2G), placed on a piece of silicon wafer (Fig. 2H), and also imaged in an SEM. Comparison of the two sample types (Fig. 2F, I) shows a fibrous network for this particular pectin (lemon) on both preparation routes.

**Table 1:** Behavior of lemon pectin films after 15 min incubation in solvents used during a typical preparation protocol for EM imaging

	Pipes	GA	RR	Ethanol	Status pectin	HF
Water					Dissolved	−/−
0.1 M Pipes	x				Dissolved	−/−
2.5% GA		x			Dissolved	−/−
25% ethanol				x	Swollen film	3.4
50% ethanol				x	Swollen film	1.74
70% ethanol				x	Film	1
100% ethanol				x	Shrunk film	0.4
Acetone					Shrunk film	0.9
Combine 2		x		25%	Swollen film	4.1
Combine 2	x	x			Dissolved	−/−
Combine 2	x			25%	Swollen film	6.2
Combine 3	x	x	x		Dissolved	-/-
Combine 3	x	x		25%	Swollen film	6.6
Combine 4	x	x	x	20%	Swollen film	8.6

*GA* stands for glutaraldehyde, *RR* for Ruthenium Red, *HF* stands for hydration factor, defined by weight (after incubation)/weight (initial), *n* = 1.

To determine the optimal hydration state for imaging a given type of pectin we varied the concentration of ethanol in the fixative (cf Suppl. Table 1) and all consecutive solutions (cf Suppl. Table 2) and determined water uptake by gravimetric analysis (Supplementary Fig. 1). Pieces of pectin film were cut to a size sufficiently large to minimize relative measuring errors and weighed at different steps in the course of the embedding protocol. To illustrate the reliability of this gravimetric analysis, Supplementary Fig. 2 shows the dehydration kinetics of lemon pectin after limited hydration in fixative.

**Fig. 3:**
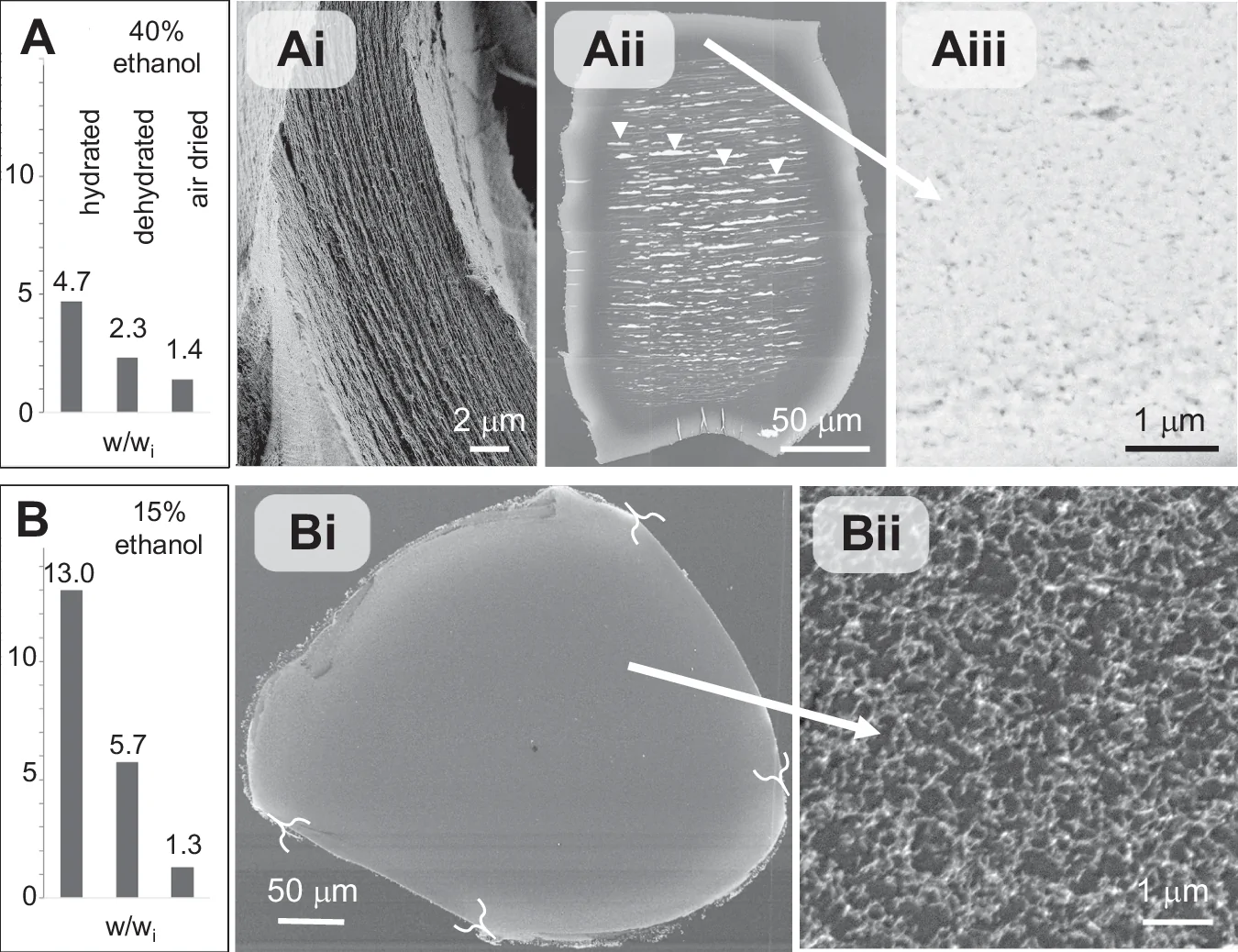
Determination of optimal hydration. Pectin films (lemon) were either hydrated in the presence of 40% (**A**) or 15% (**B**) ethanol in a fixative consisting of 2.5% glutaraldehyde, 0.15% ruthenium red in 0.1 M Pipes. The block diagrams show the degree of hydration (weight normalized to the original dry weight) at different steps during preparation for SEM imaging. Maximal hydration occurs after fixation (first bar), decreases during dehydration in organic solvents (middle bar) and reaches the original value after air drying (right bar). Films hydrated in the presence of high ethanol concentration show a layered appearance, both as fracture faces (**Ai**) and in cross sections (**Aii**), with very dense material packing visualized at high magnification (**Aiii**). Note the high number of cracks (arrowheads) in the cross section. At low ethanol concentration the material is more isotropically packed (**Bi**), presenting a filamentous network (**Bii**). Sample edges inside brackets are cut faces, outside brackets are original interfaces (to air/dish, respectively). For more details, see Supplementary Figs. S3 and S5 and movie_1.

During embedding procedures, in our canonical fixative (2.5% glutaraldehyde, 0.15% ruthenium red in 0.1 M Pipes buffer) plus added ethanol weights were only obtained after the initial fixation (“hydrated”), after dehydration in ethanol and acetone (“dehydrated”) and after air drying (Fig. 3A, B). In the presence of 40% ethanol, the maximal hydration achieved was about 5× the weight of the original film, while in 15% ethanol this value rose to 13×. Samples prepared with 40% ethanol in the fixative showed a very dense, layered structure of the fracture face view in the SEM (Fig. 3Ai). Embedded samples were rather difficult to cut into ultrathin sections, displaying many cracks and tears (arrowheads) in the cross-section (Fig. 3Aii) and showing a very densely packed material at higher magnification (Fig. 3Aiii). When reducing the ethanol content of the fixative to 15%, much smoother sections were produced (Fig. 3Bi), showing fibrous networks (Fig. 3Bii). The density of filaments was higher at the edge of the sample (Supplementary Fig. 3) with slightly different morphologies depending on the nature of the edge: Edges arising from cutting the small piece used for embedding are denoted as “cut” in the macroscopic image (Supplementary Fig. 3A) and by brackets in the cross-section (Fig. 3Bi). The cut pectin film surface shows a denser packing of filaments, creating a skin effect (Supplementary Fig. 3D). The original surface of the film, i.e., the interface to air or plastic dish produced edges in the cross section without a clear skin (Supplementary Fig. 3C). In the center of the sample filaments were more loosely packed, leading to comparable densities in the two different radial profiles extending about 100 micrometers from the two types of edges (Supplementary Fig. 3E, F and Suppl. movie_1). The fracture face of lemon pectin prepared with 20% ethanol was devoid of layering when comparing the overview (Supplementary Fig. 6A) with the fracture face of lemon pectin prepared with 40% ethanol (cf Fig. 3i). Higher magnifications showed a fibrous network for both, fracture face (Supplementary Fig. 6Ai) and cross section (Supplementary Fig. 6Aii).

### Chemical analysis and ultrastructural signatures of various types of pectin

Having established a reliable workflow for visualizing the ultrastructure of partially hydrated pectin films from lemon, we investigated films fabricated from structurally different PPS. Although the isolation and modification procedures of these commercially available pectins are not known, the structural analyses suggest that the lemon and orange pectins are simply extracted pectins (presumably under acidic conditions), whereas the PPS from potato, sugar beet, and probably soy have been additionally enzymatically modified in order to enrich (or eliminate) different pectic structural subunits.

**Fig. 4:**
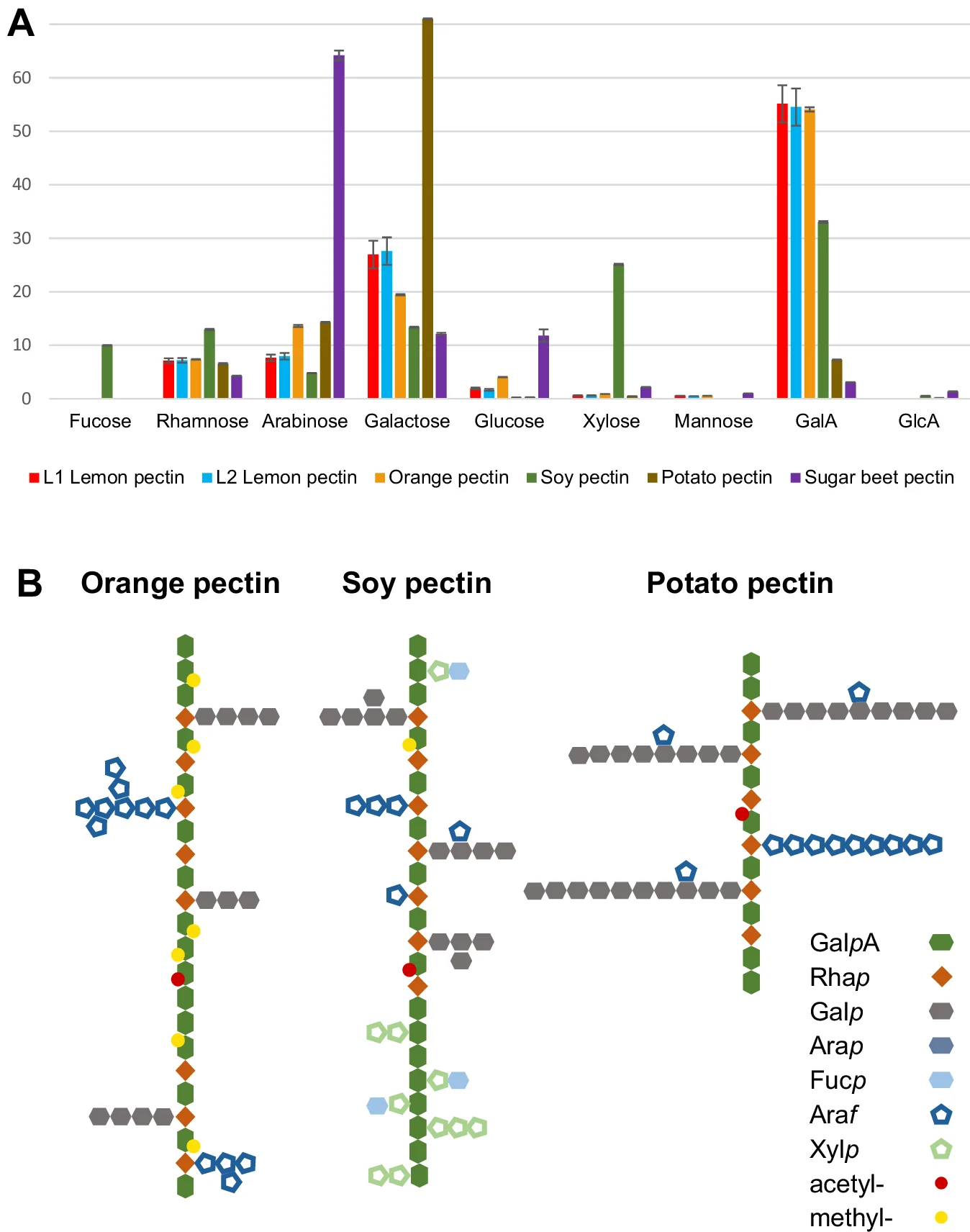
Monosacharide composition and structural representation of pectic polysaccharides. Monosaccharide composition of the applied pectic polysaccharides following methanolysis in mol%. The average of two technical replicates (*n* = 2) is plotted, with top and bottom of the error bars representing the two data points. GalA galacturonic acid, GlcA glucuronic acid (**A**). Source data underlying this graph can be obtained as Supplementary Data 1 and 2. Structural representation of three chemically different types of pectic polysaccharides stabilized and visualized (**B**). For more details of structural analysis, see also SI, pp 8–12.

Interpretation of the monosaccharide composition (Fig. 4, Supplementary Fig. 5, and Suppl. Table 3) and methylation analysis data (Suppl. Table 4) demonstrate that the lemon pectins (analyzed from two batches of films, L1 and L2) and the orange pectin show typical structures of commercial pectins extracted from the peels of citrus fruits. Both degree of methylation (lemon L1: 51.3%, lemon L2: 40.0%, orange: 40.4%) and degree of acetylation (DAc) were comparable (lemon L1: 0.9%, lemon L2: 0.7%, orange: 1.4% (Suppl. Table 5). Also, the molecular weight distribution is roughly comparable for the lemon pectins L1 and L2 as well as the orange pectin (Supplementary Fig. 4), although lemon pectin L2 polysaccharides appear to be slightly reduced in molecular weight. Finally, all three samples show an implied bimodal distribution of the PPS. Additional structural information is given in the SI (page 11).

Soy bean pectins are structurally different from other pectins with some interesting structural details. Studies of Schols´ and Voragen´s group revealed fucose as a component of XGA, whereas HG appears to be almost absent (in some fractions)^23^. Here, detection of substantial amounts of fucose as a monomer (Fig. 4A, Supplementary Fig. 5, and Suppl. Table 3) also suggests the presence of potentially fucosylated XGA. The monosaccharide compositional data also revealed a large proportion of xylose in the PPA from soy (Fig. 4A and Suppl. Table 3), likely from XGA. Arabinose and galactose have their origin from RGI carrying (short) arabinan and (arabino)galactan side chains. Size-exclusion chromatography (SEC) indicates a trimodal distribution of soy PPS, covering a wide molecular weight range (> 670–50 kDa when calibrated using dextran standards) (Supplementary Fig. 4). Additional structural information is given in the SI (page 11).

The potato “pectin” appears to be an enriched RGI fraction with galactans being clearly dominant (71% of the monosaccharides are galactose). The almost equal ratio of galacturonic acid and rhamnose (Fig. 4, Supplementary Fig. 5, and Suppl. Table 3) suggests a targeted degradation of HG during preparation of this sample. The RGI neutral side-chains of potato are usually dominated by galactans^24^. This is supported by methylation analysis data, demonstrating that 1,4-linked galactopyranose units are dominant (Suppl. Table 4). The 1,2,4-rhamnopyranose to 1,2-rhamnopyranose ratio of 2.2 suggests a dense RGI ramification. Just as in the soy pectin preparation used here, acetylation and methylation of the galacturonic acid units are very minor (Suppl. Table 4). The molecular weight distribution of the potato PPS suggests molecular weights being in between those of the larger citrus PPS and the smaller sugar beet PPS.

The sugar beet “pectin” appears to be an RGI fragment, too, as indicated by an almost equal rhamnose to galacturonic acid ratio. Thus, HG has been (enzymatically) removed. The RGI side-chains are highly enriched in arabinans. Native sugar beet pectins are also enriched in arabinans^25^, but the arabinan to galactan ratio of 5.3 appears to be more extreme here, suggesting a potential partial enzymatic galactan removal. Methylation anylsis data suggests a typical branched arabinan structure with linkages in position O3 being dominant^25^. The galacturonic acid units are highly acetylated (67%) (Suppl. Table 4). The PPS from sugar beet exhibit a bimodal molecular weight distribution, with the dominant fraction representing the smallest PPS used in this study (Supplementary Fig. 4).

Optimal hydration had to be defined for each pectin type, as a state in which the film could be carried through the fixation steps without dissolving, but at the same time giving an ultrastructure that was not just a compact block of material (as e.g., in Fig. 3Aiii). Films were incubated with all components of the primary fixative (cf Suppl. Table 1 for orange and lemon), single and combined and hydration factors were determined (Supplementary Fig. 1). From all films tested potato pectin required the highest concentration of ethanol (40%) to remain stable, whereas the other pectin types could be stabilized with lower ethanol concentrations of 15 or 20% (cf Suppl. Table 2).

**Fig. 5:**
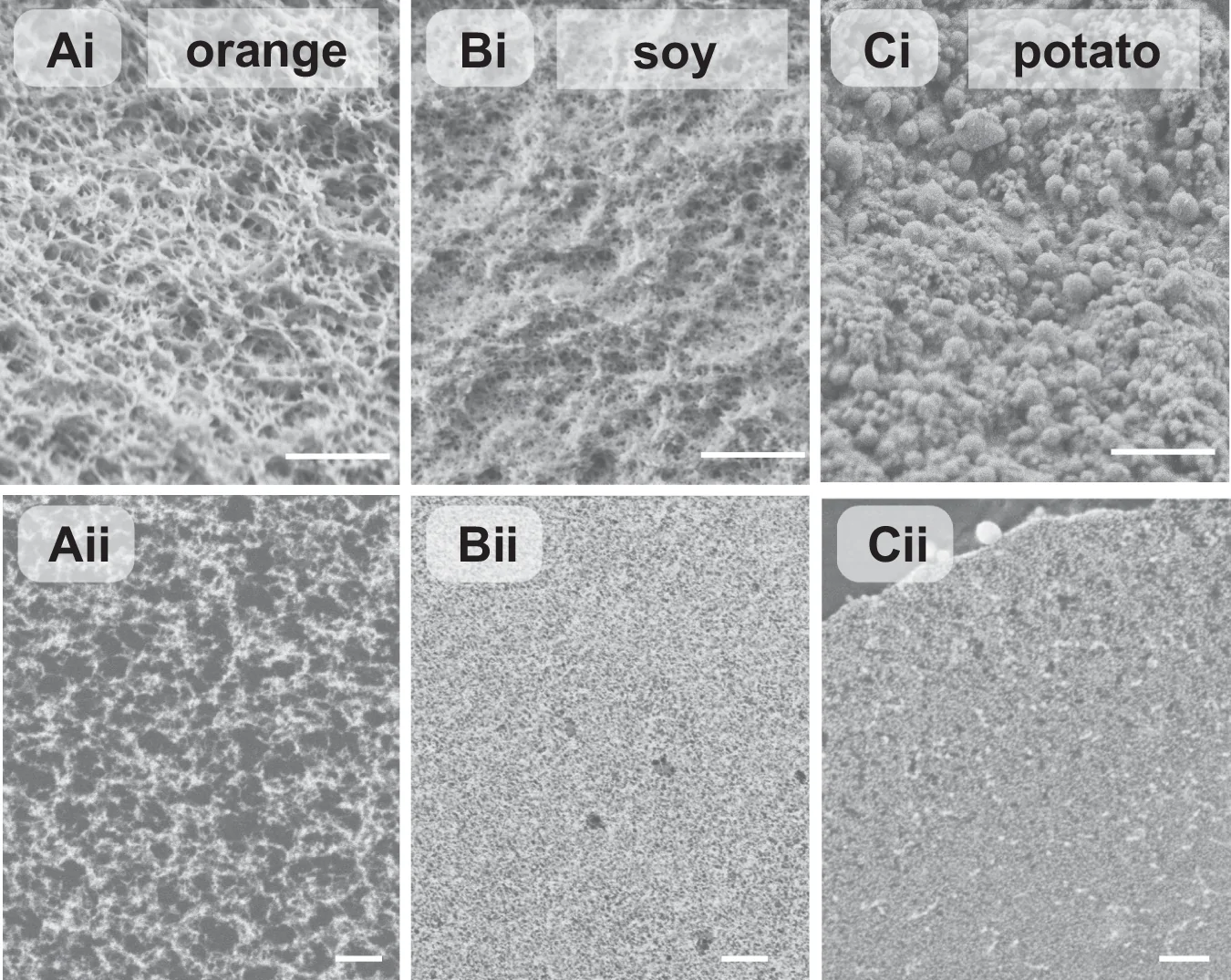
Pectic polysaccharides from different plants show different morphologies. Fracture faces from pectin films air-dried after staining with ruthenium red and OsO_4_ (i) or ultrathin cross sections from films embedded in epoxide resin after staining (ii) of pectic polysaccharides isolated from orange (**A**), soy (**B**), or potato (**C**). Samples were prepared with 15% ethanol in the fixative and 20% in subsequent solutions for (**A**, **B**) and with 40% ethanol in fixative and 50% in subsequent solutions for (**C**). Scale bars are 1 μm (i) or 400 nm (ii).

A comparison of the fracture faces in Fig. 5 (i) reflects differences in the structural composition: While the PPS from orange (Fig. 5Ai) and soy (Fig. 5Bi), both retaining smooth and hairy regions, had a filamentous appearance, the fracture face of potato PPS (Fig. 5Ci)—basically an RG1 (hairy) domain enriched for galactans—exposed a more granular, globular arrangement of coarser and more solid material.

These findings are consistent with images of the corresponding cross sections (Fig. 5ii), suggesting that the interconnected networks of orange and soy pectin were easier to stabilize than the agglomeration of globular particles in the case of potato pectin. Image analysis of the different cross sections by power spectral density and fractal dimension analysis showed a correlation between the type of pectic domains present in the respective sample and either network or densely packed morphology (Supplementary Fig. 7).

Analysis of more films fabricated from other PPS or mixtures of such with carboxymethylcellulose (CMC), another carbohydrate polymer, confirms these observations as shown in Supplementary Fig. 6 for lemon PPS alone (Supplementary Fig. 6A), and mixed with CMC (Supplementary Fig. 6B), sugar beet PPS (an RG1 domain enriched for arabinans) with CMC (Supplementary Fig. 6C), and CMC alone (Supplementary Fig. 6D) as control. In all cases, images of the fracture faces (left and central column) are consistent with images obtained from cross sections (right column, ii). PPS from lemon shows a similar fibrillar organization as that from orange, as expected based on chemical structural similarities, whereas the other materials appear as a mixture of globular and filamentous components. The globular components (arrowheads) are more obvious in the fracture face images, the filaments (arrows) in the cross sections.

### Pectin adhesion to lung surface in a surgical setting

As shown previously^10^, pectin films can bind to visceral organs and serve as sealant and potential drug delivery device. So far, the basis for the structural adhesion on the ultrastructural level was not known since visualization of the interface between pectin and organ surfaces had not been achieved yet. It has been hypothesized that a hydration-mediated microstructural entanglement between the carbohydrates of the glycocalyx—an extensive network of carbohydrate filaments on the lung mesothelial cells^18^—and the lemon pectin chains would mediate the adhesion of the pectin film.

**Fig. 6:**
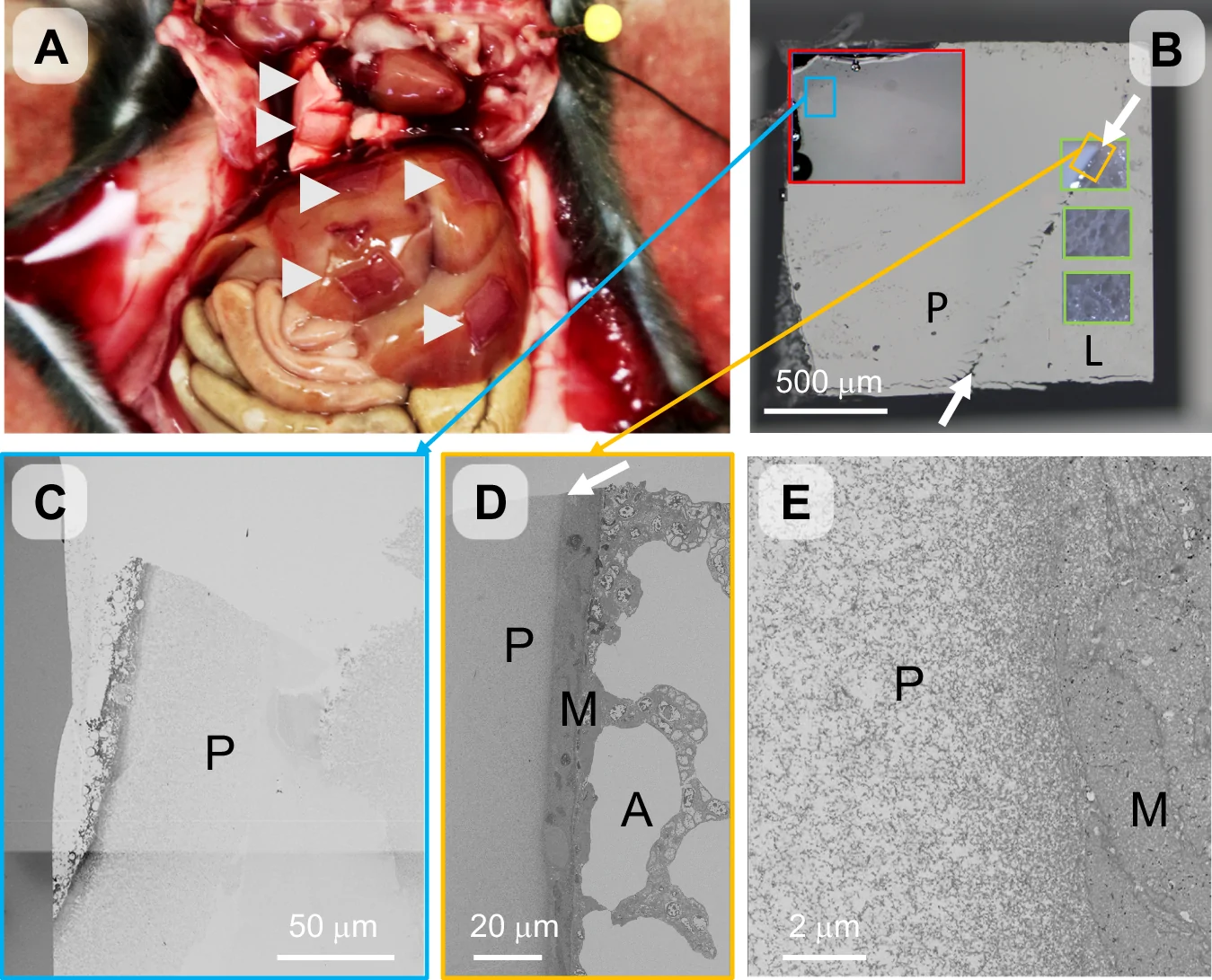
Pectin attachment to visceral organ surface. **A** Pectin patches (arrowheads) placed on murine lung and liver. **B** After embedding and trimming of the block, the interface (arrows) between pectin patch (P) and lung tissue (L) is clearly visible on the blockface (epi-illumination light microscopy) in bright field mode of entire blockface combined with darkfield mode for selected regions of lung tissue (green rectangles, overlays in software ZEN connect, see also Suppl. Fig. 11). **C**–**E** SEM images of sections showing edge of pectin patch (**C**), pectin–lung interface at intermediate (**D**) and high (**E**) magnification. P Pectin, M Mesothelial cell layer, A Pulmonary alveoli.

With our new workflow for pectin imaging, we set out to test this hypothesis. Small pieces (3 × 3 mm) of lemon pectin film (arrowheads in Fig. 6A) were placed on the surfaces of liver and lung of a mouse under a lethal dose of anesthetics. Pieces of tissue with adherent pectin patches were excised and immersion fixed in our optimized fixative with 15% ethanol and 0.15% Ruthenium Red added. To demonstrate that the inclusion of 15% ethanol in the fixative did not impair tissue preservation, we imaged control tissue taken from the same animal. Note that images of tissue and pectin film attached to tissue will be shown with inverted contrast to create an impression that is comparable to classical TEM ultrastructural histology and pathology imaging. As shown in Supplementary Fig. 8 mesothelial cells and a few cell layers immediately below them appear darker stained than deeper tissue layers, almost displaying a negative contrast. This is presumably caused by limited diffusion of the highly charged Ruthenium red into the topmost tissue layers, but not further as described before^22^. Pneumocytes, specifically type 2 alveolar epithelial cells, also exhibit dense cytoplasm with lamellar bodies (Supplementary Fig. 9B, C). Secreted surfactant is visualized adjacent to these cells (Supplementary Fig. 9D). Membrane-bound organelles such as Golgi stacks and endoplasmic reticulum (Supplementary Fig. 10A), coated vesicles and coated pits (Supplementary Fig. 10C) were visualized in a cell with less dense cytoplasm (Supplementary Fig. 10B). Suppl. Movie_2 shows the different regions of interest described in the context of the entire tissue piece.

After embedding and trimming of a tissue sample with attached pectin film, the surface of the block was imaged in a light microscope under epi-illumination conditions (Fig. 6B). The interface between the pectin patch and the tissue is clearly visible (arrows, see also Supplementary Fig. 11C, D). Higher resolution images from the regions marked in green show well-preserved tissue features of the lung surface with the pectin patch (P), the lungs glycocalyx, the mesothelial cell layer (M), the basement membrane and subpleural alveoli (A) from left to right (Supplementary Fig. 11D–F), but no discernible details in the area of the pectin material (red rectangle). SEM imaging of the corresponding regions on ultrathin sections (Fig. 6C–E) reveals one corner of the pectin patch only (Supplementary Fig. 11C), and pectin attached to the tissue surface (Supplementary Fig. 11D). The tightness of the interaction of pectin with the tissue is illustrated in Supplementary Fig. 11E at high magnification, where also the network character of pectin becomes obvious. Due to this very tight association between pectin and tissue observed in these samples, it was not possible to distinguish glycocalyx from pectin.

**Fig. 7:**
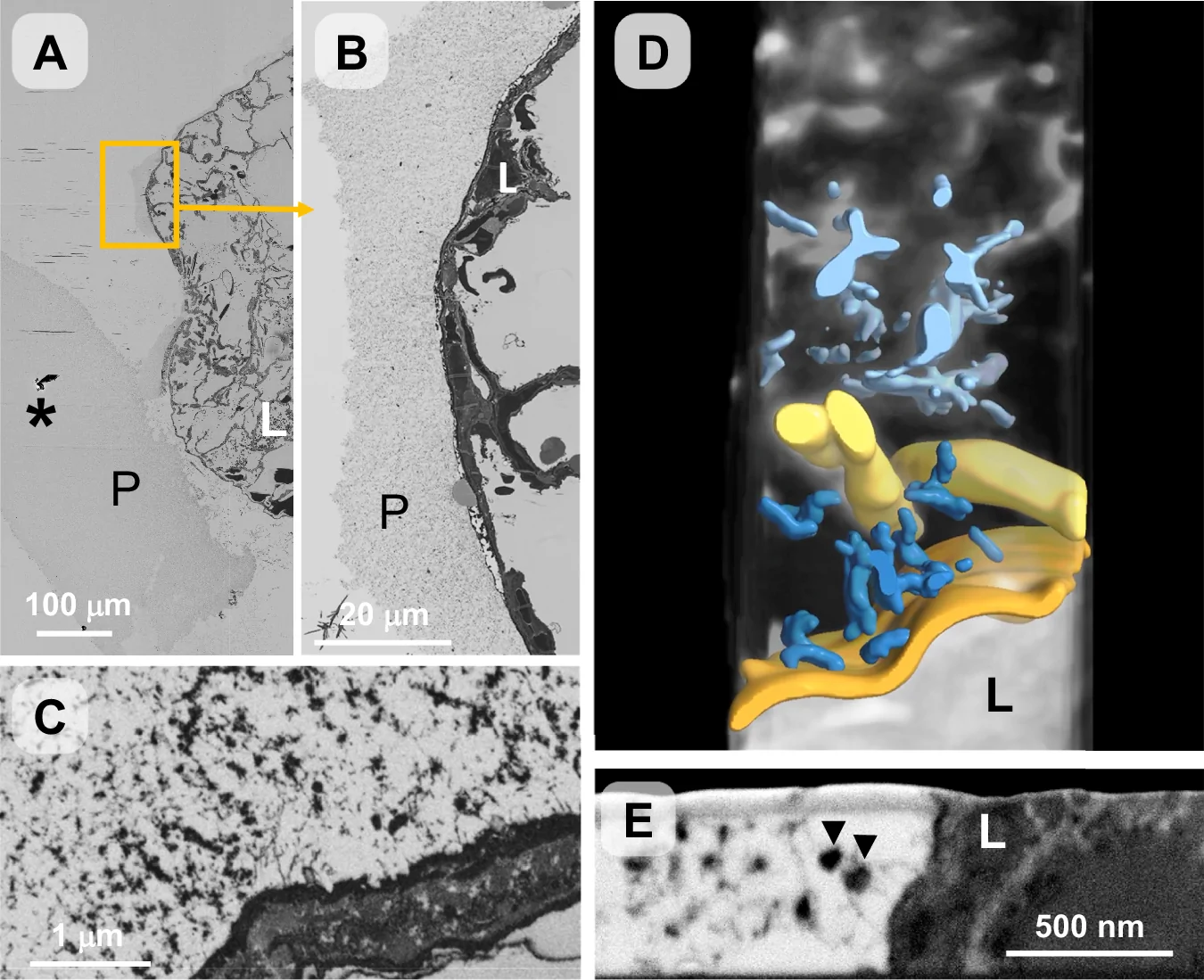
Interaction of pectin with glycocalyx. Cross section of pectin patches (P) at lung (L) surface. **A** overview, star indicates delaminated pectin patch, **B** detail with tightly attached pectin patch, **C** high resolution image showing filamentous and globular material outside the lung tissue. **D** 3D rendering of a FIBSEM nanotomography stack (recorded with 5 nm voxel size, see also Suppl. Movie_3) from a 500 nm thick section. Lung surface represented in orange, microvilli in yellow, fibrils close to surface in blue, peripheral fibrils in light blue. See also Suppl. Movies _4a–c for better impression of 3D data. **E** Sum over 9 slices (45 nm in z-direction) showing microvilli cross sections (arrowheads) and thin filaments in contact with the lung surface.

As a next step, we tried to achieve a less tight interaction by perfusion fixing the sacrificed animal for about 10 min to give the pectin film more time to hydrate before being excised with the underlying piece of tissue. Utilizing such a protocol, we could identify regions on the lung surface where parts of the pectin patch had delaminated from the tissue surface (Fig. 7A, star), but smaller pieces were still attached (Fig. 7A orange box, B). In such regions at high magnification, the glycocalyx could clearly be visualized with spherical microvilli cross sections and fine strands between them and also emanating from the cell surface (Fig. 7C). To distinguish microvilli cross sections from “glycocalyx globules”^18^, presumably crossing points of filaments in a projection view, we did a 3D analysis by FIBSEM nanotomography (data in Suppl. Movie_3). 3D reconstruction of an image stack recorded with a voxel size of 5 nm from such a region targeted in a 500 nm thick section by darkfield light microscopy (Supplementary Fig. 12) shows the microvilli (yellow structures in Fig. 7D) and between them and the cell surface a filamentous material (dark blue in Fig. 7D). For annotation of this dark blue material we only selected fine strands originating from the cell surface or from the surface of the microvilli (as e.g., shown in Fig. 7E).

Filamentous structures outside the region defined by the microvilli are annotated in light blue (see also Suppl. Movies_4a–c for different angles of the 3D rendering and segmentation). It is obvious that there are no morphological differences between the two annotated networks, but it is also clear that at some distance from the cell surface, the pectin patch must start. However, without sugar domain-specific labeling, it is not possible to identify the transition between glycocalyx and pectin patch.

## Discussion

In order to visualize on the nanoscale a possible entanglement of PPS of varying origin and different chemical structural features with polysaccharides of the glycocalyx on lung mesothelial cells, it was paramount to combine the requirements for good ultrastructural tissue preservation with stabilization of pectin through the entire course of the electron microscopy sample preparation. This was achieved by limited hydration, which helped to stabilize pectin during the chemical fixation and has not been employed before.

In our surgical setting, we used dry pectin films, which had been prepared without any additives and were initially only hydrated by water uptake from the organ surface. In contrast, wet pectin gels were used in earlier attempts to visualize pectin ultrastructure, and they were stabilized for EM imaging by lowering the pH and adding high concentrations (30–50%) of sucrose, 100 mM NaCl, or complexing ions such as Ca^2+^ in different combinations to the fixatives, depending on the composition of the pectin gels^14,15^. Usage of such additives was not advisable in our combined system of animal tissue and pectin patch for the following reasons: Low concentrations of Ca ions have been used successfully in fixatives for EM before^22^, but pH values as low as 3.0 are not desirable for animal tissue fixation since average pH values in mammalian cytoplasm are usually between 7.0 and 7.4^22^. Furthermore, the addition of sucrose would increase osmolarity of the fixative to such an extent that the tissue would massively loose water, leading to shrinkage artifacts. Also, the chemical structures of some of the PPS used—such as those of PPS from sugar beet and potaoes, which are essentially lacking in HG and therefore lack the regions typically used to form junction zones, do not allow for gel formation under the conditions mentioned before. Seeking another way to limit water uptake into the films, we found that the presence of a certain amount of ethanol in the aqueous fixative is limiting hydration of the film, which then allowed further processing for ultrastructural electron microscopy. This finding aligns with the fact that polysaccharides are precipitated from aqueous ethanol solutions, with the ethanol concentration required being dependent on the molecular weight and the chemical structures of the polysaccharides. Gravimetric analysis at different time points during embedding revealed that an initial hydration factor (after fixation) between 10 and 15 produced pectin morphologies with well separated fibrillar or globular arrangements—depending on the PPS used. Higher hydration factors led to dissolution of the films, while lower factors resulted in rather densely packed material where no fine structural features were discernible. Even in a hydration regime which just prevented dissolution of the film (15% ethanol for the lemon and orange based pectins) a frequent observation was that of a hydration gradient with a looser packing of filaments in the centre of the film (cf Supplementary Fig. 3). This skin effect has been observed before on pectin gels embedded for TEM^16^ and was attributed to the presence of Ruthenium Red which we also used as a staining agent.

Analysis of lung tissue subjected to this modified embedding procedure—optimized for retention of pectin films—demonstrated well preserved structural features (Supplementary Figs. 8–10) with a high degree of ultrastructural information.

As a next step, we investigated the interaction of pectin with the glycocalyx on lung mesothelium. Although pectin had been used as surgical sealant before—either in combination with CMC^10–12^ or with other polymers^9^—the structural details of its adherence mechanism to the mesothelium have only been speculative. As for other wound closure systems or for mucoadhesion in general, entanglement models have been proposed^9^, assuming an interpenetration of the sealant polymer chains with the carbohydrate chains of the glycocalyx in our case. However, a successful visualization of a postulated interaction complex for mucoadhesion at the ultrastructural level had not been achieved, even using cryofixation and freeze substitution of a model system consisting of rat intestinal mucus and a mucoadhesive polymer^26^. Here we visualize for the first time the postulated interaction complex in both LM and EM (cf Fig. 6). We show that a pectin film can firmly adhere to the mesothelial surface and survive the lengthy embedding procedure for EM analysis. In darkfield epi-illumination LM, the partially hydrated film appears as milky substance tightly attached to the lung surface. Ultrastructurally, this film shows the typical fibrillar network also observed for the PPS from lemon. In samples with a short hydration time, the attachment is so close that it is not possible to discern individual structural components, neither in the presumed animal glycocalyx nor in the plant-derived pectin patch. Extended hydration times produced samples with pectin patches adhering less strongly. Here, individual filaments originating from the surface of mesothelial cells and from microvilli could be detected (cf Fig. 7C, E) intermingling with a fibrillar network that extends several micrometers away from the tissue surface.

Although we cannot know the origin of a specific fibril within the putative interaction zone—plant or animal—the morphological convergence is remarkable, especially for the PPS from lemon that we chose. Since it is known that after chemical fixation, the width of the observable glycocalyx is about 10 times smaller than the 13 μm observed after cryopreparation^18^, we cannot give a reliable measure for the thickness of the transition zone. Analysis by 3D stimulated Raman spectral imaging of the transition zone between pectin and porcine intestinal serosa in fully hydrated, non-fixed state found a thickness of about 15 µm^27^ but could not provide ultrastructural detail.

In view of the structural similarity of the glycocalyx with the fibrous structure of the citrus-based PPS shown here, the question arises whether pectin as a natural sealant can be considered more than just biocompatible: The carbohydrate-based material might rather be regarded as a bifunctional sealant, which could engage in perfect polymer-chain interpenetration of its branched carbohydrates with the glycocalyx surface of the target organ, while at the same time presenting a glycocalyx-like surface to other internal organs in the body. Based on emerging findings on the role of the glycocalyx in vascular endothelial and immune functions^28–30^ it is tempting to speculate whether the structural homology of the plant-derived material pectin to glycans produced by the body itself will extend to a functional potential regarding signaling or mechanical properties. This will certainly need more research in the future.

To conclude: Using the concept of limited hydration by adding a low amount of ethanol during fixation of high-methoxy pectin films, we found a way to stabilize hydrated films and visualize their ultrastructure. SEM images of pectin films fabricated from several plant sources revealed different ultrastructural signatures for the various films with consistent morphology for each pectin type when comparing fracture faces with cross sections. Putative entanglement of lemon pectin’s carbohydrate chains with the glycocalyx on lung mesothelium was analyzed by single section high resolution imaging and FIBSEM nanotomography of pectin patches adhering to the surface of mouse lung. A dense material strongly attached to the mesothelial cells was visualized by darkfield light microscopy of embedded samples. At the nanoscale, a meshwork of filaments arising from cell surfaces and microvilli was found, extending up to several hundred micrometers above organ surface, much further than the known extent of the native glycocalyx.

These results support the initial hypothesis that certain types of pectin formulations—here the PPS from lemon that still show the typical smooth/hairy region structure—consist of fibrous material facilitating an interaction with complementary polymer chains in the glycocalyx of mammalian visceral organs. Such an interaction is in fact expected for homologous glycan-glycan polymeric structures and would give a direct mechanical explanation for the bioadhesion of certain pectin patches in a surgical setting. This finding might also open new routes for optimization of adhesion and subsequent resorption by chemical modifications focused on improving interaction and kinetics of swelling and disintegration after wound healing. It will be interesting to test whether our approach of limited hydration is also applicable to rationally designed composite bio-derived hydrogels with advanced stability and toughness^31–33^.

## Methods

### Materials and reagents

The pectins used in this study were obtained from two commercial sources: Lemon and orange pectin from Cargill, Minneapolis, MN, USA, potato pectin (Pectic Galactan), soy pectin (Rhamnogalacturonan), and sugar beet pectin (Arabinan) from Megazyme, Bray, Ireland. Pectin powder was stored in low humidity at 25 °C. CMC was obtained from Sigma-Aldrich (St. Louis, MO, USA). Ruthenium Red (“for microscopy”) and tannic acid (ACS reagent) were purchased from Sigma-Aldrich; PIPES (buffer grade) from AppliCHem PanReac, paraformaldehyde (16% EM grade), glutaraldehyde (25%, EM grade), OsO_4_ (crystals for making 5% stock in ethanol), Uranyl acetate, and lead citrate (3% ready to use solution) from Science Services. All components for the Epon mix (42.4 g glycid ether 100, 29.6 g dodecenylsuccinic acid anhydride, 18.4 g methyl-5-norbornene-2,3-dicarboxylic anhydride and 2.4 g benzyldimethylamine as initiator) were bought from Serva. All other materials were reagent grade.

### Fabrication of pectin films

Preparation of the pectin films has been described in detail elsewhere^34^. Briefly, the pectin powder was dissolved to 3% (w/w) at 25 °C by a step-wise addition of deionized water to first induce swelling and softening of the particles before fluidization and full dissolution. To complete dissolution a high-shear 10,000 rpm rotor-stator mixer (L5M-A; Silverson, East Longmeadow, MA) was used, monitoring the viscosity via the instrument’s data logger software. After 2 h of continuous stirring at room temperature, the dissolved pectin was poured into custom molds and cured to glass-phase films, also at room temperature.

### Analysis of pectin films

Pectin films were solubilized/suspended in water and freeze-dried. The polysaccharides of the different origins were analyzed with respect to their monosaccharide composition, degree of galacturonic acid methylation and acetylation, gylcosidic linkages, and molecular weight distribution. Monosaccharide composition was analyzed by HPAEC-PAD following methanolysis and trifluoroacetic acid (TFA) hydrolysis, following the principle described in ref. ^35^. In brief, methanolysis was performed in 1.25 M methanolic HCl for 16 h at 80 °C. Following evaporation, 2 M TFA was added, and the solution was heated for 1 h at 121 °C. Following co-evaporation with ethanol, D-mannitol was added as quality control standard. HPAEC-PAD was performed on a CarboPac PA-20 column as described in detail in ref. ^36^. Calibration was performed externally, and samples were analyzed in duplicate.

Galacturonic acid contents to calculate both the degree of methyl esterification and the DAc were determined using a recently published LC-MS-based stable isotope dilution approach^37^. In brief, the solubilized polysaccharides and added ^13^C_6_-galacturonic acid (internal standard) were treated with a solution of 12.5 mM sodium tetraborate in concentrated sulfuric acid in order to degrade both the polysaccharides and the internal standard. Following careful, partial neutralization of the acid on ice, the resulting degradation product, 5-formyl-2-furancarboxylic acid, both unlabeled (5-FFA) and fully labeled (^13^C_6_-FFA), was extracted into ethyl acetate. Ethyl acetate was evaporated, the residue was redissolved in 10% aqueous acetonitrile, diluted and analyzed by using LC-orbitrap-MS following separation on an UHPLC C18-column. Esterified acetic acid and methanol were analyzed according to a method published by ref. ^38^. The sample material was treated for 2 h with 2 M NaOH in D_2_O containing the sodium salt of 3-(trimethylsilyl)propionic-2,2,3,3-*d₄* as internal standard (TMSP), centrifuged, and the supernatant was membrane-filtered and analyzed by quantitative ^1^H-NMR using a 30° pulse (zg 30), a relaxation delay of 35 s, and 32 scans. The following signals were used for quantification: referencing against TMSP (δ 0.00 ppm); acetate (δ 1.92 ppm); methanol (3.36 ppm). Contents were calculated by comparing the integrals of the acetate and methanol signals with the signal of TMSP.

The distribution of glycosidic linkages in which the individual neutral sugars are in involved in the polysaccharides was analyzed by methylation analysis according to ref. ^39^. Dried samples were dissolved in DMSO and methylated using dry, pulverized NaOH and methyl iodide. Following addition of sodium thiosulfate solution, the permethylated polysaccharides were extracted into dichloromethane. Following evaporation of the solvent, the samples were dried. In order to reduce undermethylation, methylation was repeated, followed by hydrolysis and derivatization: 2 M TFA was added to the dried residues, and the suspensions were treated at 121 °C. Following evaporation, the liberated partially methylated monosaccharides were reduced using sodium borodeuteride in 2 M ammonia solution. The reaction was stopped by adding glacial acetic acid. Acetylation was performed by adding acetic acid anhydride, catalyzed by 1-methylimidazole.

On ice, the solution was carefully diluted with water, and the partially methylated alditol acetates were extracted into dichloromethane. The organic phase was separated and residual water was frozen-out. Analysis of the partially methylated analysis was performed by GC-MS and GC-FID. While GC-MS was used for identification purposes, GC-FID was used for semiquantitative determination using reponse factors based on the “effective carbon response” described by ref. ^40^.

The molecular weight distribution of the soluble PPS was determined by HPSEC with refractive index detection, using two SEC columns in row (TSKgel G6000PWxl (300 × 7.8 mm, 13 μm particle size) and TSKgel G4000PWxl (300 × 7.8 mm, 10 μm particle size))^24^. The separation was performed at 50 °C, using 50 mM sodium nitrate as eluent. Dextrans (5, 12, 50, 150, and 670 kDa) were used for calibration.

### Animals

We have complied with all relevant ethical regulations for animal use. All experiments were conducted in agreement with national and international guidelines. Note: the mice were only used as a source of tissue to test adhesion of a pectin film to a visceral surface and not as experimental animal system. Four twelve-weeks-old male and female mice, wild type C57BL/6 were anesthetized prior to euthanasia. The care of the animals was consistent with guidelines of the German Animal Welfare Law. Studies were approved by the animal care and use committees of University Hospital Heidelberg under the registration number T-30/23. In two independent experiments, two animals/experiment were euthanized with intraperitoneal injection of 100 mg/kg ketamine hydrochloride with 10 mg/kg xylazine hydrochloride. Mice were placed in supine position, the thoracic and abdominal cavity were opened and the right ventricle cannulated with a 20 G catheter, an incision was made to the inferior caval vene to later allow drainage of blood and fixative. The trachea was cannulated using a blunt 20-G cannulas the lung air inflated to 25 cm H_2_O pressure. 3 × 3 mm pectin patches were carefully applied to the wet surfaces of visceral organs. In the first experiment, small organ samples with and without pectin patches were then directly harvested and immersed in primary fixative (1.25% glutaraldehyde, 2% paraformaldehyde, 0.15% Ruthenium red, and 15% ethanol in 0.1 M Pipes buffer). In the second experiment, the animals were vascular perfusion fixed via the right ventricle using the primary fixative before small organ samples containing pectin patches were harvested after 10 min of perfusion fixation and submerged in the same fixative.

### Gravimetric analysis

Pectin films (40–100 μm thick) were cut in 4 × 10 mm pieces and weighed. After treatment with the fixatives (see below), i.e., after about 2 h in aqueous solution, the hydrated films were weighed again. Since observation in the SEM samples requires dry samples, the hydrated films were slowly dehydrated through a graded ethanol series and acetone, weighed again and then air dried. For some experiments, additional weight measurements were taken after each dehydration step. The weight values were normalized to the initial weight and the obtained “hydration factor” plotted in Excel.

### Sample preparation for microscopy

Pectin films were cut in 2 × 5 mm pieces, sometimes also in a triangular shape, and incubated for 30 min in the primary fixative, consisting of 2.5% glutaraldehyde, 0.15% Ruthenium red, and 15–40% (depending on the type of pectin) ethanol in 0.1 M Pipes buffer pH 6.8. After washing for 30 min with 20% ethanol in 0.1 M Pipes (2–3 solution changes), the secondary fixation was for 1 h in 1% OsO_4_, 0.15% Ruthenium red, 20–50% ethanol in 0.1 M Pipes. For potato pectin, a much higher concentration of ethanol was required to keep the film stable throughout the entire embedding procedure. Suppl. Table 2 gives the exact ethanol concentrations for all pectins during all incubation steps in aqueous medium. After washing as above the samples were dehydrated in a graded ethanol series (50, 70, 90, 100%) followed by ethanol/acetone (1:1) and dried acetone twice, each step for 10 min. Then samples were either air-dried or infiltrated with 30% Epon in dried acetone for 1 h, 70% Epon in dried acetone for 1 h, and 100% Epon overnight, followed by fresh 100% Epon for 4 h. For polymerization (2 d at 65 °C), the samples were transferred to silicon molds filled with 100% Epon.

For visualization of pectin patches on mouse lung, the protocol was slightly adjusted: The primary fixative was 1.25% glutaraldehyde, 2% paraformaldehyde, 0.15% Ruthenium red, and 15% ethanol in 0.1 M Pipes buffer. After the secondary fixative (prepared as above), an additional step was introduced to increase tissue contrast: Overnight incubation with 2% Uranyl acetate in 25% ethanol. Dehydration steps were increased to 15 min/step and the initial infiltration steps to 2 h/step.

Air-dried pieces of pectin films were cut into smaller pieces and mounted with silver paint (Acheson, Plano, Wetzlar, Germany) onto aluminum stubs.

Resin-embedded samples were processed further by ultramicrotomy: Sections (70–100 nm for 2D imaging, 500 nm for nanotomography) were produced using a Powertome PC ultramicrotome (RMC Boeckeler, Tucson, USA). For thin sections an Ultra 35° diamond knife was used, for thick sections a HistoJumbo 45° (both Diatome, Biel, Switzerland). Sections were collected on pieces of Si wafer before post-staining with 3% aqueous uranyl acetate followed by 3% aqueous lead citrate.

### Light and electron microscopy

An upright microscope (AxioImager 2, Carl Zeiss Microscopy, Oberkochen, Germany) with epi-illumination and long-distance objectives was used in brightfield or darkfield mode for mapping of embedded sample through the trimmed blockface or of thick sections on pieces of silicon wafer. The software package ZENconnect (Carl Zeiss Microscopy, Oberkochen, Germany) allowed a seamless transfer of these datasets to the instruments used for ultrastructural analysis, see below.

SEM hierarchical imaging at 1.5 keV landing energy with a 20 μm aperture using SE and ESB detectors was performed with the Atlas 5 AT software package on a Ultra55 SEM (Carl Zeiss Microscopy).

FIBSEM nanotomography was carried out on a Crossbeam 540 (Carl Zeiss Microscopy) at 2 keV landing energy, 1.0 nA beam current with an isotropic voxel size of 5 nm in x, y, and z. FIB milling was performed at 30 kV with an ion beam current of 700 pA.

The ZENconnect LM dataset was imported into the Atlas 3D software package for targeting of relevant pre-selected regions. InLens and ESB (1000 V grid) detectors were used simultaneously for image recording.

### Image analysis

For registration of nanotomography stacks, the TrakEM module^41^ of the open-source software Fiji was used. Registered substacks were annotated manually in Fiji^42^ and visualized in Chimera^43,44^. All other operations on images (merging of slices by the command z-projection (from stack operations) using “sum slices”), filtering, etc., were also done in Fiji.

### Statistics and reproducibility

Chemical analyses were done as technical replicates of *n* = 2 for each type of PPS studied. For lemon-derived PPS duplicates from two different material batches (biological replicates) were analyzed. Values given are average ± range/2).

Gravimetric analyses were usually *n* = 1, with lemon PPS always as reference when hydration levels were optimized for other PPS. In the dehydration kinetics experiment (Supplementary Fig. 2), technical replicates (*n* = 3) were analyzed to check how reliable small pieces of film could be weighed.

Image analysis of the network characteristics of different PPS (Supplementary Fig. 7) was done on *n* = 3 different regions of interest selected from the same cross section of the respective resin-embedded PPS. Plotted are mean ± standard deviation^44^.

## Supplementary information

Supplementary Information

Description of Additional Supplementary Materials

Supplementary Movie 1

Supplementary Movie 2

Supplementary Movie 3

Supplementary Movie 4a

Supplementary Movie 4b

Supplementary Movie 4c

Supplementary Data 1

Supplementary Data 2

Transparent Peer Review file

## Data Availability

All data are available via the data repository heiDATA under the following https://doi.org/10.11588/DATA/5LW3JS Source data underlying graphs for the Fig. 4A can be obtained from Supplementary Data 1 and 2.
