# Supplementary material for: Plant heteropolysaccharides and their bioadhesion to animal glycocalyx visualized at the nanoscale
Source: Commun Biol. 2026 Sep 22;9:1238. doi: 10.1038/s42003-026-10872-y (PMC13597490; doi:10.1038/s42003-026-10872-y)

26.02.26	Calibration	Peak area calibration										
µM	pmol	Fucose	Rhamnose	Arabinose	Galactose	Glucose	Xylose	Mannose	Fructose	Apiose	GalA	GlcA
1 µM	25	0,501	0,236	0,389	0,679	0,905	0,532	0,351			0,342	0,444
6 µM	150	2,848	1,403	2,254	3,638	4,367	3,010	2,252			1,978	2,647
12 µM	300	5,460	2,716	4,396	6,906	8,144	5,839	4,486			3,954	5,295
18 µM	450	7,481	3,809	6,205	9,505	11,074	8,055	6,367			5,751	7,549
25 µM	625	**9,840**	**5,085**	**8,333**	**12,438**	**14,568**	**10,773**	**8,763**			**8,134**	**10,537**
50 µM	1250	16,287	9,212	15,313	20,526	24,185	18,558	16,077			16,384	20,243
75 µM	1875	20,890	12,529	20,423	26,613	30,980	24,240	22,778			24,753	29,871
100 µM	2500	24,584	15,299	24,610	31,385	36,650	28,885	28,695			33,371	39,533
125 µM	3125	27,230	17,496	27,594	34,636	39,871	31,463	31,641			40,858	47,727

GalA, galacturonic acid; GlcA, glucuronic acid;

Calibration 1-25µM

	Fucose			Rhamnose			Arabinose			Galactose			Glucose			Xylose		
	**B1**	**B2**	**c**	**B1**	**B2**	**c**	**B1**	**B2**	**c**	**B1**	**B2**	**c**	**B1**	**B2**	**c**	**B1**	**B2**	**c**
**Value**	-6,647E-06	1,980E-02	2,7525E-02	-2,537E-06	9,695E-03	2,9515E-03	-3,539E-06	1,549E-02	1,2904E-02	-8,429E-06	2,499E-02	7,7490E-02	-9,513E-06	2,878E-02	2,3371E-01	-5,716E-06	2,068E-02	3,8515E-02
**Standard error**	1,245E-06	8,388E-04	1,1180E-01	4,729E-07	3,186E-04	4,2464E-02	7,168E-07	4,828E-04	6,4356E-02	1,243E-06	8,371E-04	1,1158E-01	2,025E-06	1,364E-03	1,8183E-01	1,504E-06	1,013E-03	1,3502E-01
**Coefficient of determination**	**0,99960**	1,048E-01	#N/A	**0,99978**	3,982E-02	#N/A	**0,99982**	6,034E-02	#N/A	**0,99975**	1,046E-01	#N/A	**0,99950**	1,705E-01	#N/A	**0,99951**	1,266E-01	#N/A

	Mannose			Fructose			Apiose			GalA			GlcA		
	**B1**	**B2**	**c**	**B1**	**B2**	**c**	**B1**	**B2**	**c**	**B1**	**B2**	**c**	**B1**	**B2**	**c**
**Value**	-2,311E-06	1,544E-02	-1,6266E-02	#VALUE!	#VALUE!	#VALUE!	#VALUE!	#VALUE!	#VALUE!	1,836E-07	1,279E-02	4,1402E-02	-1,447E-06	1,765E-02	2,5839E-02
**Standard error**	1,089E-06	7,339E-04	9,7814E-02	#VALUE!	#VALUE!	#VALUE!	#VALUE!	#VALUE!	#VALUE!	9,130E-07	6,150E-04	8,1975E-02	1,444E-06	9,729E-04	1,2967E-01
**Coefficient of determination**	**0,99962**	9,172E-02	#N/A	#VALUE!	#VALUE!	#VALUE!	#VALUE!	#VALUE!	#VALUE!	**0,99969**	7,686E-02	#N/A	**0,99953**	1,216E-01	#N/A

Calibration 25-125µM

	Fucose			Rhamnose			Arabinose			Galactose			Glucose			Xylose		
	**B1**	**B2**	**c**	**B1**	**B2**	**c**	**B1**	**B2**	**c**	**B1**	**B2**	**c**	**B1**	**B2**	**c**	**B1**	**B2**	**c**
**Value**	-1,556E-06	1,273E-02	2,5883E+00	-8,060E-07	7,968E-03	4,4744E-01	-1,630E-06	1,376E-02	4,5160E-01	-2,009E-06	1,638E-02	3,0494E+00	-2,545E-06	1,963E-02	3,3704E+00	-2,094E-06	1,612E-02	1,5469E+00
**Standard error**	1,366E-07	5,221E-04	4,2818E-01	3,984E-08	1,523E-04	1,2490E-01	1,242E-07	4,748E-04	3,8942E-01	8,986E-08	3,434E-04	2,8167E-01	1,839E-07	7,028E-04	5,7640E-01	1,215E-07	4,645E-04	3,8092E-01
**Coefficient of determination**	**0,99958**	1,996E-01	#N/A	**0,99993**	5,824E-02	#N/A	**0,99972**	1,816E-01	#N/A	**0,99989**	1,313E-01	#N/A	**0,99965**	2,687E-01	#N/A	**0,99977**	1,776E-01	#N/A

	Mannose			Fructose			Apiose			GalA			GlcA		
	**B1**	**B2**	**c**	**B1**	**B2**	**c**	**B1**	**B2**	**c**	**B1**	**B2**	**c**	**B1**	**B2**	**c**
**Value**	-1,741E-06	1,587E-02	-6,8104E-01	#VALUE!	#VALUE!	#VALUE!	#VALUE!	#VALUE!	#VALUE!	-2,332E-07	1,406E-02	-6,6804E-01	-5,468E-07	1,704E-02	-1,4080E-02
**Standard error**	3,791E-07	1,449E-03	1,1885E+00	#VALUE!	#VALUE!	#VALUE!	#VALUE!	#VALUE!	#VALUE!	2,104E-07	8,041E-04	6,5944E-01	2,321E-07	8,870E-04	7,2747E-01
**Coefficient of determination**	**0,99823**	5,542E-01	#N/A	#VALUE!	#VALUE!	#VALUE!	#VALUE!	#VALUE!	#VALUE!	**0,99972**	3,075E-01	#N/A	**0,99974**	3,392E-01	#N/A

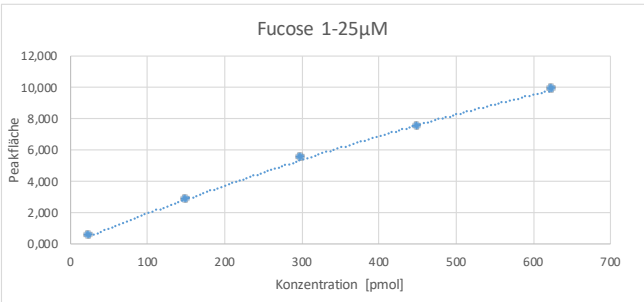

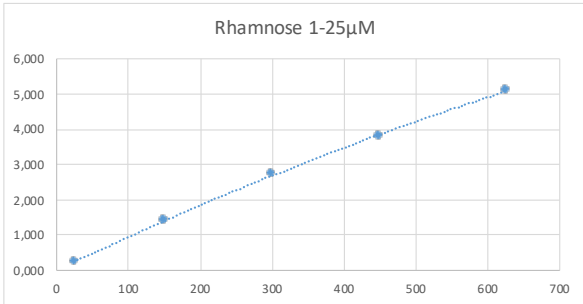

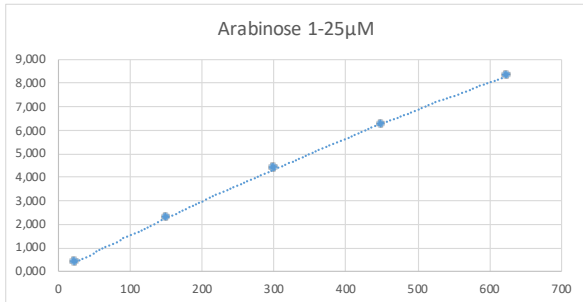

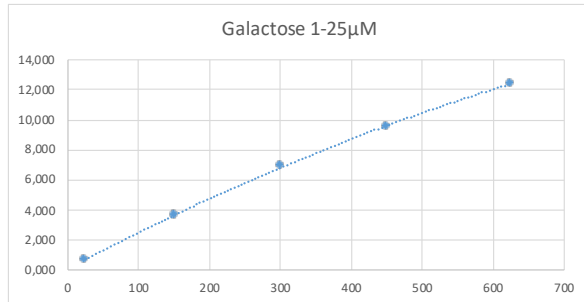

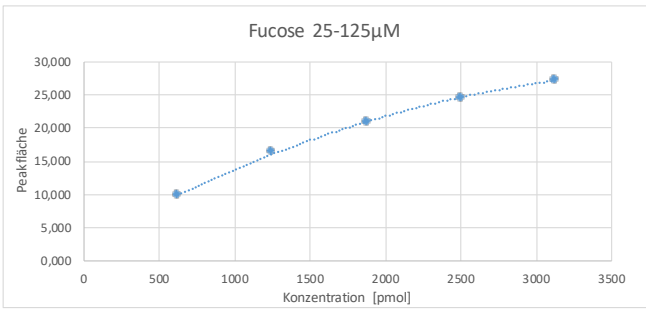

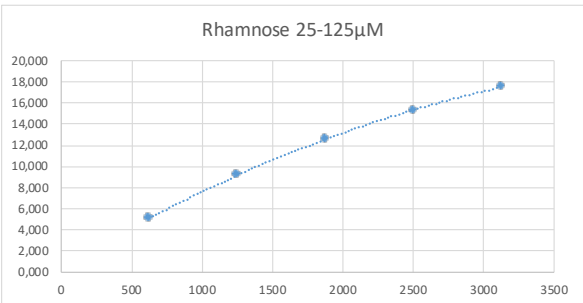

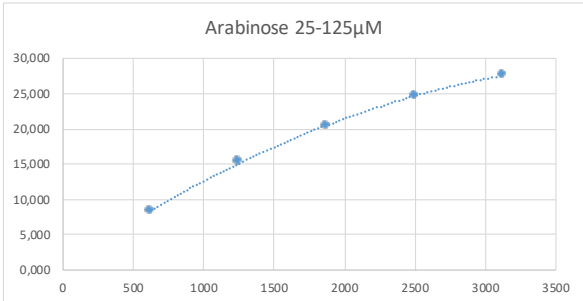

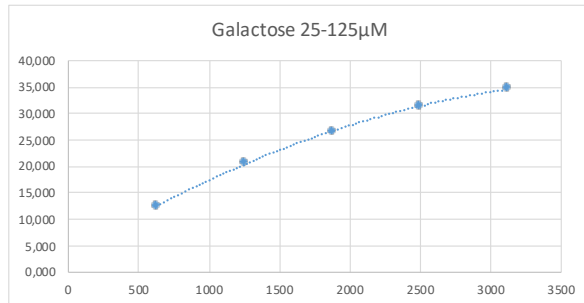

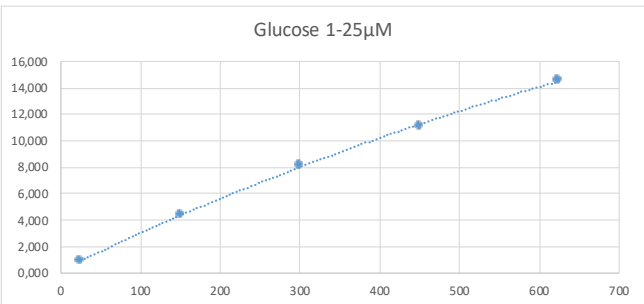

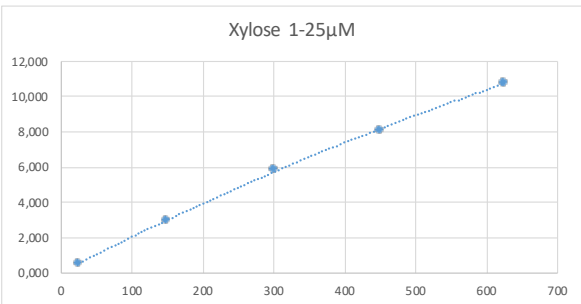

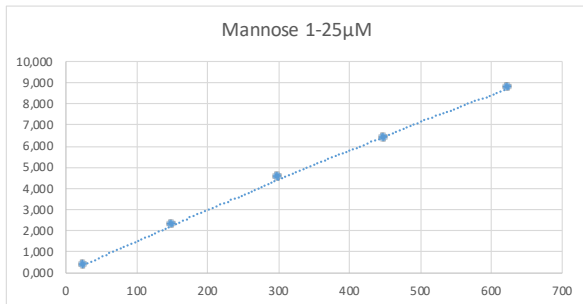

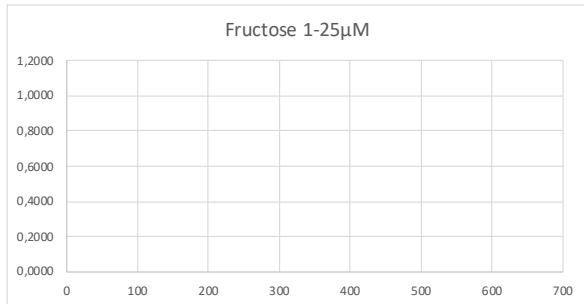

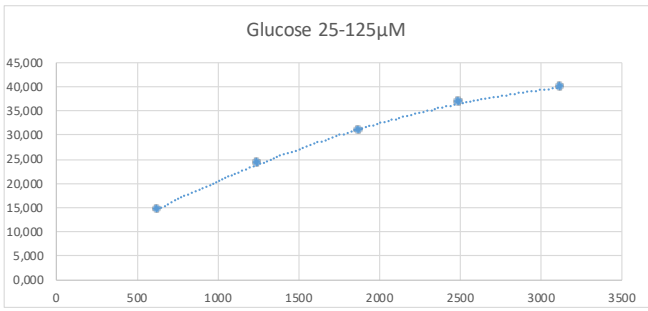

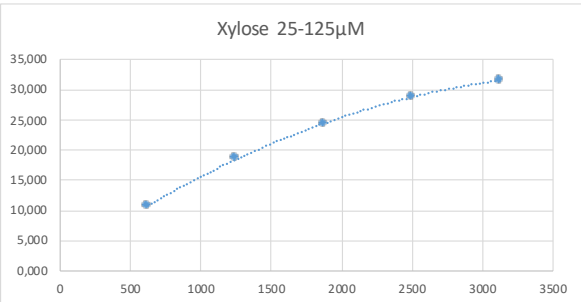

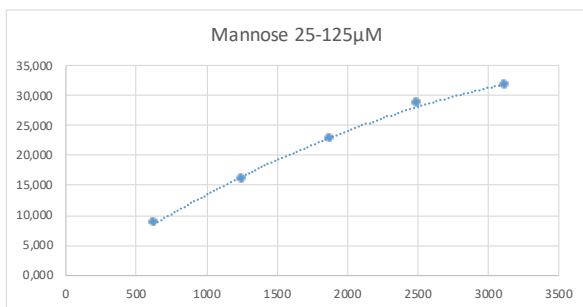

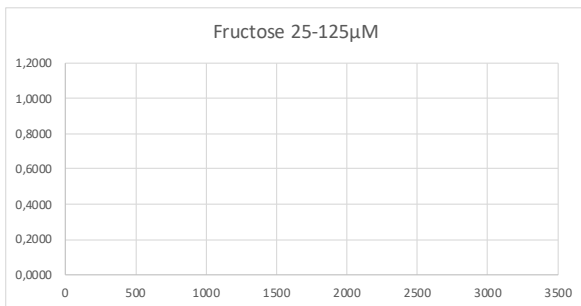

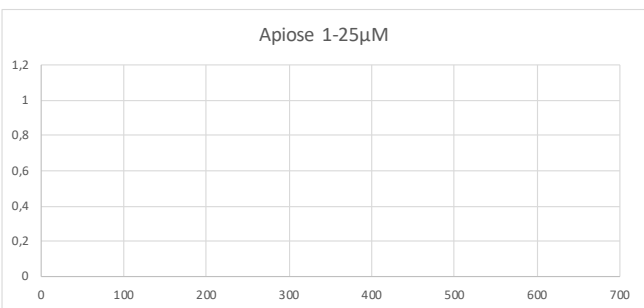

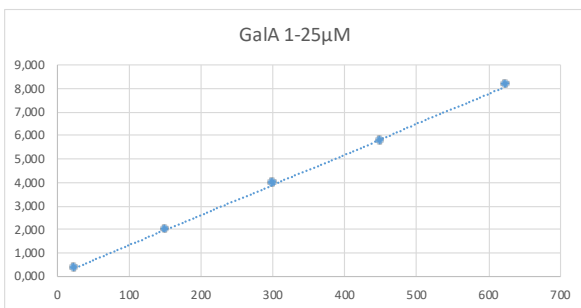

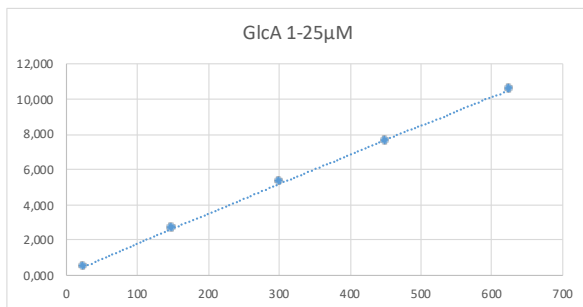

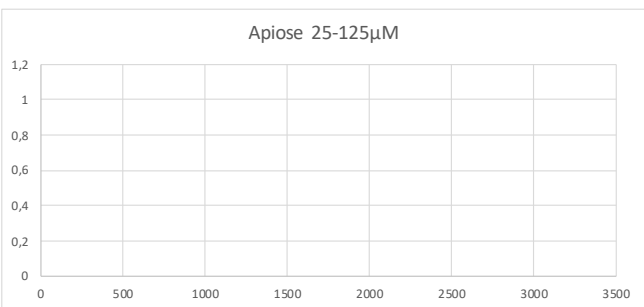

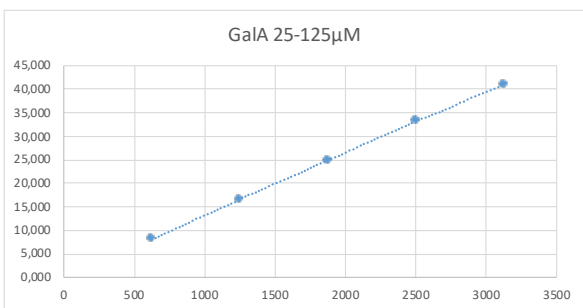

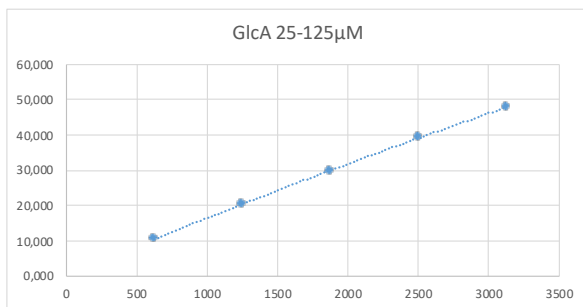

Supplement: Supplementary file 1 [file 42003_2026_10872_MOESM10_ESM.pdf]
