# Supplementary material for: Plant heteropolysaccharides and their bioadhesion to animal glycocalyx visualized at the nanoscale
Source: Commun Biol. 2026 Sep 22;9:1238. doi: 10.1038/s42003-026-10872-y (PMC13597490; doi:10.1038/s42003-026-10872-y)
Supplement: Supplementary file 2 [file 42003_2026_10872_MOESM11_ESM.pdf]

nature portfolio

Peer Review File

Plant heteropolysaccharides and their bioadhesion to animal glycocalyx visualized at the nanoscale

Corresponding Author: Dr Irene Wacker

This file contains all reviewer reports in order by version, followed by all author rebuttals in order by version.

Version 0:

Reviewer comments:

Reviewer #2

(Remarks to the Author)
The authors present a technical paper to optimise fixation/stabilisation protocols for investigating pectin films that have recently found clinical application as sealants. By incorporating ethanol into their fixation and sample preparation protocols, the authors have developed an approach to hydrate and stabilise pectin films for nano-scale visualisation through electron microscopy. This further enabled the imaging of pectin interaction with excised murine lung tissues. For the data presented, it does not appear to support the claim of entanglement between pectin strands and glycan chains from the lung epithelial tissue. The authors should consider removing definitive language for entanglement unless further tested. Proteoglycans and hyaluronic acids are long chain glycocalyx polymers present on the lung pleural surface and may play key roles in any physical entanglement with pectin films. One possible test might be through enzymatic removing glycocalyx components, e.g. hyaluronidase to partially or completely remove hyaluronic acids, that have been shown to markedly reduce pectin association with pleural surfaces (references 10 and 12 that are cited in the study). EM Imaging of pectin films on pleural surfaces that are differentially treated to remove long-chain glycan components could provide some indication of any "physical" entanglement or tight association that might occur. Any alterations also need to be carefully quantified instead of simply "stating" that entanglement is "obvious". One important consideration is whether there might be any molecular binding between pectin carbohydrate and components of the pleural surface. For example, the presence of galectins on pleural surfaces could bind galactan chains that are present in pectins. Alternatively, if the technique is not too prohibiting, the authors could also consider simplified in vitro systems of densely surface-deposited/grafted hyaluronic acid chains to interact with pectin films. EM imaging of the interactions between pectin chains and hyaluronic acids chains could indicate whether physical entanglement might occur. A general note to the authors is that there is a lack of consistency in detailing the specific experimental conditions in the main text and figure legends. It is advisable for the authors to indicate if certain standard buffer conditions are used throughout the manuscript. This could minimise inconsistency in their description and avoid confusion to the reader. There is also a general lack of detailed quantification or statistical analyses throughout the entire manuscript. The observations were mostly qualitative descriptions.

Other comments:

Lines 117, 133, 187, etc.: should capitalise Fig. for consistency throughout the article.

Line 118: the authors indicated that a combination of "ethanol, ruthenium red, and conventional aldehyde fixatives in a Pipes buffer." was used to achieve limited hydration. However, in Figure 1 legend (Line 524-525), the authors instead indicated "buffer solution containing ruthenium red and 15-40% ethanol". It is not clear whether aldehyde fixatives have been present and if so, which type. The authors have used glutaraldehyde (GA) at 2.5% elsewhere in the manuscript. Was this included in the Figure 1 example?

Line 130: "To determine the optimal hydration state for imaging a given type of pectin, here pectin from lemon, we varied the concentration of ethanol in the fixative". This sentence needs revision for clarity.

Line 130-136 in the main text document and Fig. S1/Line 103-107 in the supplemental document: the figure legend indicated that the samples were exposed to "different aqueous fixatives with 15% ethanol added for 2h in total (Fix)". Presumably this is reflected in the figure as "Fix (15% etOH)". If all the samples were exposed for 2 h in 15% ethanol, then it is not clear why the graph would indicate hydration data for two different time-points (10 min/20 min) for this same 2 h incubation. Did the

authors carry out statistical tests? Are the results significantly different between the 10 min vs 20 min incubations at the different dehydration steps? For the stated "different aqueous fixatives with 15% ethanol", what are the exact aqueous fixatives used? Were the three different pieces of film in each experiment exposed to different fixatives though all contained 15% ethanol? How are these different fixatives controlled experimentally? Was osmium tetroxide included? Note that ethanol alone can be used as a fixative. It is not clear whether the authors used 15% ethanol as the sole fixative as indicated by "Fix (15% etOH)" or in combination with other reagents. Note also that Table 1 indicated the sole use of 25% - 100% ethanol content for comparison without the addition of other reagents.

Line 137-138: the authors indicated "during embedding procedures" with a reference to "air drying". This contradicts the flow chart in Figure 1 where "air-dried" samples were not embedded in epoxy but instead directly mounted for SEM imaging. It is therefore unclear whether the authors are still referring to the same procedure as Figure 1 to include ruthenium red, osmium tetroxide, and possibly glutaraldehyde, when comparing samples in different ethanol content.

Line 153: could the authors clarify what they meant by "the fracture face of lemon pectin prepared with 15% ethanol exposed a more homogeneous aspect in overview"? Do the authors mean that it is more homogeneous than pectin prepared from other plans in comparison? Or simply that the overview provided by zoom-out imaging gave a "homogeneous" appearance? The sentence would require clarification.

Figure 2: the authors indicated that the pectin films were hydrated "in the presence of 40% or 15% ethanol". The authors should clarify whether this was in buffer (Pipes?) and whether ruthenium red, osmium tetroxide, and other aldehyde fixatives were also present that could potentially impact the solution osmolarity and therefore sample hydration. For example, Figure 3 indicated staining with ruthenium red and osmium tetroxide.

Line 321-331: the authors indicated that the pectin films were fabricated specifically for this study. Could the authors indicate whether their approach represents typical or standard methods in the literature for generating pectin films? There is currently no citation for the method employed. Do the authors observe batch-to-batch variations? If so, what are the quality control mechanisms to minimise these variations and ensure consistent production to avoid any confounding factors that could potentially impact downstream analyses?

Line 162-164: could the authors detail how they have quantified the stability of the pectin films for determining the amount of ethanol to use in the fixation step? The authors stated that potato pectin films require 40% ethanol to remain stable. However, in the methods section, it appears that the amount of ethanol is tailored only at the fixation step and all subsequent washing and staining with ruthenium red and osmium tetroxide have been carried out with 20% ethanol in 0.1M Pipes buffer. Do those that require higher ethanol content remain stable during these steps?
On a related note, how did the authors rule out structural artefacts that may be introduced by instabilities related to using ethanol at lower-than-stable concentrations? For example, in Figure 3, how do the authors rule out that the globular domains observed in potato pectin films are not due to instability introduced by switching from 40% to 20% ethanol for prolonged periods in the ensuing steps? Have the authors carried out similar 40% to 20% ethanol switch for lemon pectin films (e.g. Fig. 2)? Carboxymethylcellulose pectin films also displayed similar globular domains in Figure 3S. Were these first stabilised in 40% ethanol then switched to 20%? The authors should detail the exact ethanol concentration that has been used for pectin films from the different plants.

Line 165: have the authors considered how the different chemical compositions of pectin from different plants might impact the observed structures?

Line 167: the authors indicated that orange pectin films had higher porosity compared to those derived from soy and potato. In Figure 3, soy (Bii) and potato (Cii) showed highly dense pectin films compared to those derived from orange (Aii). Leomon pectin films exposed to 40% ethanol (similar to the potato pectin) were also highly dense (Figure 2). Note that the authors had provided ethanol concentrations for lemon and potato pectin but such information seemed to be missing for any other pectin/carboxymethylcellulose used in the study. It is therefore difficult to draw conclusions on the data presented. The authors should also indicate how they have quantified porosity and density of the pectin films beyond qualitative descriptions.

Figure 3: legend should indicate what i and ii represent.

Figure S3: legend should indicate what the arrows and arrow heads represent. Should also indicate imaging modality – e.g. A and Ai are SEM and Aii TEM images.

Line 169-178: As the varying % ethanol played a key role in stabilising pectin from different plants and can also impacted pectin film structures, how did the authors control the effect of sample processing to ensure robust observations? As above, % ethanol used for stabilisation in the fixation step needs to be stated for all pectin/carboxymethylcellulose films used in the study. It is difficult to assess the impact of sample processing if this information is not considered.

Figure S4-S7: legend should indicate imaging modality.

Lin 217: could the authors clarify what "glycocalyx globules" are? And why might they be similar to microvilli cross-section? Reference 12, as indicated, does not use the same terminology.
Line 223-236: the authors stated, "it is obvious that fine strands originating from the cell surface (Fig. 5E) are physically entangled with pectin chains". From Figure 5E, "fine strands" can be seen that may be wrapping around microvilli that are

identified by the authors (arrowheads). The authors assumed these fine strands are part of the cellular glycocalyx. However, it is not immediately clear where pectin chains are positioned in relation to these fine strands (presumed glycocalyx) and where stated “entanglement” has occurred. Further, in the FIB SEM reconstruction (Figure 5D and the associated movies), the indicated glycocalyx filaments (dark blue) are physically separated from pectin chains (light blue). Their physical separation clearly in the modelled reconstruction shows that there is no obvious entanglement that has occurred between the glycocalyx and the pectin chains. Could the authors indicate regions of the “obvious” entanglement in Figure 5E?
As a side note, there appears to be many “fine strands” that are far away from the cell surface. Following reasoning of the authors annotation in Figure 5D, the strands in Figure 5E that are far away from the cell surface would be likely to be pectin chains. It appears difficult to discern whether the stated “fine strands” are indeed part of the glycocalyx or part of the pectin film that is attached to the cell surface.

Figure 5D and 5E had the gray scales inverted. Could the authors make these consistent for comparison?

Reviewer #3

(Remarks to the Author)
This manuscript presents the the authors' findings on how to preserve pectin ultrastructure and their assessments of pectin interactions with the glycocalyx via a mouse lung in a surgical setting.
1. Since the use of pectin in lung surgery is not novel, more detailed investigations on the retention of pectin over time and maintenance of its structural integrity over time could have been investigated to further strengthen this work.
2. Please provide the molecular weights of the different types of pectins used.
3. Please provide more information on the statistical analysis done on the data obtained.
4. The rationale for using mice under lethal dose of anesthesia for testing the adhesive properties of the film is unclear, as the breathing rate and other body functions would have slowed down. Assessing bioadhesiveness in the presence of regularly exerted mechanical stresses may have been better.
5. The abstract could be further strengthened by describing in more detail the key findings from the studies in the manuscript.
6. The potential applications of the pectin films and future work to be carried out could have been described more clearly.

Reviewer #4

(Remarks to the Author)
The work is interesting and valuable, although several aspects related to the characteristics of the pectins used in the studies and the conditions of their cross-linking and gelation should be explained in more detail. The manuscript requires major revision.
L 323 Pectin must be chemically characterized – monosaccharide composition, DM, DA, /Mw. These parameters influence mainly the cross-linking processes.
L 328 How long was the dissolution of pectin carried out? It has a crucial role in the spatial arrangement of pectin molecules.
L 330 Describe the method of pectin film preparation with details, the amount of pectin sample, the temperature of drying, the time of drying, or the drying conditions.

L 234-235 Pectin is a very complex family of polysaccharides; it can be divided into several main domains (homogalacturonan, rhamnogalacturonans), and their chemical composition, molecular properties, structure, and thusability to absorption depend strongly on source, extraction method, and even the physiological stage of the fruit. So, the term ‘pure pectin’ is highly imprecise. In the experiment, no information was provided regarding these critical factors. In my opinion, it is not possible to analyze the results without considering these facts.
236 Pecin gelling ability depends mainly on the number of methyl esters of carboxyl groups of galacturonic acid. The mechanism of gelling should be explained, especially given that calcium ions were applied along with a high sucrose concentration and low pH.

Version 1:

Reviewer comments:

Reviewer #2

(Remarks to the Author)
The authors have appropriately and thoroughly addressed the comments and concerns raised. Missing details to analyses and experimental set-up are now listed. The manuscript has been appropriately revised to remove previous unsupported conclusion regarding entanglement.

Reviewer #3

(Remarks to the Author)
The updated manuscript is responsive to prior comments.

Reviewer #4

(Remarks to the Author)
The manuscript has been significantly revised and expanded. The pectin used in the study has been chemically analyzed, and the methodology has been described. However, I would suggest making the following minor corrections:
Table S3 – please use the same number of significant digits.
Table S5 – please add standard error values.
Figure S4 – please add a discussion of the SEC analysis results; it is difficult to determine the molecular weight from the graph alone.
Please check the response to the comment: “236 Pecin’s gelling ability depends mainly on the number of methyl esters of the carboxyl groups of galacturonic acid. The mechanism of gelling should be explained, especially given that calcium ions were applied along with a high sucrose concentration and low pH.” It appears the response is incomplete since it ends with a colon (:).

Open Access This Peer Review File is licensed under a Creative Commons Attribution 4.0 International License, which permits use, sharing, adaptation, distribution and reproduction in any medium or format, as long as you give appropriate credit to the original author(s) and the source, provide a link to the Creative Commons license, and indicate if changes were made.
In cases where reviewers are anonymous, credit should be given to 'Anonymous Referee' and the source.
The images or other third party material in this Peer Review File are included in the article’s Creative Commons license, unless indicated otherwise in a credit line to the material. If material is not included in the article’s Creative Commons license and your intended use is not permitted by statutory regulation or exceeds the permitted use, you will need to obtain permission directly from the copyright holder.
To view a copy of this license, visit https://creativecommons.org/licenses/by/4.0/

Response to referees

Response to reviewer#1

Note: when line numbers are given here by us, they refer to the new MS version with tracking.
New text is shown in orange

The authors present a technical paper to optimise fixation/stabilisation protocols for investigating pectin films that have recently found clinical application as sealants. By incorporating ethanol into their fixation and sample preparation protocols, the authors have developed an approach to hydrate and stabilise pectin films for nano-scale visualisation through electron microscopy. This further enabled the imaging of pectin interaction with excised murine lung tissues. For the data presented, it does not appear to support the claim of entanglement between pectin strands and glycan chains from the lung epithelial tissue. The authors should consider removing definitive language for entanglement unless further tested.
We understand the reviewer's concerns and tuned down all statements regarding proof of entanglement accordingly, starting with the MS title, and also in the abstract, results section (line 429ff), and discussion (line 571).
Proteoglycans and hyaluronic acids are long chain glycocalyx polymers present on the lung pleural surface and may play key roles in any physical entanglement with pectin films. One possible test might be through enzymatic removing glycocalyx components, e.g. hyaluronidase to partially or completely remove hyaluronic acids, that have been shown to markedly reduce pectin association with pleural surfaces (references 10 and 12 that are cited in the study= Servais 1/3). EM Imaging of pectin films on pleural surfaces that are differentially treated to remove long-chain glycan components could provide some indication of any "physical" entanglement or tight association that might occur. Any alterations also need to be carefully quantified instead of simply "stating" that entanglement is "obvious". One important consideration is whether there might be any molecular binding between pectin carbohydrate and components of the pleural surface. For example, the presence of galectins on pleural surfaces could bind galactan chains that are present in pectins.
We appreciate the the constructive suggestion from Reviewer #1. We agree that testing adhesion after removal of structural components of the glycocalyx would further strengthen the overall body of work. However, we respectfully note that the scope of the present study was intentionally limited to establishing feasibility, specifically demonstrating that a pectin patch can be successfully retained on the organ surface and remain intact through the demanding dehydration and embedding steps required for electron microscopy (EM) preparation. We have clarified this point in the revised text to emphasize that the goal of this paper is methodological feasibility and structural preservation through EM processing.
Alternatively, if the technique is not too prohibiting, the authors could also consider simplified in vitro systems of densely surface-deposited/grafted hyaluronic acid chains to interact with pectin films. EM imaging of the interactions between pectin chains and hyaluronic acids chains could indicate whether physical entanglement might occur.
We appreciate this suggestion, but from our experience with hydrogels we know that the initial problem of the present work, the problem of identification "who is who" would not be solved by such an in vitro approach. How would we distinguish the HA from pectin? We have images of cells in HAMA hydrogels and also of 0.32% HAMA gels alone – one sees short filaments and globules, similar to what we visualized for some pectic materials after limited hydration. Unless we would be able to attach a specific label to one partner of the proposed interaction complex, we could not reach a valid interpretation. And such an extensive labeling study is definitely beyond the scope of the present work.
A general note to theauthors is that there is a lack of consistency in detailing the specific experimental conditions in the main text and figure legends. It is advisable for the authors to indicate if certain standard buffer conditions are used throughout the manuscript. This could minimise inconsistency in their description and avoid confusion to the reader.
We apologize for this confusion. We now included a table in SI (table S2) where all experimental conditions are listed for all EM images shown. We also added experimental conditions to the figure legends where appropriate.

There is also a general lack of detailed quantification or statistical analyses throughout the entire manuscript. The observations were mostly qualitative descriptions.

Gravimetric measurements during embedding procedures for EM were done for 1 sample only, because otherwise it would not have been possible to keep consistent time intervals for the different incubation steps. To check how reliable the weighing of such small pieces (weight ca 10mg) could be performed we did one experiment with three independent pieces of material, which were weighed as dry films, then after the entire incubation series in the primary/secondary fixatives including the washing steps in between (initially in 15%, then 20% ethanol) plus after each incubation step during the ascending ethanol series necessary for subsequent dehydration prior to embedding in hydrophobic resin. This is shown in the new Figure S2 as mean with standard deviation for two different dehydration schemes: 10 min/step or 20 min/step. We also explained the experimental setup in more detail in the figure legend.

Furthermore, we included a scatter plot for hydration factors vs ethanol concentration for all pectic polysaccharides (PPS) described in the MS. Since lemon pectin was always used as a reference, values from several different experiments are shown for that.

Although the aim of the present work was not a thorough morphological characterization of the different materials, we now also performed image analysis to assess porosity and network characteristics of the three PPS compared in Figure 5 of the MS. Corresponding data and explanation are given on page 14 & 15 of the SI.

Other comments:

Lines 117, 133, 187, etc.: should capitalise Fig. for consistency throughout the article.

We capitalized all occurences of Fig. and Table.

Line 118: the authors indicated that a combination of “ethanol, ruthenium red, and conventional aldehyde fixatives in a Pipes buffer.” was used to achieve limited hydration. However, in Figure 1 legend (Line 524-525), the authors instead indicated “buffer solution containing ruthenium red and 15-40% ethanol”. It is not clear whether aldehyde fixatives have been present and if so, which type. The authors have used glutaraldehyde (GA) at 2.5% elsewhere in the manuscript. Was this included in the Figure 1 example?

We apologize for the misleading wording and rephrased:

MS text, line 189: limited hydration is achieved by combining a fixative consisting of 2.5% glutaraldehyde and 0.15% ruthenium red in 0.1M Pipes buffer with ethanol. The concentration of ethanol depends on the pectin type used and can vary from 15% to 40%. All aqueous solutions used in the following steps, including washing buffers, contained at least the initial concentration of ethanol (cf table S2).

Legend to Fig.1/new Fig.2: Glass phase pectin films (A) are partially hydrated (B) in 0.1M Pipes buffer containing 2.5% glutaraldehyde as fixative, 0.15% ruthenium red and 15-40% ethanol (exact value depending on the type of pectin).

Line 130: “To determine the optimal hydration state for imaging a given type of pectin, here pectin from lemon, we varied the concentration of ethanol in the fixative”. This sentence needs revision for clarity.

We apologize for the misleading wording and rephrased:

To determine the optimal hydration state for imaging a given type of pectin we varied the concentration of ethanol in the fixative (cf table S1) and all consecutive solutions (cf table S2) and determined water uptake by gravimetric analysis (Fig. S1).

Line 130-136 in the main text document and Fig. S1/Line 103-107 in the supplemental document: the figure legend indicated that the samples were exposed to “different aqueous fixatives with 15% ethanol added for 2h in total (Fix)”. Presumably this is reflected in the figure as “Fix (15% etOH)”. If all the samples were exposed for 2 h in 15% ethanol, then it is not clear why the graph would indicate hydration data for two different time-points (10 min/20 min) for this same 2 h incubation.

To check how reliable the weighing of such small pieces (weight ca 10mg) could be performed we did one experiment with three independent pieces of material, which were weighed as dry films, then after the entire incubation series in the primary/secondary fixatives including the washing steps in between (initially in 15%, then 20% ethanol) plus after each incubation step during the ascending ethanol series necessary for subsequent dehydration prior to embedding in hydrophobic resin. This is shown in Figure S2 as mean with standard deviation for two different dehydration schemes: 10 min/step or 20 min/step.

We rephrased the main text, line 208:

To illustrate the reliability of this gravimetric analysis, fig. S2 shows the dehydration kinetics of lemon pectin after limited hydration in fixative.

The figure legend now contains a full description of the experiment:
Fig. S2 Dehydration kinetics of lemon pectin films with 10 min or 20 min incubation time during dehydration steps. For each experiment three pieces of film were weighed initially as dry material. They were then incubated for 30 min with the primary fixative (2.5% GA, 0.15% Ruthenium Red, 15% ethanol in 0.1M Pipes), washed 3x5 min with 20% ethanol in 0.1M Pipes, incubated 1h in a mimic of the secondary fixative (0.15% ruthenium red, 20% ethanol in 0.1M Pipes), washed again 3x5 min with 20% ethanol in 0.1M Pipes, and weighed a second time ("Fix"). After that they were dehydrated in a graded ethanol series followed by acetone with 10 min or 20 min incubation time for each step. During that interval they were weighed after each dehydration step. All values were normalized to dry weight (= hydration factor, HF) and are plotted as average ± SD with the original data points inserted as dots. The statistical analysis shows, that the individual pairs (10/20 min experimental method) overlap within a 1σ combined uncertainty, thus would not indicate a significant difference. However, the 10min experiment values are consistently higher than the 20min values (6 out of 7 data points) with an average difference of +0.76 (not excluding the one outlier) with a standard deviation of the difference of 0.72. The offset is therefore +0.76 +/- 0.72, which includes zero only at the 2σ level, i.e. the 10min experiments show a systematic higher value.

Did the authors carry out statistical tests? Are the results significantly different between the 10 min vs 20 min incubations at the different dehydration steps?

See new figure legend S2 above

For the stated "different aqueous fixatives with 15% ethanol", what are the exact aqueous fixatives used?

1st fix 2.5% GA, 0.15% RR, 15% etOH, 0.1MPipes x 30min
2nd fix 0.15% RR, 20% etOH, 0.1M Pipes x 1h
See new figure legend S2 above

Were the three different pieces of film in each experiment exposed to different fixatives though all contained 15% ethanol?

No, see new figure legend S2 above

How are these different fixatives controlled experimentally?

Fixatives were always primary fixative with GA, and secondary fixative with OsO_4, always in 0.1M Pipes. RR was always added to primary and secondary fixative, but not to the washing solutions.

Was osmium tetroxide included? Note that ethanol alone can be used as a fixative. It is not clear whether the authors used 15% ethanol as the sole fixative as indicated by "Fix (15% etOH)" or in combination with other reagents.

This is now explained in the legend to Fig. S2, and in the corresponding note, page 6 of SI

Note also that Table 1 indicated the sole use of 25% - 100% ethanol content for comparison without the addition of other reagents.

Table 1 represents an initial test how the different solutions used in a canonical EM fixation procedure would interact with our pectin films. Since the entire "limited hydration" exercise was aimed at the final proof of concept – the embedding of a pectin-film-on-tissue sandwich – we always needed to have the requirements of tissue preservation in mind. For that reason, in all consecutive experiments combined fixative solutions (fixans + stain + buffer + ethanol) were used.

Line 137-138: the authors indicated "during embedding procedures" with a reference to "air drying". This contradicts the flow chart in Figure 1 where "air-dried" samples were not embedded in epoxy but instead directly mounted for SEM imaging. It is therefore unclear whether the authors are still referring to the same procedure as Figure 1 to include ruthenium red, osmium tetroxide, and possibly glutaraldehyde, when comparing samples in different ethanol content.

We always used the same components in the fixatives: 0.1M Pipes, 2.5% GA, 0.15% RR plus ethanol which was varied in concentration to keep the respective pectin type in the desired hydration status. When we did an embedding experiment we used large pieces of film for the weighing part – those were then air-dried and fractured. In parallel we processed smaller pieces (too small to be reliably weighed) for embedding in resin to achieve good infiltration and sectioning results.

We rephrased, line 210:

During embedding procedures, in our canonical fixative (2.5% glutaraldehyde, 0.15% ruthenium red in 0.1M Pipes buffer) plus added ethanol weights were only obtained after the initial fixation ("hydrated"),

Line 153: could the authors clarify what they meant by "the fracture face of lemon pectin prepared with 15% ethanol exposed a more homogeneous aspect in overview"? Do the authors mean that it is more homogeneous than pectin prepared from other plans in comparison? Or simply that the overview provided by zoom-out imaging gave a "homogeneous" appearance? The sentence would require clarification.

We rephrased, line 247
The fracture face of lemon pectin prepared with 20% ethanol was devoid of layering when comparing the overview (Fig. S6A) with the fracture face of lemon pectin prepared with 40% ethanol (cf Fig. 3Ai). Higher magnifications showed a fibrous network for both, fracture face (Fig. S6Ai) and cross section (Fig. S6Aii).
Figure 2: the authors indicated that the pectin films were hydrated "in the presence of 40% or 15% ethanol". The authors should clarify whether this was in buffer (Pipes?) and whether ruthenium red, osmium tetroxide, and other aldehyde fixatives were also present that could potentially impact the solution osmolarity and therefore sample hydration. For example, Figure 3 indicated staining with ruthenium red and osmium tetroxide.
We rephrased the first sentence of the figure legend (new figure 3):
Pectin films (lemon) were either hydrated in the presence of 40% (A) or 15% (B) ethanol in a fixative consisting of 2.5% GA, 0.15% RR in 0.1M Pipes.
Line 321-331: the authors indicated that the pectin films were fabricated specifically for this study. Could the authors indicate whether their approach represents typical or standard methods in the literature for generating pectin films?
We thank the reviewer for this question. The pectin films used in this study were fabricated specifically for the experiments described; however, the fabrication approach itself follows well-established and widely used methods in the pectin literature. In particular, our process is based on conventional aqueous dissolution of pectin, controlled ionic conditions, solution casting, and ambient drying, which represent the standard methodology for generating pectin films in both food science and biomaterials research.
Accordingly, while the films were produced de novo for this study, their composition, structure, and fabrication are representative of standard pectin film preparation methods reported in the literature. This approach ensures that the observed behaviors reflect intrinsic pectin network properties rather than artifacts of an atypical or proprietary fabrication process.
There is currently no citation for the method employed.
The relationship between polymer mechanics and hydration was illustrated in our technical paper: Pierce A, Zheng Y, Wagner WL, Scheller HV, Mohnen D, Tsuda A, Ackermann M, Mentzer SJ (2020) Pectin biopolymer mechanics and microstructure associated with polysaccharide phase transitions. J Biol Mat Res Part A 108:246–253.
This is now cited in line 616, (section fabrication of pectin films) as ref 34
Do the authors observe batch-to-batch variations? If so, what are the quality control mechanisms to minimise these variations and ensure consistent production to avoid any confounding factors that could potentially impact downstream analyses?
We acknowledge the reviewer's concern regarding potential batch-to-batch variability. As with all naturally derived polysaccharides, pectin can exhibit inherent compositional heterogeneity. In our experience, batch-to-batch variation is minimal and well controlled when material is sourced, processed, and formulated under standardized conditions.
In addition, our extensive chemical analysis of two films from different lemon-derived batches indicates that monosaccharide composition, molecular weight, DM and DA are indeed comparable (see tables S3-S5 in SI and extended new text in results section of MS).
To minimize variability and prevent confounding effects in downstream analyses, we employ multiple quality control (QC) measures at both the raw material and final film levels. These include: (i) compositional verification of each batch (galacturonic acid content and degree of methylation), (ii) control of ionic conditions and calcium content during formulation, (iii) standardized dissolution, casting, and drying protocols, and (iv) exclusion of batches that fall outside predefined acceptance criteria. Functional QC metrics—including swelling behavior, mechanical integrity, and adhesion performance—are also assessed to confirm consistency of the formed films.
Importantly, our downstream analyses rely on within-batch controls and replicated experiments across independent batches, ensuring that observed effects are robust and not driven by batch-specific artifacts. Collectively, these measures ensure reproducible material properties and consistent performance, while minimizing the impact of unavoidable natural variability inherent to pectin-based systems.
Line 162-164: could the authors detail how they have quantified the stability of the pectin films for determining the amount of ethanol to use in the fixation step?
We actually did determine hydration factors for combinations of fixative components with a given concentration of ethanol. Having learned that hydration factors below 5 did produce only very compact morphologies with little structural detail and with factors above 20 films tended to dissolve we aimed for hydration factors between 10 and 20. Apart from that we did no further quantification, we

just aimed to preserve a film that could be carried through an entire embedding procedure for EM. For potato pectin we also tested the different components of the fixative solution single and in combinations and found that only in the full set of GA, RR, Pipes plus 40% ethanol the film would not disintegrate. For potato pectin the secondary fixative contained 50% ethanol, all washing solutions were kept at 40% ethanol in 0.1M Pipes.

The authors stated that potato pectin films require 40% ethanol to remain stable. However, in the methods section, it appears that the amount of ethanol is tailored only at the fixation step and all subsequent washing and staining with ruthenium red and osmium tetroxide have been carried out with 20% ethanol in 0.1M Pipes buffer. Do those that require higher ethanol content remain stable during these steps?

We apologize for that omission and have added a sentence to material and methods section, line 723: For potato pectin, a higher concentration of ethanol was required to keep a stable film during the embedding procedure. Table S2 summarizes the exact ethanol concentrations for all PPS during all incubation steps in aqueous medium.

On a related note, how did the authors rule out structural artefacts that may be introduced by instabilities related to using ethanol at lower-than-stable concentrations? For example, in Figure 3, how do the authors rule out that the globular domains observed in potato pectin films are not due to instability introduced by switching from 40% to 20% ethanol for prolonged periods in the ensuing steps?

We never lowered the ethanol concentration after fixation. On the contrary, to compensate the drop in osmolarity by the lack of GA and RR in the washing solutions we usually increased ethanol concentration by 5% for washing and osmication steps (see table S2).

Have the authors carried out similar 40% to 20% ethanol switch for lemon pectin films (e.g. Fig. 2)?

There was no switch, see above

Carboxymethylcellulose pectin films also displayed similar globular domains in Figure 3S. Were these first stabilised in 40% ethanol then switched to 20%?

No they were not, see table S2

The authors should detail the exact ethanol concentration that has been used for pectin films from the different plants.

This is all summarized now in table S2.

Line 165: have the authors considered how the different chemical compositions of pectin from different plants might impact the observed structures?

Indeed, we have done so now – with all pectic polysaccharides (PPS) used in the study analyzed in much more detail, cf lines 254-312 plus Figures 4, S4 & S5 as well as tables S3-S5 – we were able to infer some correlations between chemical structure and morphology. PPS with both, hairy and smooth domains (those extracted from lemon, orange, or – to some extent – soy) showed a more filamentous morphology while those without smooth domains (potato and sugar beet which are basically modified RG1 domains) exhibited a more globular appearance. This can be found in the results section of the revised MS (lines 320-353) and also in the revised abstract. Analysis of PSDs and fractal dimensions (Fig. S7 and page 14 in SI) also demonstrate this in a more quantitative manner.

Line 167: the authors indicated that orange pectin films had higher porosity compared to those derived from soy and potato. In Figure 3, soy (Bii) and potato (Cii) showed highly dense pectin films compared to those derived from orange (Aii). Leomon pectin films exposed to 40% ethanol (similar to the potato pectin) were also highly dense (Figure 2). Note that the authors had provided ethanol concentrations for lemon and potato pectin but such information seemed to be missing for any other pectin/carboxymethylcellulose used in the study. It is therefore difficult to draw conclusions on the data presented.

We rephrased Fig. 3 legend, line 1020:

Samples were prepared with 15% ethanol in the fixative and 20% in subsequent solutions for (A, B) and with 40% ethanol in fixative and 50% in subsequent solutions for C.

We inserted the following sentence into Fig. S6 legend

All samples were prepared with ethanol in the fixative (20%) and all subsequent solutions (25%).

The authors should also indicate how they have quantified porosity and density of the pectin films beyond qualitative descriptions.

We added a new section to SI, page 14: "Assessing porosity and network characteristics of different pectic polysaccharides by Power Spectral Densities (PSD) and fractal dimension as quantitative descriptors", explaining what type of image analysis was done and a new figure S7 showing data for orange, soy, and potato.

Figure 3: legend should indicate what i and ii represent.

New legend for Figure 5 (old 3)

Pectic polysaccharides from different plants show different morphologies. Fracture faces from pectin films air-dried after staining with ruthenium red and OsO4 (i) or ultrathin cross sections from films embedded in epoxide resin after staining (ii) of pectic polysaccharides isolated from orange (A), soy (B), or potato (C). Samples were prepared with 15% ethanol in the fixative and 20% in subsequent solutions for (A, B) and with 40% ethanol in fixative and 50% in subsequent solutions for (C). Scale bars are 1 μm (top) or 400 nm (bottom).

Figure S3: legend should indicate what the arrows and arrow heads represent. Should also indicate imaging modality – e.g. A and Ai are SEM and Aii TEM images.

Actually, all images, fracture faces as well as cross sections were recorded in an SEM. We routinely image ultrathin sections on Si wafer pieces in our SEM - with a rather low primary energy of 1.5keV.

New legend for Figure S6 (old S3):

Fig. S6 Ultrastructural signatures of different polysaccharides: (A) lemon pectin, (B) lemon pectin + carboxymethylcellulose (CMC), (C) sugar beet pectin + CMC, (D) CMC. All samples were prepared with ethanol in the fixative (20%) and all subsequent solutions (25%). Overviews (A, B, C, D) of fracture faces (asteriscs) from air-dried films stained with ruthenium red and OsO_4 and high magnification images (Ai, Bi, Ci, Di) were recorded using secondary electrons. Cross sections from films embedded in epoxide after staining as above (Aii, Bii, Cii, Dii) were imaged with backscattered electrons in a SEM. Arrowheads point to globular components, arrows to filamentous structures.

Line 169-178: As the varying % ethanol played a key role in stabilising pectin from different plants and can also impacted pectin film structures, how did the authors control the effect of sample processing to ensure robust observations? As above, % ethanol used for stabilisation in the fixation step needs to be stated for all pectin/carboxymethylcellulose films used in the study. It is difficult to assess the impact of sample processing if this information is not considered.

We clarified the ethanol concentrations used for stabilization of the different pectins for all solutions utilized during the embedding in table S2. The fact that all PPS in figure S6 were treated with the same ethanol concentration, processed at the same day, and still showed different morphologies rules out a strong influence of our preparation protocol. Furthermore, lemon pectin which we always used as a control when changing ethanol concentration for the embedding protocol, never showed globular structures at high ethanol concentrations, it just became denser.

Figure S4-S7: legend should indicate imaging modality.

The title of the section was modified:

Quality of tissue preservation – SEM images of ultrathin sections

Lin 217: could the authors clarify what “glycocalyx globules” are? And why might they be similar to microvilli cross-section? Reference 12, as indicated, does not use the same terminology.

We apologize for this error, the reference # should be 18. “MPS bodies” are defined there in the results and discussion sections: “an underlying branched-chain structure with beaded periodicity (MPS bodies)”. This may actually have resulted from an inadequate preparation protocol: 24h submerged in 1% RR. According to the datasheet of RR supplier Abcam, 10mM = ca 0.75% is actually the maximum concentration at which RR is fully soluble in aqueous solution, so probably unspecific aggregates were formed here.

Although we never observed a beaded **periodicity** with our preparation protocol during which RR was used at 0.15% for a maximum of 2h (0.5-1h in primary fixative plus 1h in secondary fixative) we still observed material clusters/agglomerations of rather high electron density alternating with filamentous regions with lower electron densities (cf Fig 7c and also 7E left side). Depending on the sectioning plane, microvilli might also display an uneven/variable/irregular shape – neither exactly round nor elongated. That means, in a single image it might be difficult to distinguish a “strange” microvillus cross section from agglomerated polysaccharide fibers.

Line 223-236: the authors stated, “it is obvious that fine strands originating from the cell surface (Fig. 5E) are physically entangled with pectin chains”. From Figure 5E, “fine strands” can be seen that may be wrapping around microvilli that are identified by the authors (arrowheads). The authors assumed these fine strands are part of the cellular glycocalyx. However, it is not immediately clear where pectin chains are positioned in relation to these fine strands (presumed glycocalyx) and where stated “entanglement” has occurred.

We apologize for this blunder, indeed we have no way to decide which of the fine strands we are observing in the entire volume are pectin and which are parts of the glycocalyx. For that it would be necessary to label one partner with a specific stain as discussed above. We just made the assumption that seeing a particular strand connected to the cell surface would most probably identify it as part of the glycocalyx. Of course, we cannot rule out that it might be pectin extending that far.

Further, in the FIB SEM reconstruction (Figure 5D and the associated movies), the indicated glycocalyx filaments (dark blue) are physically separated from pectin chains (light blue).

Their physical separation clearly in the modelled reconstruction shows that there is no obvious entanglement that has occurred between the glycocalyx and the pectin chains.

When annotating the data, we made an arbitrary distinction between strands originating from the cell surface and/or the microvilli (blue) and extending no further than the microvilli on one hand and strands outside this defined region (light blue) on the other hand. They do not look much different, so they could be either pectin or glycocalyx or both. That might support the idea of entanglement, but of course does not prove it.

Could the authors indicate regions of the "obvious" entanglement in Figure 5E? As a side note, there appears to be many "fine strands" that are far away from the cell surface.

Again, we apologize for this provocative interpretation, it is exactly true that due to the similar morphology of the two materials we cannot identify a given polymer species nor delineate clearly a transition zone where they would both be present.

Following reasoning of the authors annotation in Figure 5D, the strands in Figure 5E that are far away from the cell surface would be likely to be pectin chains. It appears difficult to discern whether the stated "fine strands" are indeed part of the glycocalyx or part of the pectin film that is attached to the cell surface.

This is very true and we therefore rewrote that section completely, line 428:

For annotation of this dark blue material we only selected fine strands originating from the cell surface or from the surface of the microvilli (as e.g. shown in Fig. 7E).

Filamentous structures outside the region defined by the microvilli are annotated in light blue (see also movies_4a-c for different angles of the 3D rendering and segmentation). It is obvious that there are no morphological differences between the two annotated networks, but it is also clear that at some distance from the cell surface the pectin patch must start. However, without sugar domain specific labeling it is not possible to identify the transition between glycocalyx and pectin patch.

Figure 5D and 5E had the gray scales inverted. Could the authors make these consistent for comparison?

We agree with the reviewer, the gray scales are inverted but for simple reasons:

Figure 5E shows a slice projection from slices of the original nano-tomogram (movie 3). When trying to show the fine structures of glycocalyx and pectin at best contrast, we found an inverted gray scale (as used in the current Figure 5E) to work best. This would also be consistent with all the other shown SEM images.

However, when visualizing the 3D volume in the used software (chimeraX), the opposite is true! Without prior segmentation and annotation the currently "invisible" void volume (white in the projection image 5E) would show also as white voxels, completely obscuring the fine structures. Of course, this could be cured by only showing the segmentation, but we thought that this defeats the purpose of the movie. We therefore kept the original nano-tomogram contrast also for Figure 5D and movie 4.

Response to reviewer #2

Note: when line numbers are given here by us, they refer to the new MS version with tracking.
New text is shown in orange

This manuscript presents the the authors' findings on how to preserve pectin ultrastructure and their assessments of pectin interactions with the glycocalyx via a mouse lung in a surgical setting.

1. Since the use of pectin in lung surgery is not novel, more detailed investigations on the retention of pectin over time and maintenance of its structural integrity over time could have been investigated to further strengthen this work.

We appreciate the Editor's guidance and the constructive suggestion from Reviewer #2. We agree that detailed investigations into long-term pectin retention and structural integrity would further strengthen the overall body of work. However, we respectfully note that the scope of the present study was intentionally limited to establishing feasibility, specifically demonstrating that a pectin patch can be successfully retained on the organ surface and remain intact through the demanding dehydration and embedding steps required for electron microscopy (EM) preparation.

These EM preparation steps impose extreme chemical, osmotic, and mechanical stresses that far exceed those encountered in physiological settings. Preservation of patch integrity under these conditions therefore represents a stringent and relevant feasibility benchmark. Systematic studies of longer-term in vivo retention, degradation kinetics, and structural evolution over time are important and are the subject of ongoing and future investigations, but were beyond the focused objectives of this manuscript.

We have clarified this point in the revised text to emphasize that the goal of this paper is methodological feasibility and structural preservation through EM processing, rather than comprehensive evaluation of long-term material performance.

2. Please provide the molecular weights of the different types of pectins used.

Molecular weight distributions for the different pectic polysaccharides used are now shown in Figure S4 and are discussed in detail in the results section (chemical analysis by our new collaborators, now also added as authors).

3. Please provide more information on the statistical analysis done on the data obtained.

With all due respect, our gravimetric analysis during embedding procedures which allows us to give at least a rough estimate of the initial hydration state of the sample we are going to visualise in the end is much more than most people working with hydrogels would do. Usually samples are somehow frozen, freeze dried and then imaged with loads of artefacts - such as macropores - introduced during such a preparation. The interpretations deduced from such a (far from natural) state are often hard to believe for someone working in the field of cryo-electronmicroscopy (ice crystal damage…). Nevertheless, for the gravimetric analyses we now added a scatter plot (Figure S1) for hydration factors vs ethanol concentration for all pectic polysaccharides described in the MS. Since lemon pectin was always used as a reference, values from several different experiments are shown. Figure S2 – data for 2 experimental conditions with n=3 samples/condition - gives a reasonable estimate how reliable the gravimetric measurements could be performed. Details are given in the figure legends.

4. The rationale for using mice under lethal dose of anesthesia for testing the adhesive properties of the film is unclear, as the breathing rate and other body functions would have slowed down. Assessing bioadhesiveness in the presence of regularly exerted mechanical stresses may have been better.

We appreciate the reviewer's thoughtful suggestion. We agree that evaluating bioadhesiveness under

5. The abstract could be further strengthened by describing in more detail the key findings from the studies in the manuscript.

We agree with this suggestion, and modified the abstract accordingly, line 44 (new MS with tracking):

Depending on actual hydration states and pectic domains selected we find distinct morphologies: Citrus pectic polysaccharides, combining both, smooth homogalacturonan and hairy rhamnogalacturonan domains, form a loose network of thin filaments. Rhamnogalacturonan and xylogalacturonan domains from soy show a denser mesh, while pectic galactan from potato or pectic arabinan from sugar beet display more globular aspects.

6. The potential applications of the pectin films and future work to be carried out could have been described more clearly.

As stated before:

Systematic studies of longer-term in vivo retention, degradation kinetics, and structural evolution over time are important and are the subject of ongoing and future investigations, but were beyond the focused objectives of this manuscript.

Response to reviewer #3

Note: when line numbers are given here by us, they refer to the new MS version with tracking.
New text is shown in orange

Reviewer #3 (Remarks to the Author):
The work is interesting and valuable, although several aspects related to the characteristics of the pectins used in the studies and the conditions of their cross-linking and gelation should be explained in more detail. The manuscript requires major revision.
We thank the reviewer for finding our work interesting and have addressed his comments as follows:
1. The different pectic materials used in the study were analyzed in detail by a new collaborator, Prof. Bunzel who is now also listed as co-author of our study. The corresponding results are summarized in the new Figure 4 in the main text, new Figures S4 and S5 as well as tables S3, S4, S5 and text on page 11 of the SI. In addition, lines 65-99 and 254-312 in the main text are now dealing with pectin chemistry and description of the analytical results.
2. Conditions of film preparation and gelation are explained more clearly in the Methods section, see also individual comments below.
L 323 Pectin must be chemically characterized – monosaccharide composition, DM, DA, /Mw. These parameters influence mainly the cross-linking processes.
Monosaccharide composition is now shown in the new Figure 4 of the main text, also in table S3 in the SI. DM and DA are shown in table S5 and MW analysis is displayed in Figure S4. The corresponding data are also inserted in the results section of the main text, lines 278-312.
L 328 How long was the dissolution of pectin carried out? It has a crucial role in the spatial arrangement of pectin molecules.
We thank the reviewer for this question. Pectin dissolution was carried out for a fixed and standardized duration to ensure complete hydration and uniform dispersion prior to film casting. Specifically, pectin was dissolved in aqueous solution under continuous stirring for 2 hours at room temperature, a time point that we have found sufficient to achieve full dissolution without inducing polymer degradation or chain scission. This dissolution time was applied consistently across all batches to minimize variability in film formation and downstream analyses.
New text, line 620: After 2 hours of continuous stirring at room temperature the dissolved pectin was poured into custom molds and cured to glass-phase films, also at room temperature.
L 330 Describe the method of pectin film preparation with details, the amount of pectin sample, the temperature of drying, the time of drying, or the drying conditions.
In line 616 we wrote: …pectin powder was dissolved to 3% (w/w) at 25°C by a step-wise addition of deionized water. For the drying temperature see comment above.
L 234-235 Pectin is a very complex family of polysaccharides; it can be divided into several main domains (homogalacturonan, rhamnogalacturonans), and their chemical composition, molecular properties, structure, and thusability to absorption depend strongly on source, extraction method, and even the physiological stage of the fruit. So, the term 'pure pectin' is highly imprecise. In the experiment, no information was provided regarding these critical factors. In my opinion, it is not possible to analyze the results without considering these facts.
We are sorry for the imprecise wording which obviously led to a misunderstanding here: We meant to point out that there is a fundamental difference between using dry pectin films (which is what we placed on the lung surface) and the wet pectin gels used as samples for EM imaging before.
We rephrased this, see in line 457:
In our surgical setting we applied pectin in the form of dry films which had been prepared without any additives and were initially only hydrated by water uptake from the organ surface.
236 Pecin gelling ability depends mainly on the number of methyl esters of carboxyl groups of galacturonic acid. The mechanism of gelling should be explained, especially given that calcium ions were applied along with a high sucrose concentration and low pH.
We are sorry for the imprecise wording which obviously led to a misunderstanding here: Our films contained **no additives** such as sucrose or Ca ions which had been used in the papers referenced (14, 15). This is now hopefully clearer in the new text, line 458:
In contrast, pectin was used in gel form in earlier attempts to visualize its ultrastructure and was stabilized for EM imaging by lowering the pH and adding high concentrations (30% - 50%) of sucrose, 100mM NaCl, or complexing ions such as Ca^{2+} in different combinations to the fixatives, depending on the composition of the pectin gels [14,15]. Usage of such additives was not advisable in our combined system of animal tissue and pectin patch for the following reasons:

Dear editor, dear Dr. Bu

please find enclosed the revised version of the manuscript COMMSBIO-25-7708-A, with the title ***Plant heteropolysaccharides and their bioadhesion to animal glycocalyx visualized at the nanoscale***, which we would like to resubmit to “Communications Biology”.

We formatted MS and SI according to the styling and formatting guide, therefore we will upload both, versions with tracking and without.

All source data and raw images are uploaded to Heidelberg University’s repository HeiData under the DOI https://doi.org/10.11588/DATA/5LW3JS and will be published once the technical screening is done.

Regarding the minor requests of reviewer #4:

Table S3 – please use the same number of significant digits.

We agree that the Table needed to be adjusted. We have reformatted the table so that the measured value and its measurement uncertainty (range/2) share the same number of decimal places.

We also adjusted in most cases the number of significant digits. However, since the individual monosaccharides in this table differ substantially in their absolute concentrations (e.g., GalA at ~55%, Xyl at ~0.6%) as well as in their analytical precision, enforcing a single, uniform number of significant digits across all analytes and samples would not accurately reflect the true measurement uncertainty of each value. In some cases, this would artificially inflate the apparent precision of low-abundance analytes; in others, it would truncate genuinely available precision for high-abundance analytes.

Nevertheless, an adjustment was absolutely necessary, and we believe that the table in its current form actually better reflects the data.

Table S5 – please add standard error values.

Done. We added the range/2 as an indicator for the measurement uncertainty.

Figure S4 – please add a discussion of the SEC analysis results; it is difficult to determine the molecular weight from the graph alone.

We added a note under the graph:

“As SEC separates molecules according to their hydrodynamic volumes, it is not possible to determine exact molecular weights unless the calibration standards (here: dextrans) show the same chemical-structural characteristics. Therefore, the chromatograms shown here can only demonstrate relative shifts in molecular weight (again based on hydrodynamic volumes) and indicate the distributions as being bi- or trimodal.“

The distribution (bi- or trimodal) and the relative ranking of the molecular weights (based on hydrodynamic volume) has already been discussed in the main manuscript. We believe that, given the methods used, specifying exact molecular weights would constitute an overinterpretation of the data.

Please check the response to the comment: “236 Pecin’s gelling ability depends mainly on the number of methyl esters of the carboxyl groups of galacturonic acid. The mechanism of gelling should be explained, especially given that calcium ions were applied along with a high

sucrose concentration and low pH." It appears the response is incomplete since it ends with a colon (:).

This must be a misunderstanding, our response in the last rebuttal letter (text in blue) indeed ended with a colon, because our new text followed. The new text (orange) also ends with a colon after "following reasons:", because in the MS we then present the reasons, which is old text. Maybe we should have added that old text (now in green below) to the rebuttal letter to make the syntax clearer.

Old rebuttal:

We are sorry for the imprecise wording which obviously led to a misunderstanding here: Our films contained **no additives** such as sucrose or Ca ions which had been used in the papers referenced (14, 15). This is now hopefully clearer in the new text, line 458:
In contrast, pectin was used in gel form in earlier attempts to visualize its ultrastructure and was stabilized for EM imaging by lowering the pH and adding high concentrations (30% - 50%) of sucrose, 100mM NaCl, or complexing ions such as Ca^{2+} in different combinations to the fixatives, depending on the composition of the pectin gels [14,15]. Usage of such additives was not advisable in our combined system of animal tissue and pectin patch for the following reasons: Low concentrations of Ca ions have been used successfully in fixatives for EM before,[22] but pH values as low as 3.0 are not desirable for animal tissue fixation since average pH values in mammalian cytoplasm are usually between 7.0 and 7.4. [22]

We hope to have now addressed all concerns and are looking forward to hearing from you.

Best regards

Irene Wacker & Rasmus Schröder
