# Supplementary material for: Plant heteropolysaccharides and their bioadhesion to animal glycocalyx visualized at the nanoscale
Source: Commun Biol. 2026 Sep 22;9:1238. doi: 10.1038/s42003-026-10872-y (PMC13597490; doi:10.1038/s42003-026-10872-y)
Supplement: Supplementary file 3 [file 42003_2026_10872_MOESM1_ESM.pdf]

Supplementary Information

Plant heteropolysaccharides and their bioadhesion to animal glycocalyx visualized at the nanoscale

Irene Wacker*, Willi Wagner, Ronald Curticean, Anna Schoch, Endre Majorovits, Jennifer M. Pan, Steven J. Mentzer, Mirko Bunzel, Rasmus R. Schröder

I. Wacker, R. Curticean, R. R. Schröder
BioQuant, Universität Heidelberg, Im Neuenheimer Feld 267, 69120 Heidelberg, Germany and 3DMM2O, Cluster of Excellence (EXC-2082/1 – 390761711)

W. Wagner
Department of Diagnostic and Interventional Radiology, University Hospital of Heidelberg, Heidelberg, Germany and Translational Lung Research Center Heidelberg (TLRC), German Center for Lung Research (DZL), University of Heidelberg, Heidelberg, Germany.

Current address: Diagnostic Radiology and Neuroradiology, Greifswald University Hospital, Ferdinand-Sauerbruch-Strasse 1, Greifswald, Germany.

A. Schoch, M. Bunzel
Department of Food Chemistry and Phytochemistry, Karlsruhe Institute of Technology (KIT), Adenauerring 20A, 76131 Karlsruhe, Germany

E. Majorovits
Carl Zeiss Microscopy GmbH, Carl-Zeiss Str. 22, D-73447 Oberkochen, Germany

J. M. Pan, S. J. Mentzer
Laboratory of Adaptive and Regenerative Biology, Brigham & Women's Hospital, Harvard Medical School, Boston, MA 02115, USA

*To whom further correspondence may be addressed:
E-mail: irene.wacker@bioquant.uni-heidelberg.de

Contents:

Stabilizing pectin **page 3**
Suppl. Table 1 Hydration factors of citrus pectin in different solutions page 3
Suppl. Table 2 Ethanol concentrations during EM preparation page 4
Suppl. Figure 1 Hydration factors for different pectic polysaccharides page 5
Suppl. Figure 2 Dehydration kinetics of lemon pectin page 6
Suppl. Figure 3 Different faces of a lemon pectin film page 7

Analysis of different pectic polysaccharides (PPS) **page 8**
Suppl. Table 3 Monosaccharide composition of PPS page 8
Suppl. Table 4 Methylation analysis data of PPS page 9
Suppl. Table 5 Degree of pectin methylation and acetylation page 10
Suppl. Figure 4 Molecular weight - size exclusion chromatograms page 10
Additional details to interpretation of PPS structural data page 11
Suppl. Figure 5 Schematic representation of the PPS analyzed page 12
Suppl. Figure 6 Ultrastructural signatures of different PPS page 13
Image analysis - assessing porosity and network characteristics page 14
Suppl. Figure 7 Extracting porosity and network characteristics page 15

Quality of tissue preservation **page 16**
Suppl. Figure 8 Lung surface/mesothelium page 16
Suppl. Figure 9 Type 2 alveolar cells page 17
Suppl. Figure 10 Membrane-bound organelles page 18

Adhesion of pectin film to lung mesothelium **page 19**
Suppl. Figure 11 Judging of trimmed blockface with light microscopy page 19
Suppl. Figure 12 Targeting for 3D analysis with FIBSEM nanotomography page 20

References **page 21**

List of Movies **page 21**
Wacker_Suppl. Movie_1 Different faces of a lemon pectin film – fly-in: overview to detail
Wacker_Suppl. Movie_2 Preservation of lung tissue - fly-in: overview to details
Wacker_Suppl. Movie_3 FIBSEM nanotomography data - fly-through
Wacker_Suppl. Movie_4a-c Annotated data – 3D visualization from different angles

Stabilizing pectin

To avoid the inevitable dissolution in aqueous media of the pure pectin films used in our study, we systematically analyzed how the different solutions utilized in a typical embedding procedure for ultrastructural analysis influenced film quality. Apart from morphological characteristics we derived a hydration factor by gravimetric analysis of small pieces of film before and after incubation with the respective solution, cf Table 1 main text.
The influence of different ethanol concentrations, alone or in combination with one or more components of the fixatives, on the hydration factors of lemon and orange pectin was also determined (Table S1)

Suppl. Table 1 – Hydration factors of lemon and orange pectin in different concentrations of ethanol, combined with components of aqueous solutions used during a typical preparation protocol for EM imaging.

Solvent	Lemon 15% EtOH	Orange 15% EtOH	Lemon 25% EtOH	Orange 25% EtOH
**EtOH**	10.8/14.1	11.8	4.0/3.4	4.2
**Pipes & EtOH**	21.5/15.4*	17.0*	6.2	12.9*
**GA & EtOH**	13.3/12.9	10.7	4.1	5.2
**GA & Pipes & EtOH**	17.0/21.2*	13.7*	6.6	8.4
**GA & Pipes & RR & EtOH**	10.0/13.0	10.2	8.6 (20%)	6.4

* These samples were no longer opaque, manageable and intact films but had disintegrated into several small, transparent pieces of material.
Hydration factor = weight(after incubation)/weight(initial) with incubation times of typically 15min - 30min.
EtOH = ethanol, concentration as given in column header
Pipes = 0.1M Pipes buffer
GA = 2.5% glutaraldehyde
RR = 0.15% Ruthenium red

Based on the finding that films with hydration factors >15 after fixation were no longer manageable, and tended to dissolve completely during the course of EM preparation we aimed for initial hydration factors around 10 for ultrastructural analyses. During the subsequent dehydration necessary for infiltration with the hydrophobic embedding resin hydration factor dropped to around 5 (Fig. S1) which then led to cross sections exhibiting clearly distinguishable filamentous networks (Fig. S2). Initial hydration factors below 5 decreased to around 2 after dehydration and resulted in very compact material without any structural detail (cf Fig. 2 main text).

Suppl. Table 2 Ethanol concentrations used during preparation protocol for electron microscopy

Our protocol for EM fixation:
30min 1st fixative, wash away fixative with buffer 3x 5', 1h 2nd fixative, wash again with buffer
Primary fixative: 2.5% glutaraldehyde (GA), 0.15% ruthenium red (RR) in 0.1M Pipes pH 6.8
Secondary fixative: 1% OsO_4, 0.15% RR in 0.1M Pipes pH 6.8
Wash solution: 0.1M Pipes pH 6.8

In addition, the following concentrations of ethanol were added:

			**[ethanol] added to**			
**sample**	HF*	shown in Fig.	primary fixative	wash	secondary fixative	wash
lemon pectin	13/15.2<br>4.7	2B/S2<br>2A	15%<br>40%	20%<br>40%	20%<br>50%	20%<br>50%
orange pectin	19.5	3A	15%	20%	20%	20%
soy pectin	5.2	3B	15%	20%	20%	20%
potato pectin**	6.5	3C	40%	40%	50%	50%
lemon pectin	15	S3A	20%	25%	25%	25%
lemon pectin + CMC	23.5	S3B	20%	25%	25%	25%
sugar beet pectin + CMC	15	S3C	20%	25%	25%	25%
CMC	30	S3D	20%	25%	25%	25%
lung tissue + pectin	n.a.	4,5, S4-S6	15%	20%	20%	20%

*HF = Hydration factor = weight (after 2nd washing period)/weight(initial)
** all incubation times were halved for this sample

Note:
For pectin on lung 1.25% GA, 2% PFA, 0.15% RR in 0.1M Pipes with 15% ethanol added were used as primary fixative. The usage of two aldehydes was warranted here because PFA (paraformaldehyde) penetrates tissue faster than GA, but PFA alone lacks cross-linking capabilities and thus does not achieve good ultrastructural preservation.

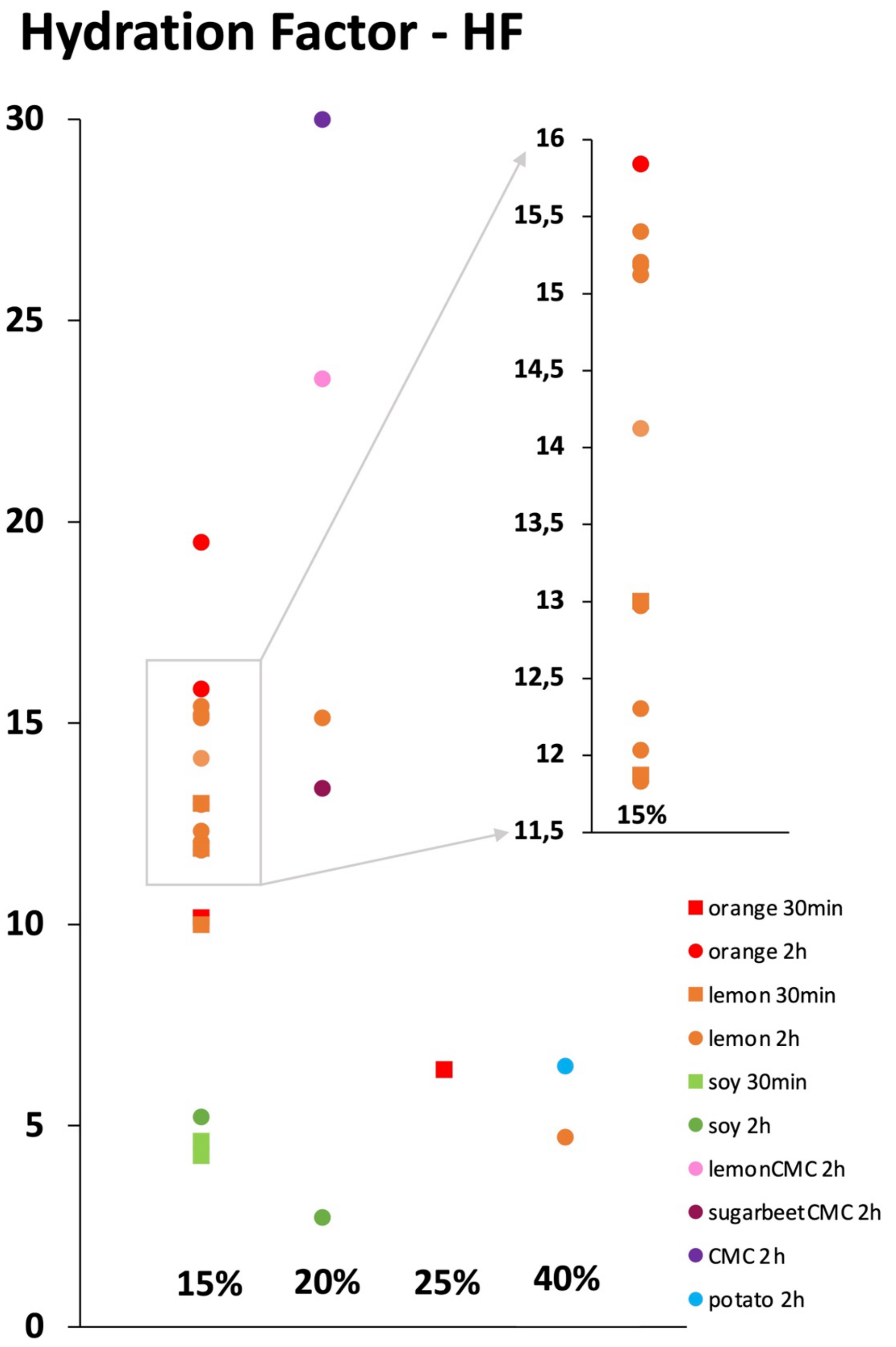

Suppl. Fig. 1 Hydration factors of different pectic polysaccharides
Plotted are the hydration factors (weight after hydration/dry weight) achieved in separate experiments depending on the ethanol concentration added. Incubation times were either 30 min in primary fixative (squares) or 2h in primary, then secondary fixative, with washing steps included (circles). Lemon was always used as a reference. Maximal hydration for lemon in 15% ethanol was already reached after 30 min since the average values (mean ± SD) for 30 min incubation (11.62± 1.52, n=3) were not significantly different from those of the longer incubation (13.63±1.52, n=7) within the combined 1σ level.

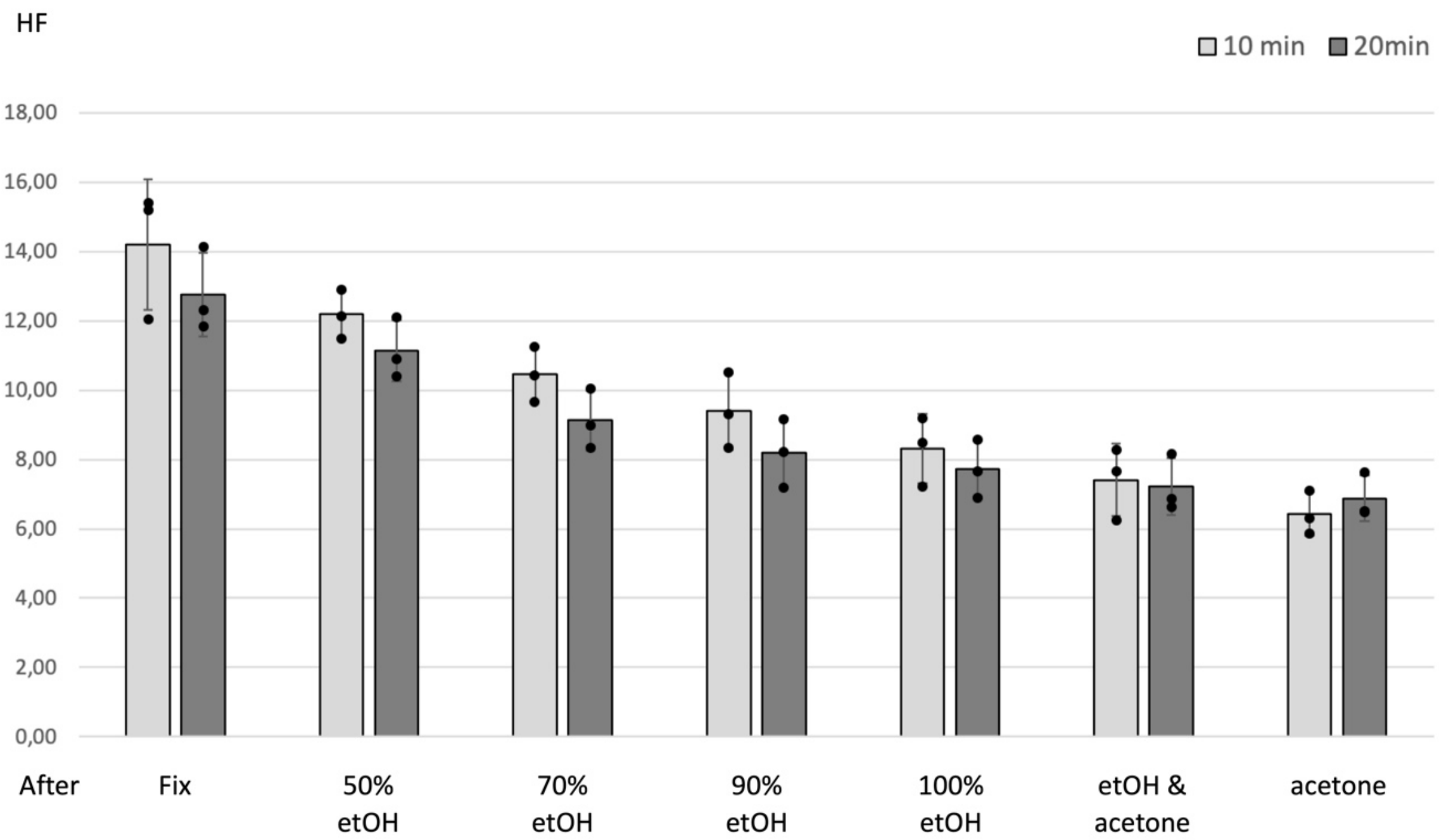

Suppl. Fig. 2 Dehydration kinetics of lemon pectin films with 10 min or 20 min incubation time during dehydration steps. For each experiment three pieces of film were weighed initially as dry material. They were then incubated for 30 min with the primary fixative (2.5% GA, 0.15% Ruthenium Red, 15% ethanol in 0.1M Pipes), washed 3x5 min with 20% ethanol in 0.1M Pipes, incubated 1h in a mimic of the secondary fixative (0.15% ruthenium red, 20% ethanol in 0.1M Pipes), washed again 3x5 min with 20% ethanol in 0.1M Pipes, and weighed a second time ("Fix"). After that they were dehydrated in a graded ethanol series followed by acetone with 10 min or 20 min incubation time for each step. During that interval they were weighed after each dehydration step. All values were normalized to dry weight (= hydration factor, HF) and are plotted as average ± SD with the original data points inserted as dots.

The statistical analysis shows, that the individual pairs (10/20 min experimental method) overlap within a 1σ combined uncertainty, thus would not indicate a significant difference. However, the 10min experiment values are consistently higher than the 20min values (6 out of 7 data points) with an average difference of +0.76 (not excluding the one outlier) with a standard deviation of the difference of 0.72. The offset is therefore +0.76 +/- 0.72, which includes zero only at the 2σ level, i.e. the 10min experiments show a systematic higher value.

Note: In that experiment, aimed to analyse the dehydration behavior of a film after limited hydration during fixation, OsO_4 was not added to the second fixative since no further embedding was planned and OsO_4 is highly toxic. It was shown to have only very minor effects on the osmolarity of fixatives[1], in fact, aqueous solutions of OsO_4 are hypotonic. [22]

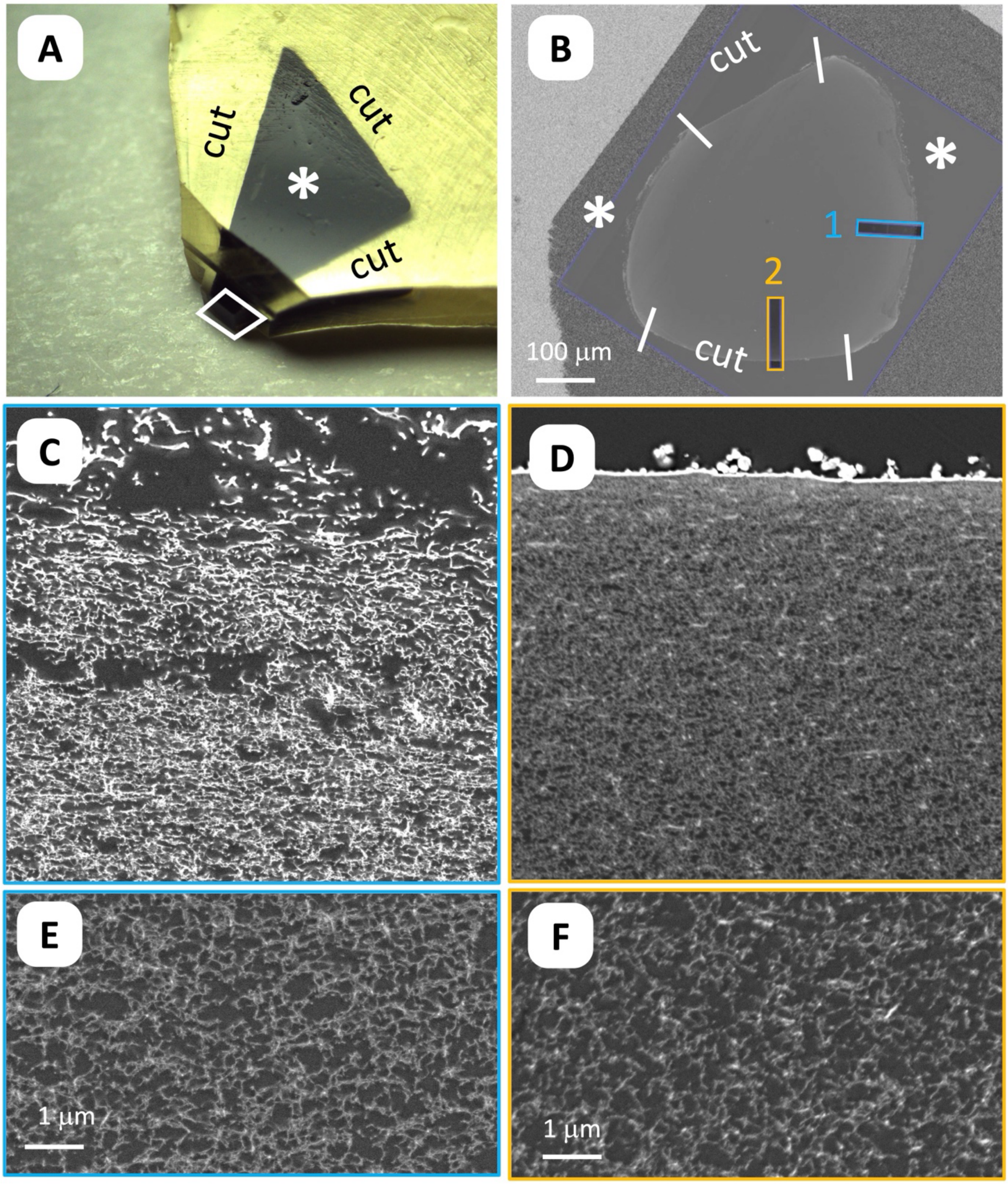

Suppl. Fig. 3 The different faces of a lemon pectin film: (A) Small piece cut from a larger film, hydrated in the presence of our canonical fixatives with 15%/20% ethanol added (cf table 2), embedded in epoxide resin, and trimmed for ultrathin sectioning (blockface indicated by white parallelogram). Stars indicate the original surface (interface of pectin to air or dish). Overview (B) of cross section with different faces separated by white lines (c.f. brackets fig. 2 Bi). Radial profiles with high spatial resolution were recorded at the positions indicated. Profile 1 extends from the original surface of the film (C) towards the centre (E) of the embedded sample, profile 2 from a cut edge (D) towards the center (F). C-F are screenshots from different positions within the radial profile, see also Suppl. movie_2.

Analysis of different pectic polysaccharides (PPS)

Suppl. Table 3 Monosaccharide composition of the applied PPS as determined after methanolysis and hydrolysis using trifluoroacetic acid (mol% ± range/2, n=2)

Ara, arabinose; Gal, galactose; GalA, galacturonic acid; Glc, glucose; GlcA, glucuronic acid; Man, mannose; Rha, rhamnose; Xyl, xylose; n.d., not detected; tr., trace amounts

	Fuc	Rha	Ara	Gal	Glc	Xyl	Man	GalA	GlcA
**Lemon pectin L1**	tr.	7.1 ± 0.2	7.6 ± 0.3	27.0 ± 0.3	1.94 ± 0.05	0.63 ± 0.1	0.58 ± 0.04	55.1 ± 0.3	tr.
**Lemon pectin L2**	tr.	7.2 ± 0.4	7.9 ± 0.6	27.6 ± 2.6	1.7 ± 0.2	0.60 ± 0.03	0.50 ± 0.002	54.5 ± 3.5	tr.
**Orange pectin**	tr.	7.4 ± 0.1	13.6 ± 0.2	19.4 ± 0.1	4.04 ± 0.03	0.90 ± 0.01	0.57 ± 0.01	54.1 ± 0.4	tr.
**Soy pectin**	10.0 ± 0.1	12.9 ± 0.1	4.78 ± 0.03	13.4 ± 0.1	0.236 ± 0.001	25.1 ± 0.1	n.d.	33.0 ± 0.2	0.53 ± 0.01
**Potato pectin**	n.d.	6.5 ± 0.1	14.3 ± 0.1	71.0 ± 0.1	0.273 ± 0.001	0.48 ± 0.01	tr.	7.3 ± 0.1	0.15*
**Sugar beet pectin**	n.d.	4.29 ± 0.03	64.2 ± 0.9	12.1 ± 0.2	11.8 ± 1.1	2.18 ± 0.04	0,99 ± 0.01	3.07 ± 0.02	1.35 ± 0.04

*based on one value

Suppl. Table 4 Methylation analysis data of the applied PPS (mol% ± range/2, n=2)

Ara, arabinose; Gal, galactose; Glc, glucose; Man, mannose; Rha, rhamnose; Xyl, xylose; n.d., not detected; tr., trace amounts; PMAA, partially methylated alditol acetates; *f*, -furanose; *p*, -pyranose

PMAA	Lemon pectin L1	Lemon pectin L2	Orange pectin	Soy pectin	Potato pectin	Sugar beet pectin
t-Ara*f*	12.1 ± 0.5	12.8 ± 0.8	20.7 ± 0.7	4.1 ± 0.3	2.8 ± 0.05	29.5 ± 1.6
t-Ara*p*	1.2 ± 0.3	0.7 ± 0.04	1.1 ± 0.2	0.2 ± 0.01	1.1 ± 0.1	n.d.
1,2-Ara*f*	1.1 ± 0.1	1.1 ± 0.1	1.1 ± 0.2	1.1 ± 0.2	0.2 ± 0.01	0.3 ± 0.1
1,5-Ara*f*	8.9 ± 0.6	8.4 ± 0.9	13.9 ± 0.3	5.3 ± 0.4	30.3 ± 0.6	25.0 ± 0.2
1,2,5-Ara*f*	1.5 ± 0.004	1.6 ± 0.1	2.0 ± 0.1	tr.	tr.	1.6 ± 0.04
1,3,5-Ara*f*	1.1 ± 0.001	1.9 ± 0.8	4.0 ± 0.2	n.d.	tr.	20.3 ± 1.8
1,2,3,5-Ara*f*	1.5 ± 0.2	2.8 ± 0.9	2.7 ± 0.2	n.d.	tr.	4.6 ± 0.3
**Sum Ara**	**27.4 ± 0.5**	**29.2 ± 3.3**	**45.5 ± 0.7**	**10.7 ± 0.8**	**34.3 ± 0.6**	**81.3 ± 3.9**
t-Gal*p*	9.9 ± 0.2	9.4 ± 0.1	6.2 ± 0.01	25.2 ± 0.5	6.8 ± 0.1	3.0 ± 0.8
1,3 Gal*p*	tr.	tr.	tr.	tr.	1.1 ± 0.02	1.1 ± 0.1
1,4-Gal*p*	34.6 ± 1.4	33.8 ± 0.4	21.0 ± 0.2	3.6 ± 0.7	49.4 ± 0.5	4.9 ± 2.2
1,6-Gal*p*	3.8 ± 0.1	3.2 ± 0.3	2.3 ± 0.2	tr.	tr.	1.5 ± 0.02
1,3,6 Gal*p*	2.5 ± 0.2	2.2 ± 0.1	1.6 ± 0.1	1.4 ± 0.1	0.4 ± 0.01	0.9 ± 0.03
1,4,6-Gal*p*	1.9 ± 0.1	1.5 ± 0.05	1.2 ± 0.1	n.d.	1.2 ± 0.001	n.d.
**Sum Gal**	**52.6 ± 1.2**	**50.1 ± 0.7**	**32.2 ± 0.1**	**30.2 ± 1.1**	**59.0 ± 0.6**	**11.3 ± 3.0**
t-Glc*p*	1.2 ± 0.2	2.5 ± 0.5	1.5 ± 0.3	4.9 ± 1.0	0.8 ± 0.2	1.4 ± 0.4
1,4-Glc*p*	5.5 ± 0.2	6.8 ± 1.4	10.3 ± 0.1	1.9 ± 0.3	0.6 ± 0.1	3.4 ± 0.001
1,4,6-Glc*p*	0.9 ± 0.1	0.9 ± 0.3	1.1 ± 0.05	n.d.	0.1 ± 0.01	n.d.
**Sum Glc**	**7.6 ± 0.1**	**10.3 ± 2.2**	**12.9 ± 0.1**	**6.8 ± 1.3**	**1.5 ± 0.1**	**4.8 ± 0.4**
1,2 Rha*p*	6.5 ± 0.5	5.2 ± 0.1	4.5 ± 0.7	8.0 ± 0.3	1.3 ± 0.02	1.0 ± 0.3
1,2,4-Rha*p*	1.8 ± 0.1	1.8 ± 0.1	1.4 ± 0.1	6.7 ± 0.3	2.9 ± 0.1	1.6 ± 0.2
**Sum Rha**	**8.3 ± 0.7**	**7.0 ± 0.02**	**5.9 ± 0.7**	**14.6 ± 0.6**	**4.2 ± 0.1**	**2.6 ± 0.5**
t-Xyl*p*	1.6 ± 0.004	1.2 ± 0.2	2.0 ± 0.2	7.7 ± 1.2	0.9 ± 0.01	tr.
1,2-/1,4-Xyl*p**	0.4 ± 0.01	0.4 ± 0.1	0.4 ± 0.01	14.5 ± 1.9	0.2 ± 0.004	n.d.
**Sum Xyl**	**2.0 ± 0.01**	**1.6 ± 0.2**	**2.3 ± 0.2**	**22.2 ± 3.0**	**1.1 ± 0.01**	
1,4-Man*p*	1.1 ± 0.1	1.0 ± 0.1	1.0 ± 0.02	tr.	tr.	n.d.
t-Fuc*p*	0.9 ± 0.002	0.7 ± 0.1	0.3 ± 0.03	15.5 ± 0.8	n.d.	n.d.

* coeluting PMAAs, but identified based on their mass spectra

Suppl. Table 5 Degree of methylation (DM) and acetylation (DA) of pectic galacturonic acid units (in % ± range/2, n=2)

	DA	DM
**Lemon pectin L1**	0.91 ± 0.02	51.3 ± 1.3
**Lemon pectin L2**	0.66 ± 0.01	40.1 ± 1.5
**Orange pectin**	1.39 ± 0.02	40.4 ± 1.1
**Soy pectin**	0.3 ± 0.06	0.5 ± 0.07
**Potato pectin**	2.45 ± 0.05	0.0
**Sugar beet pectin**	67.3 ± 2.8	4.3 ± 0.26

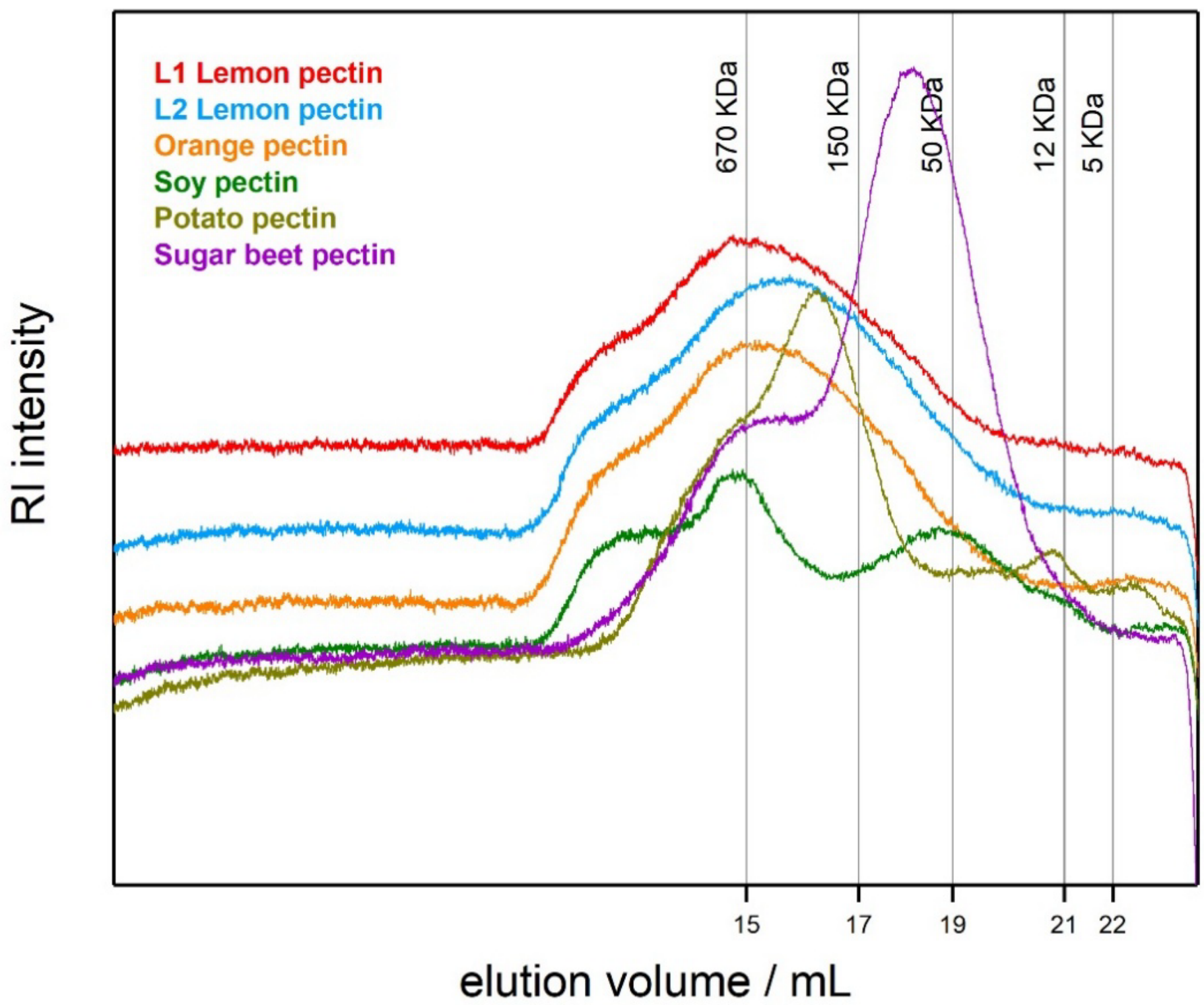

Suppl. Fig. 4 Size exclusion chromatograms monitored with refractive index (RI) detection. Molecular weights indicated are based on a calibration with dextran standard compounds.

Note: As SEC separates molecules according to their hydrodynamic volumes, it is not possible to determine exact molecular weights unless the calibration standards (here: dextrans) show the same chemical-structural characteristics. Therefore, the chromatograms shown here can only demonstrate relative shifts in molecular weight (again based on hydrodynamic volumes) and indicate the distributions as being bi- or trimodal.

Additional details to interpretation of PPS structural data from chemical analysis

A) Citrus PPS (commercial extracts from orange and lemon)

Galacturonic acid has its origin from both HG and the RGI backbone, the latter one being built of alternating galacturonic acid and rhamnose units. In both PPS, galactose, representing (arabino)galactans, dominates over arabinose, representing arabinans, in the monosaccharide composition data. This suggests acid extraction conditions as arabinans usually dominate over (arabino)galactans in (native) citrus pectin [2] but arabinans are less stable under acidic conditions. Comparing lemon and orange pectins, the dominance of (arabino)galactans is less distinct in orange pectin as compared to citrus pectin. Methylation analysis data suggest a comparable degree of RGI ramification for lemon and orange pectins (1,2,4-linked rhamnopyranose to 1,2-linked rhamnopyranose ratio of 0.28 (lemon L1), 0.35 (lemon L2), and 0.31 (orange)). Arabinans from both lemon and orange pectins are moderately branched, both in position O2 and/or O3; however, the partially methylated alditol acetate of 1,2,3,5-linked arabinofuranose may also (at least partially) be the result of undermethylation.

B) Soy PPS

Terminal xylopyranose as expected in XGA structures was identified by methylation analysis. According to methylation analysis data (Table S4), however, a larger portion of the xylose is 1,2- or 1,4-linked. This may suggest impurities such as xyloglucans (1,2-linked xylopyranose units) or secondary cell wall xylans (1,4-linked xylopyranose units); however, these units may also originate from the distinct xylogalacturonan structures that are found in soybeans. Following partial hydrolysis of soy polysaccharides, Aspinall et al. identified a 1,2-linked disaccharide composed of fucose and xylose suggesting 1,2-linked xylopyranose units as structural units soy xylogalacturonans[3]. In addition, Nakamura and co-workers identified oligomeric side-chains of 1,4-linked xylopyranose units as xylogalacturonan structural entities[4]. Arabinan side-chains appear to be a minor structural feature of the PPS (ca. 5% arabinose) as compared to (arabino)galactans (ca. 13% galactose). The arabinans are mostly unbranched as methylation analysis only revealed 1,5-linked and terminal arabinose units.

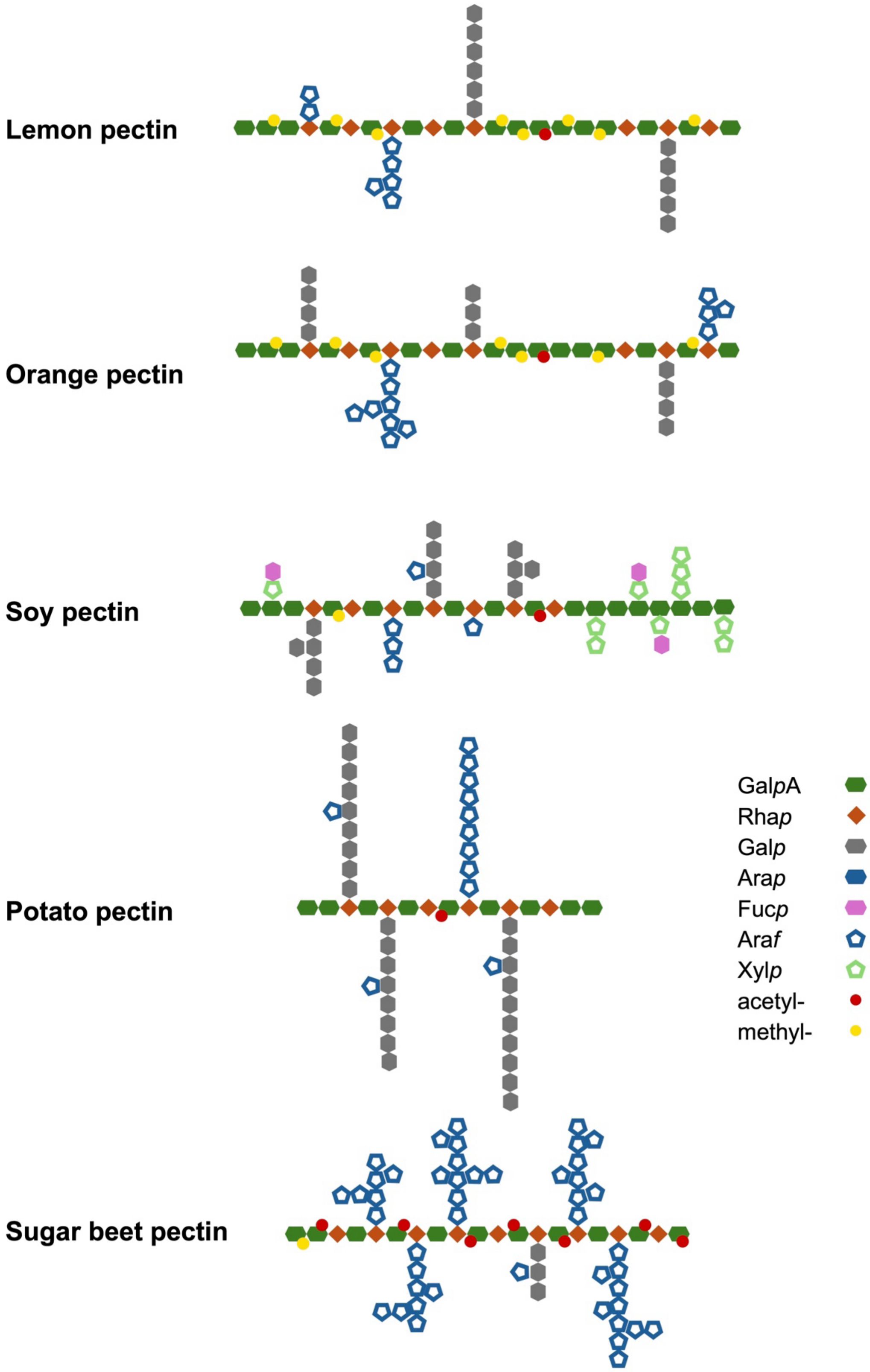

Suppl. Fig. 5 Structural representation of the different types of pectic polysaccharides analyzed

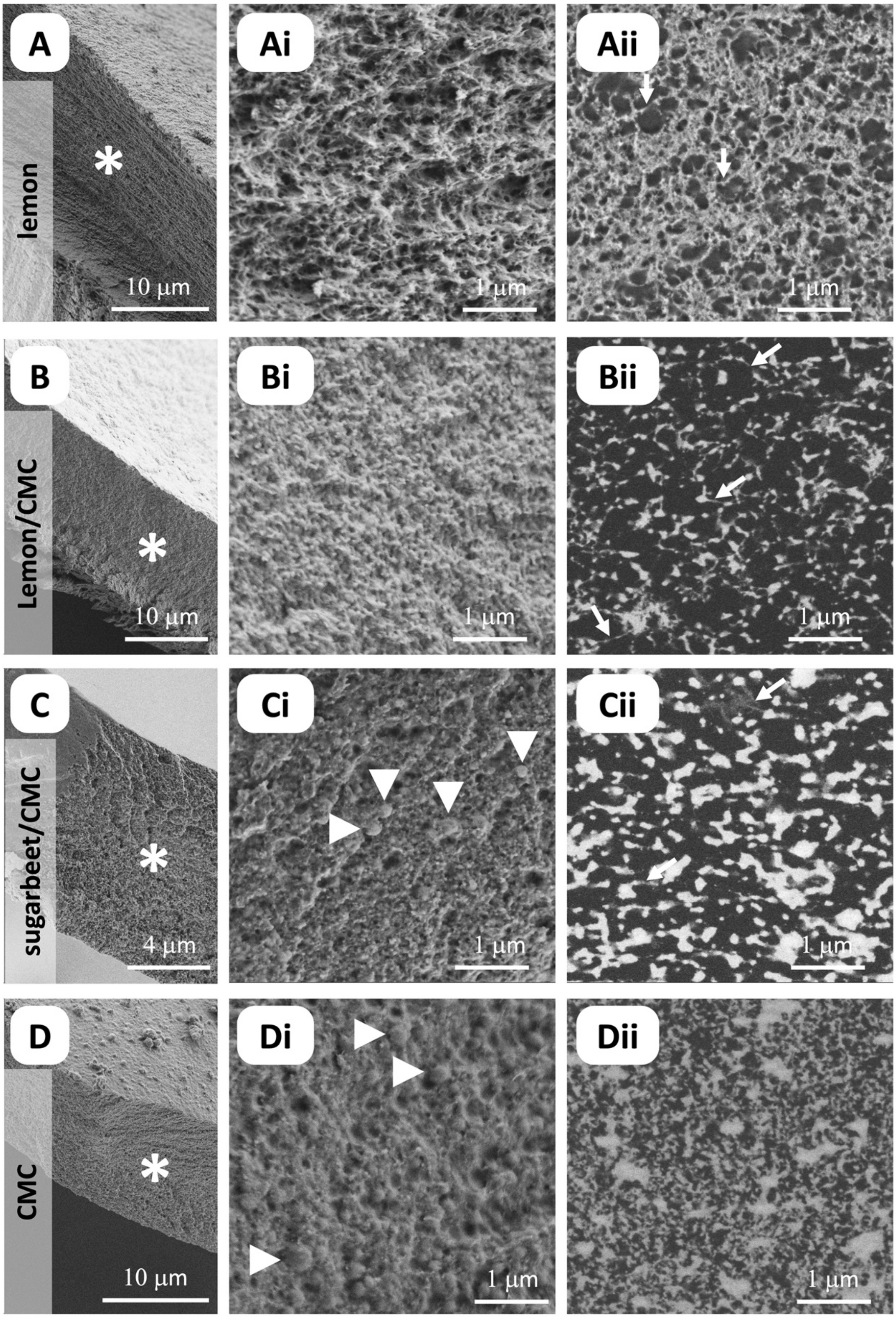

Suppl. Fig. 6 Ultrastructural signatures of different polysaccharides: (A) lemon pectin, (B) lemon pectin + CMC (carboxymethylcellulose), (C) sugar beet pectin + CMC, (D) CMC. All samples prepared with ethanol in fixative (20%) and all subsequent solutions (25%). SEM overviews (A, B, C, D) and details (Ai, Bi, Ci, Di) of fracture faces (asteriscs) from air-dried films stained with ruthenium red and OsO_4 recorded using secondary electrons. Cross sections from films embedded in epoxide after staining as above (Aii, Bii, Cii, Dii) imaged with backscattered electrons, also in a SEM. Arrowheads point to globular components, arrows to filamentous structures.

Assessing porosity and network characteristics of different pectic polysaccharides by Power Spectral Densities (PSD) and fractal dimension as quantitative descriptors

As the selected areas in Suppl. Fig. 7 indicate, a naïve comparison of material's porosity, network dimensions, or the general density of its packing cannot be evaluated and compared quantitatively by commonly used measures such as e.g. pore width in an open network – as PPS derived from soy and potato simply do not show such a network. On the other hand, orange-based PPS does not show smooth and densely packed material under the preparation conditions used. Therefore, a simple descriptive comparison fails.
To apply an objective descriptor for these structures, we adapted here the Power Spectral Density (PSD) [5,6] as a possible measure of image appearance. Assessing the log-log plots of the PSD is routinely done for the quantification of surface characteristics in AFM imaging, where either smooth or rough surfaces – with almost no edge structures or many, respectively – show a distinct dependance of the PSD on spatial frequency[7,8].
It is interesting to see, that the PSDs of the three examples, and in particular the log-log plot of the PSDs, describe the images now in such a way, that common features (such as "smooth/homogeneous areas" vs. "edges" and their quantitative content in the images) can directly be deduced.
From the comparison of the log-log plots of the PSDs (Suppl. Fig. 7) we infer the following correlations:
a) Smooth areas are indicated by the flat plateaus at low spatial frequencies (indicated by the linear dependencies in orange and green). However, in the case of orange-derived pectic polysaccharide this reflects the pores (dark resin) in between the light pectic fibrous material, while in the case of soy and potato the material itself forms these light smooth areas in the images.
b) The size distribution of the smooth areas is different for the three materials, i.e. the linear plateau falls off at different spatial frequencies (red arrows).
c) The slope of the plateaus is different, which indicates a different substructure (fractal dimension). The higher slope of the potato PSD corresponds to a slightly higher fractal dimension and thus to a smoother area, i.e. larger areas of homogeneous material packing.
In our analysis these PSD characteristics were consistent for a wide range of selected areas, in Figure S7 the error bars (standard deviation) of the average of n=3 PSDs is shown.
To conclude, PSDs can be used as quantitative measure and indicate the differences and similarities of the formed pectic materials.

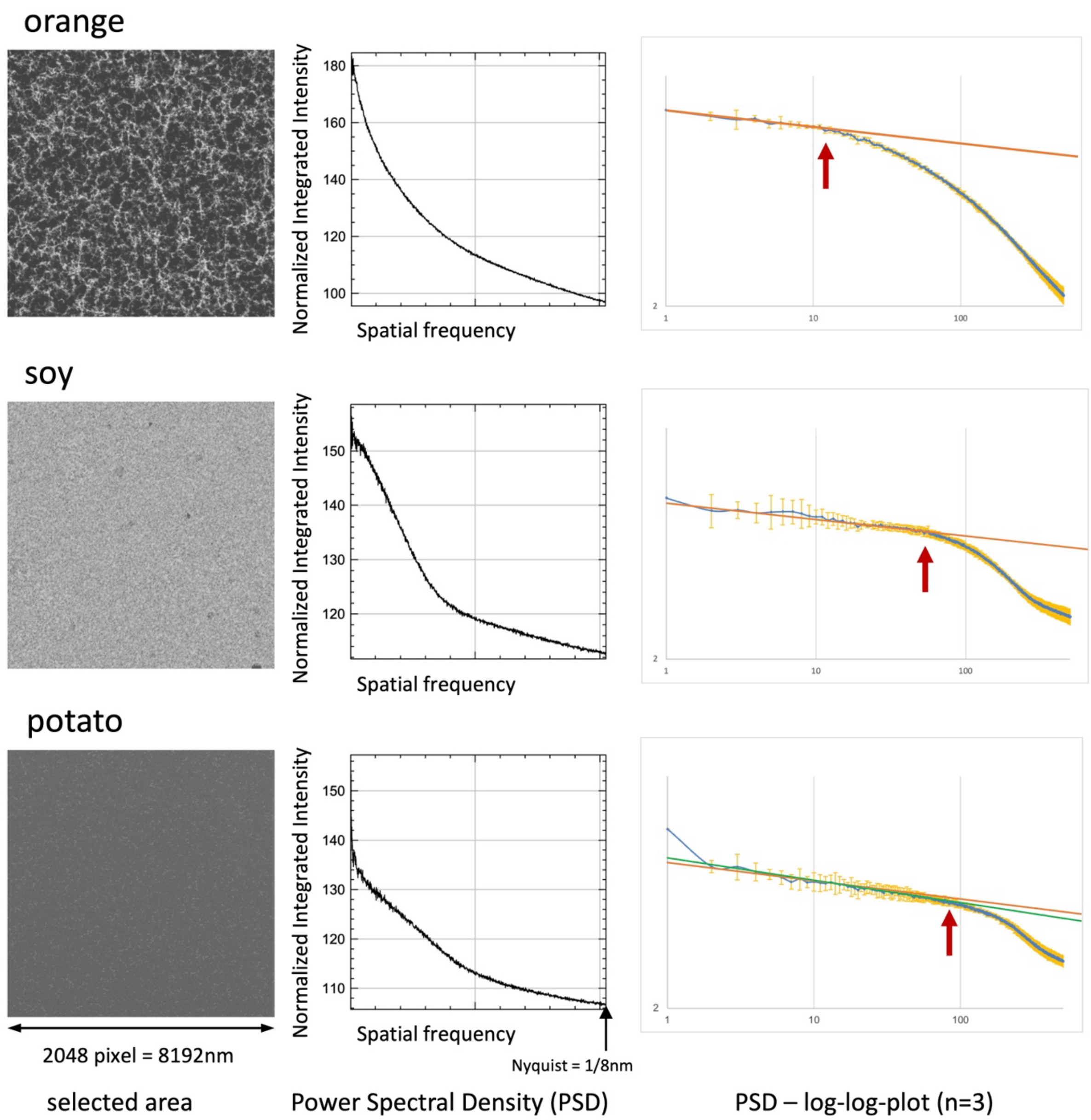

Suppl. Fig. 7 Image analysis – extracting porosity and network characteristics by PSD and fractal dimension. Three typical examples for the visualization of different PPS as discussed in Fig. 5 of the main publication text. Representative areas selected from larger images of orange, soy, and potato PPS indicate different structures already by visual inspection. PSDs (here the 1D radial average of the Power Spectrum of Fourier-transformed images) and also the log-log plots of PSDs reflect this in a quantitative manner, cf. text here in SI, page 14.

Quality of tissue preservation – SEM images of ultrathin sections

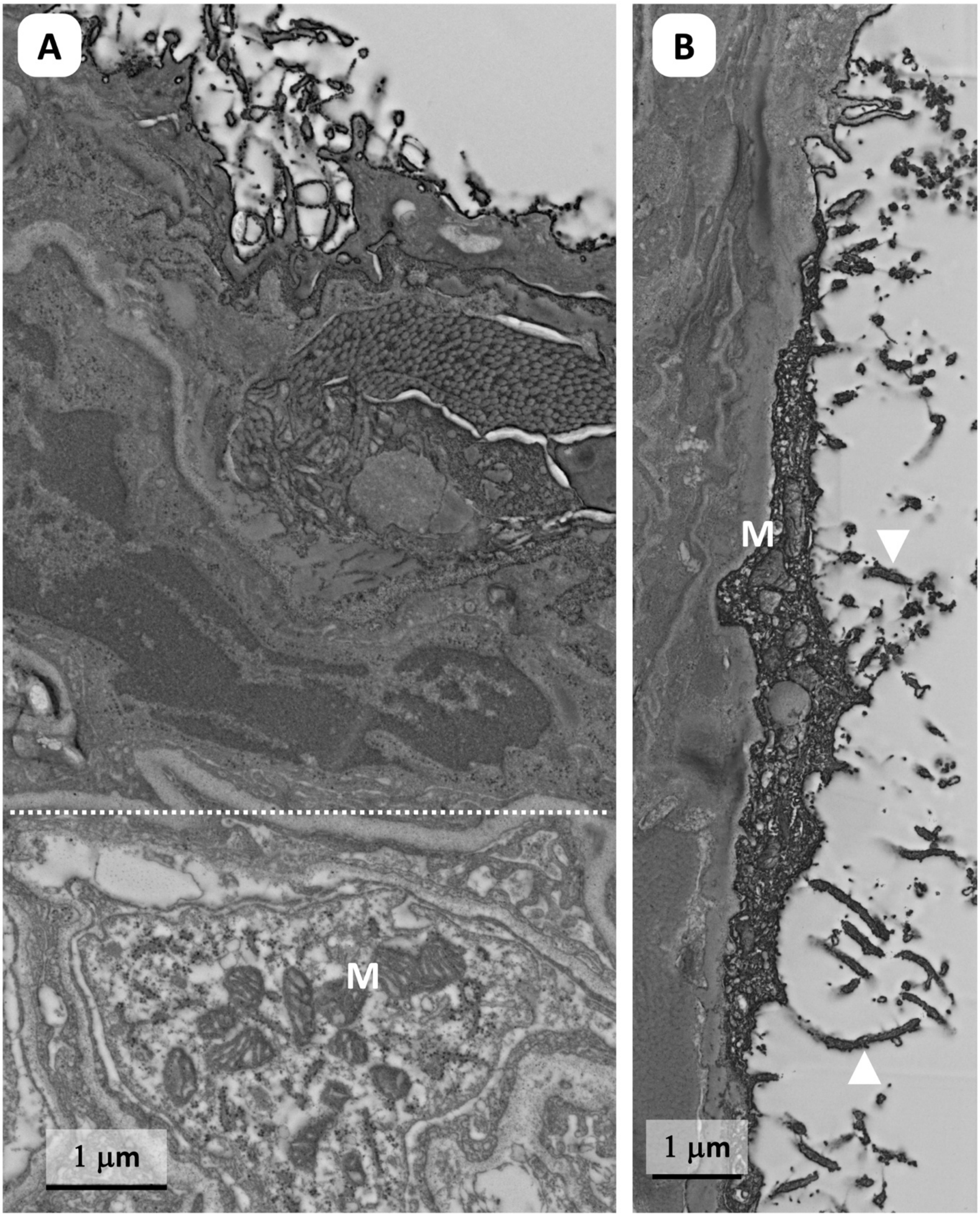

Suppl. Fig. 8 Quality of tissue prepared with 15% ethanol in primary fixative: Lung surface (A) with strongly stained mesothelial layer and submesothelial tissue, presumably caused by ruthenium red in the fixatives. In deeper tissue layers (below dashed line) the cytoplasm is less densely stained and mitochondria (M) appear dark. (B) mesothelial cell with microvilli (arrowheads) and dark, granular cytoplasm containing mitochondria which appear less dense than their surroundings. Note also fine fibrillar strands of the glycocalyx in both.

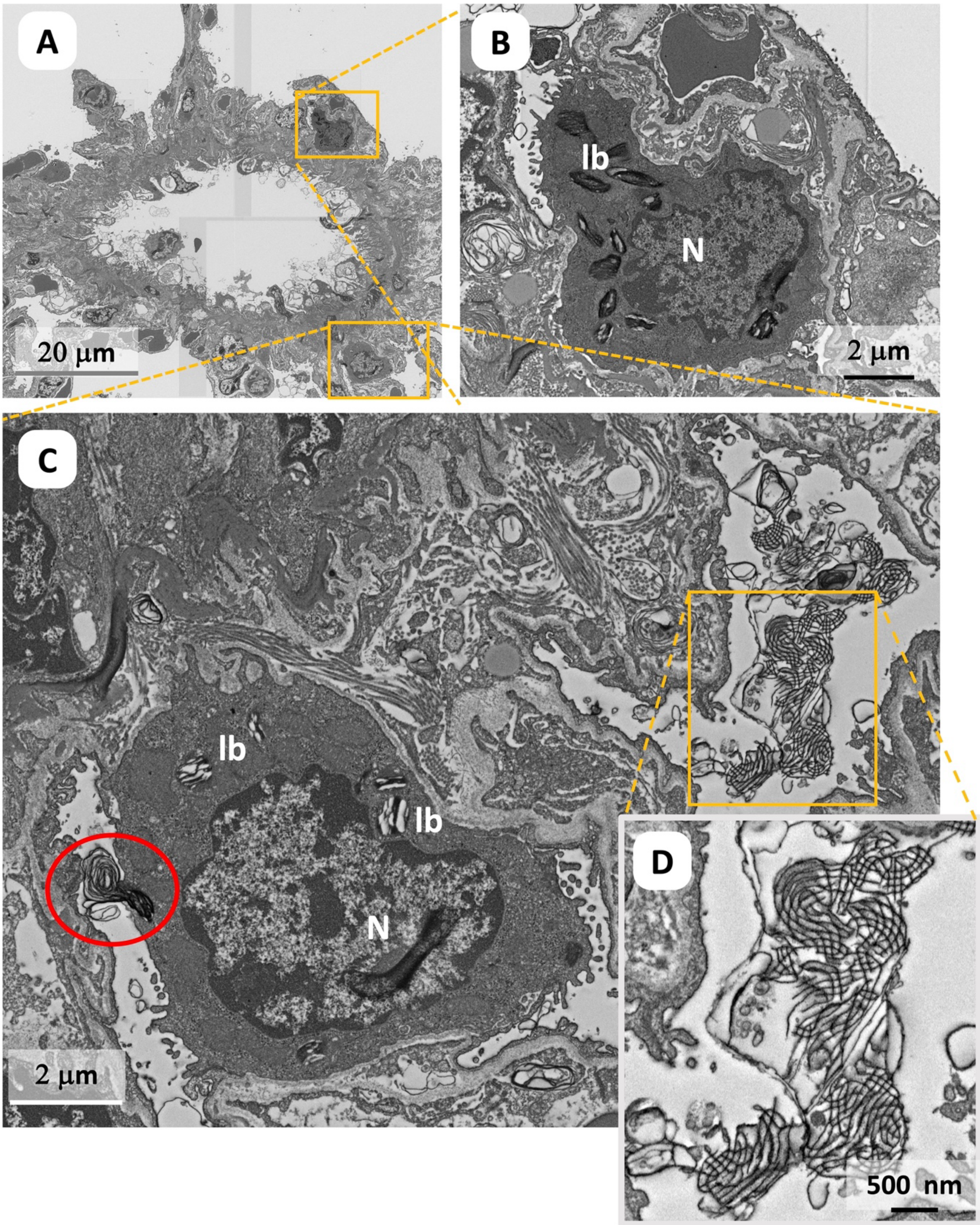

Suppl. Fig. 9 Quality of tissue prepared with 15% ethanol in primary fixative: Type 2 alveolar cells. (A) Overview of a broncheolus with surrounding alveoli with alveolar epithelial cells type 2 (B, C) with lamellar bodies (lb). Cell in (C) is secreting surfactant (red circle in C), (D) extracellular surfactant (tubular myelin). N = nucleus

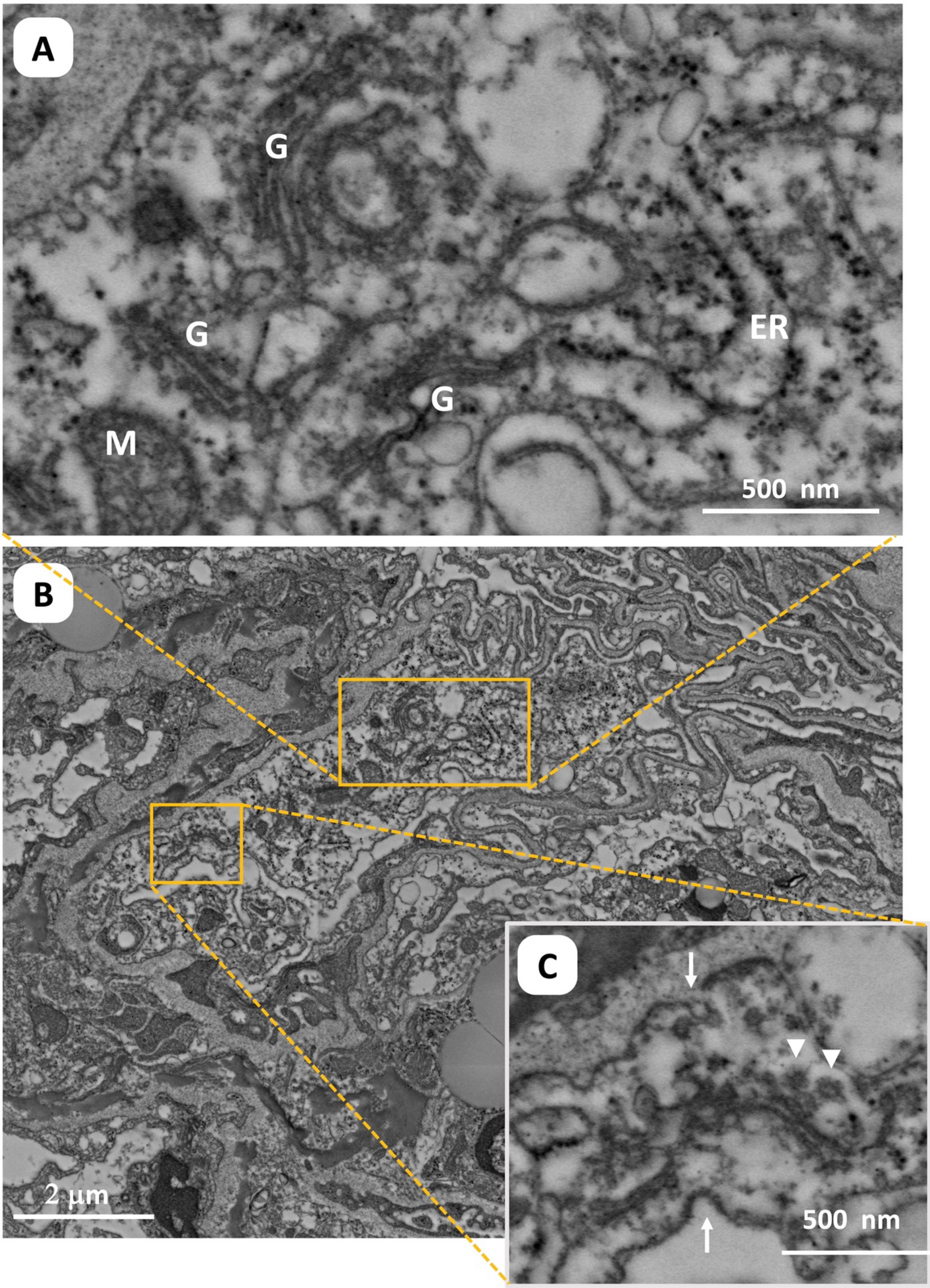

Suppl. Fig. 10 Quality of tissue prepared with 15% ethanol in primary fixative: Membrane-bound organelles (A) such as Golgi apparatus (G), endoplasmic reticulum with ribosomes (ER) in the cell shown in (B). (C) Clathrin-coated pits (arrows) and vesicles (arrowheads) are also visible. M = mitochondrium

Adhesion of pectin film to lung mesothelium

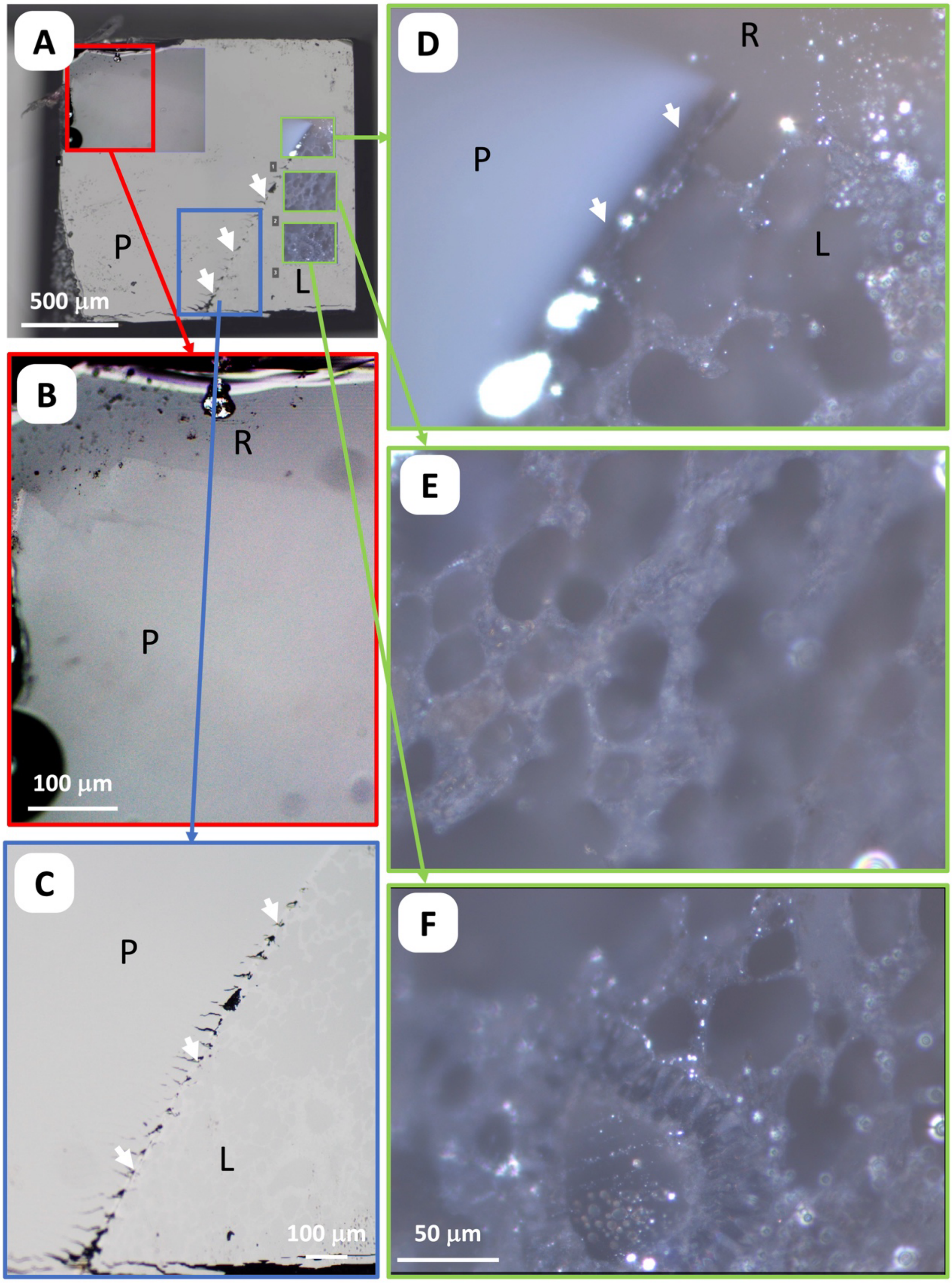

Suppl. Fig. 11 Assessment of trimmed blockface with epi-illumination light microscopy. (A) position on the surface of the trimmed block (blockface) of the different ROIs shown: (B) corner of pectin patch, P = pectin, R = embedding resin. (C) interface (arrowheads) between pectin and lung tissue (L), (A-C) visualized in brightfield mode. Darkfield mode (D-F) allows a much clearer visualization of the pectin-lung interface (D) with pectin appearing as a milky material and the lung tissue with much more structural detail (see also E, F), visible in the resin block without further staining.

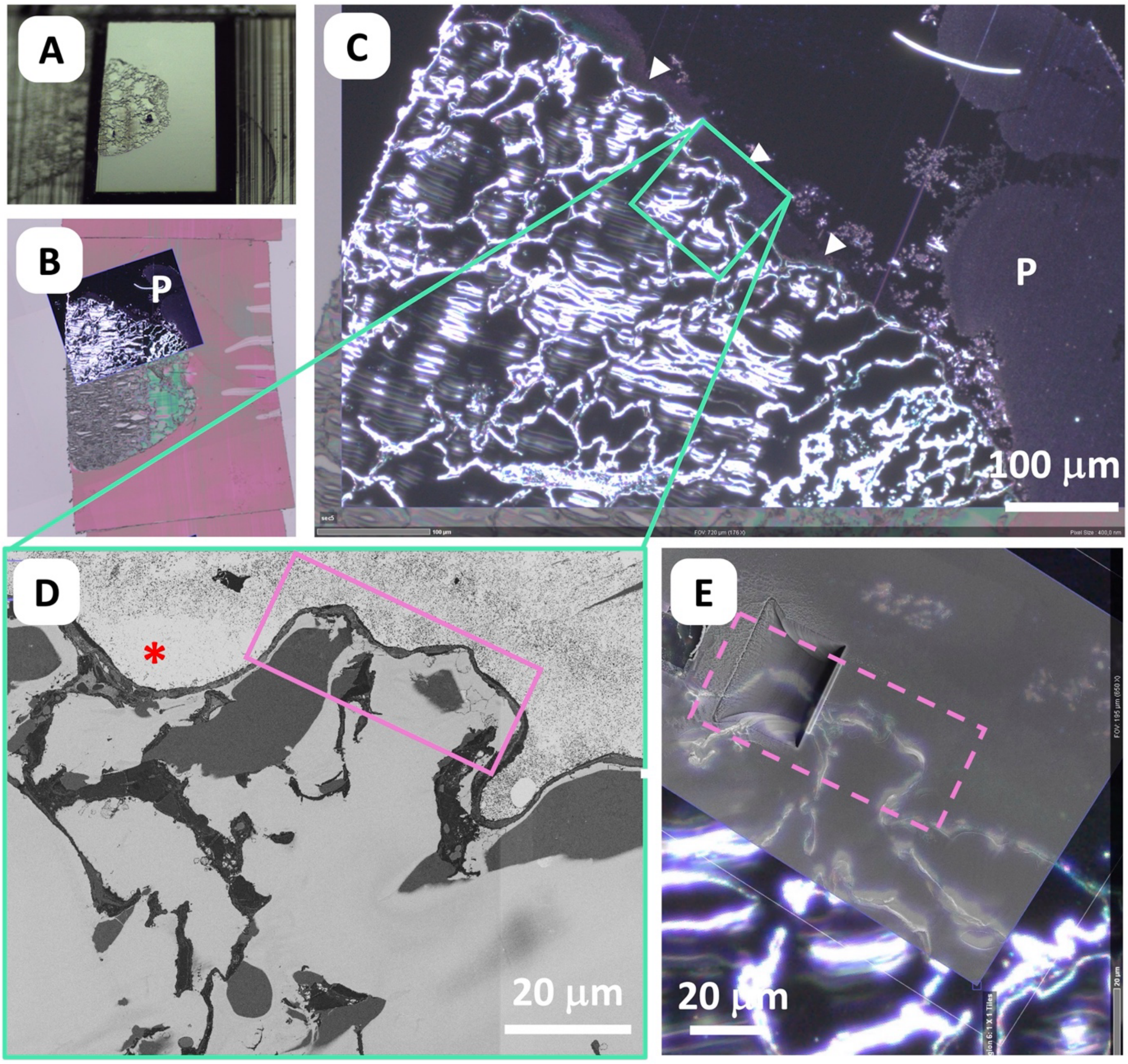

Suppl. Fig. 12 Targeting for 3D analysis with FIBSEM nanotomography: A common coordinate system established by the software packages ZENconnect, Atlas AT and Atlas 3D was used for defining and checking ROIs and for setting up the FIBSEM runs.
(A) Blockface with lung tissue (B) 500nm thick section with overlay of darkfield image showing large pectin patch (P) which is partially delaminated from the tissue surface. (C) At higher magnification pectin - appearing as milky material – is also detected close to the lung surface (arrowheads) in certain areas. (D) 80nm thick section imaged with SEM to confirm suitable ROIs with pectin really attached to the lung surface (area devoid of pectin indicated by red star). The ROI marked by the pink rectangle was identified as target ROI for the 3D analysis. (E) shows the FIB-milling trench superimposed on the corresponding area of the darkfield image.

Supplementary References

[1] Penttila, A., Kalimo H. & Trump, B. F. Influence of glutaraldehyde and/or osmium tetroxide on cell volume, ion content, mechanical stability, and membrane permeability of Ehrlich ascites tumor cells. J. Cell Biol. **63**, 197-214 (1974).

[2] Bender, C. (2023). Untersuchung der intestinalen Fermentationskinetik strukturell unterschiedlicher Pektine unter in vitro und in vivo Bedingungen. *Department of Food Chemistry and Phytochemistry* (PhD Thesis): Karlsruhe Institute of Technology (KIT).

[3] Aspinall, G., Cottrell, I., Egan, S. V., Morrison, I., & Whyte, J. Polysaccharides of soy-beans. Part IV. Partial hydrolysis of the acidic polysaccharide complex from cotyledon meal. *J. Chem. Soc. C: Org.* **11**, 1071-1080 (1967).

[4] Nakamura, A., Furuta, H., Maeda, H., Takao, T., & Nagamatsu, Y. Analysis of the molecular construction of xylogalacturonan isolated from soluble soybean polysaccharides. *Bioscience, biotechnology, and biochemistry,* ***6***, 1155-1158 (2002).

[5] Definition of Power Spectral Density in textbook: P. Stoica & R. Moses (2005). Spectral Analysis of Signals. https://user.it.uu.se/~ps/SAS-new.pdf
last access 2026-05-19

[6] Schaaf, A. van der & Hateren, J.H. van (1996) Modelling the Power Spectra of Natural Images: Statistics and Information. Vision Res. 36, 2759-2770

[7] Matlab: https://de.mathworks.com/matlabcentral/fileexchange/54297-radially-averaged-surface-roughness-topography-power-spectrum-psd?s_tid=FX_rc3_behav
last access 2026-05-19

[8] Pahwa, N. & Singh, A.R. (2025) Structural, optical and AFM based PSD surface roughness studies of cadmium telluride thin films of nanometer thickness range. Results Surf. Interfaces 21, 100655

List of movies

Wacker_Suppl. Movie_1 Different faces of a lemon pectin film – fly-in: Overview to details for two regions of interest in one section. One region extends from the original material surface towards the center, the other from a cut face of the pectin film towards the center (cf Suppl. Fig. 3)

Wacker_Suppl. Movie_2 Preservation of lung tissue – fly-in: Overview to details for three regions of interest, located at different positions within the section (cf figure S8, S9, S10).

Wacker_Suppl. Movie_3 FIBSEM nanotomography data – data stack

Wacker_Suppl. Movie_4 a-c Annotated FIBSEM data – 3D visualization from different angles
