# Supplementary material for: Plant heteropolysaccharides and their bioadhesion to animal glycocalyx visualized at the nanoscale
Source: Commun Biol. 2026 Sep 22;9:1238. doi: 10.1038/s42003-026-10872-y (PMC13597490; doi:10.1038/s42003-026-10872-y)
Supplement: Supplementary file 4 [file 42003_2026_10872_MOESM2_ESM.pdf]

Description of Additional Supplementary Files

File name: Supplementary Movie 1

Description: Cross-section of resin-embedded lemon-derived pectin prepared with 15% ethanol in fixatives: fly-in from overview to highest resolution

File name: Supplementary Movie 2

Description: Cross section of lung tissue treated with 15% ethanol in primary fixative: Fly-in from overview to three different regions high-lighting details of surface/mesothelium, subcellular organelles, and type 2 alveolar cells

File name: Supplementary Movie 3

Description: High resolution FIBSEM nanotomography data of lung surface with attached pectin patch: Fly-through of 3D stack

File name: Supplementary Movie 4a-c

Description: Annotated data stack, 3D visualization from different angles

File name: Supplementary Data 1

Description: source data to Figure 4A - results

File name: Supplementary Data 2

Description: source data to Figure 4A - calibration
