# Supplementary material for: Plant heteropolysaccharides and their bioadhesion to animal glycocalyx visualized at the nanoscale
Source: Commun Biol. 2026 Sep 22;9:1238. doi: 10.1038/s42003-026-10872-y (PMC13597490; doi:10.1038/s42003-026-10872-y)
Supplement: Supplementary file 5 [file 42003_2026_10872_MOESM9_ESM.pdf]

02.03.26

Methanolysis and TFA hydrolysis
GalA, galacturonic acid;
GlcA, glucuronic acid;

in duplicate

L1 Lemon pectin	L1 A	L1 B
L2 Lemon pectin	L2 A	L2 B
Orange pectin	OA	OB
Soy pectin	Soy A	Soy B
Potato pectin	PA	PB
Sugarbeet pectin	SuA	SuB

concentration in pmol from the calibration
red value is not within the calibration range, other dilutions necessary
tr., trace amounts, n.d., not detected,

	peak area	Fucose	Rhamnose	Arabinose	Galactose	Glucose	Xylose	Mannose	GalA	GlcA
diluted 1 to 20	L1 A Lemon pectin	0,223	1,931	3,345	14,362	1,758	0,354	0,212	20,753	0,197
diluted 1 to 20	L1 B Lemon pectin	0,413	3,272	5,624	22,886	3,222	0,662	0,437	39,585	0,333
diluted 1 to 20	L2 A Lemon pectin	0,320	2,586	4,527	18,299	2,436	0,508	0,311	32,825	0,232
diluted 1 to 20	L2 B Lemon pectin	0,450	3,320	5,983	23,164	2,334	0,684	0,369	33,685	0,417
diluted 1 to 20	OA Orange pectin	0,417	2,748	7,727	15,008	4,826	0,796	0,268	29,494	0,284
diluted 1 to 20	OB Orange pectin	0,448	2,757	7,652	15,092	4,844	0,824	0,289	30,500	0,317
diluted 1 to 20	Soy A Soy pectin	15,141	10,471	7,458	23,476	1,007	30,759		47,015	1,076
diluted 1 to 20	Soy B Soy pectin	14,900	10,361	7,313	23,126	0,989	30,236		45,510	1,093
diluted 1 to 20	PA Potato pectin		9,131	26,044	73,663	1,729	1,949	0,351	18,926	0,546
diluted 1 to 20	PB Potato pectin		7,231	21,675	61,377	1,330	1,403	0,173	13,421	0,394
diluted 1 to 20	SuA Sugarbeet pectin	0,112	4,407	43,948	22,757	28,288	5,103	1,850	4,955	3,013
diluted 1 to 20	SuB Sugarbeet pectin	0,073	4,099	42,403	21,847	23,206	4,837	1,656	4,429	2,596
other dilutions										
diluted 1 to 50	Soy A Soy pectin	7,610	5,103	3,074	12,643	0,402	15,739		20,370	0,513
diluted 1 to 50	Soy B Soy pectin	7,271	4,945	2,949	12,220	0,390	15,210		19,474	0,487
diluted 1 to 100	PA Potato pectin		2,341	7,877	32,975	0,378	0,348		3,896	0,107
diluted 1 to 100	PB Potato pectin		1,731	5,895	27,767	0,559	0,244		2,799	0,080
diluted 1 to 50	SuA Sugarbeet pectin		2,034	27,573	11,839	15,456	2,077	0,715	2,085	1,272
diluted 1 to 50	SuB Sugarbeet pectin		1,865	26,723	11,122	11,708	1,952	0,613	1,878	1,047
diluted 1 to 10	L1 A Lemon pectin	0,476	3,910	7,120	25,121	4,039	0,847	0,521	41,351	0,258
diluted 1 to 10	L2 A Lemon pectin	0,555	4,625	8,279	28,325	5,128	1,021	0,649	59,950	0,412
diluted 1 to 10	OA Orange pectin	0,930	5,294	14,753	26,060	10,403	1,834	0,689	62,048	0,538
diluted 1 to 10	OB Orange pectin	0,984	5,528	15,109	26,958	10,792	1,939	0,731	67,498	0,680

	concentation in pmol	Fucose	Rhamnose	Arabinose	Galactose	Glucose	Xylose	Mannose	GalA	GlcA	SUM	
diluted 1 to 20	L1 A Lemon pectin	tr	210,48	226,85	762,06	53,92	19,77	17,49	1563,65	tr	2854,22	Xyl, Man: from dillution 1 to 10 /2
diluted 1 to 20	L1 B Lemon pectin	tr	373,71	398,46	1480,21	107,66	30,40	29,47	3012,57	tr	5432,48	
diluted 1 to 20	L2 A Lemon pectin	tr	288,23	313,89	1072,37	78,55	24,07	21,68	2483,70	tr	4282,48	Xyl, Man: from dillution 1 to 10 /2
diluted 1 to 20	L2 B Lemon pectin	tr	379,90	427,01	1506,99	74,82	31,48	25,05	2550,48	tr	4995,74	
diluted 1 to 20	OA Orange pectin	tr	307,92	572,95	810,99	169,02	37,00	23,00	2226,79	tr	4147,66	Man: from dillution 1 to 10 /2
diluted 1 to 20	OB Orange pectin	tr	309,08	566,43	817,34	169,71	38,39	24,37	2304,20	tr	4229,52	Man: from dillution 1 to 10 /2
diluted 1 to 20	Soy A Soy pectin	1147,13	1479,26	549,57	1537,41	27,12	2915,10	n.d.	3837,22	59,79	11552,60	GalA: from dilution 1 to 50 x 2,5
diluted 1 to 20	Soy B Soy pectin	1120,95	1459,60	537,09	1503,32	26,46	2789,90	n.d.	3669,68	60,76	11167,77	GalA: from dilution 1 to 50 x 2,5
diluted 1 to 20	PA Potato pectin	n.d.	1247,09	2764,60	13833,98	52,88	94,87	tr	1426,95	29,55	19449,93	Gal: from dilution 1 to 100 x 5
diluted 1 to 20	PB Potato pectin	n.d.	940,95	2029,97	10002,14	38,60	67,21	tr	1018,99	tr	14097,85	Gal: from dilution 1 to 100 x 5
diluted 1 to 20	SuA Sugarbeet pectin	n.d.	526,91	7832,25	1467,83	1601,47	264,20	123,11	382,10	171,60	12369,46	Ara: from dilution 1 to 50 x2,5
diluted 1 to 20	SuB Sugarbeet pectin	n.d.	483,75	7287,92	1382,37	1195,43	249,21	110,13	341,45	147,36	11197,62	Ara: from dilution 1 to 50 x2,5
other dilutions												
diluted 1 to 50	Soy A Soy pectin								1534,89			
diluted 1 to 50	Soy B Soy pectin								1467,87			
diluted 1 to 100	PA Potato pectin				2766,80							
diluted 1 to 100	PB Potato pectin				2000,43							
diluted 1 to 50	SuA Sugarbeet pectin			3132,90								
diluted 1 to 50	SuB Sugarbeet pectin			2915,17								
diluted 1 to 10	L1 A Lemon pectin						39,55	34,98				
diluted 1 to 10	L2 A Lemon pectin						48,13	43,35				
diluted 1 to 10	OA Orange pectin							46,00				
diluted 1 to 10	OB Orange pectin							48,74				

mol%	Fucose	Rhamnose	Arabinose	Galactose	Glucose	Xylose	Mannose	GalA	GlcA
L1 A Lemon pectin	tr	7,37	7,95	26,70	1,89	0,69	0,61	54,78	tr
L1 B Lemon pectin	tr	6,88	7,33	27,25	1,98	0,56	0,54	55,45	tr
L2 A Lemon pectin	tr	6,73	7,33	25,04	1,83	0,56	0,51	58,00	tr
L2 B Lemon pectin	tr	7,60	8,55	30,17	1,50	0,63	0,50	51,05	tr
OA Orange pectin	tr	7,42	13,81	19,55	4,07	0,89	0,55	53,69	tr
OB Orange pectin	tr	7,31	13,39	19,32	4,01	0,91	0,58	54,48	tr
Soy A Soy pectin	9,93	12,80	4,76	13,31	0,23	25,23	n.d.	33,22	0,52
Soy B Soy pectin	10,04	13,07	4,81	13,46	0,24	24,98	n.d.	32,86	0,54
PA Potato pectin	n.d.	6,41	14,21	71,13	0,27	0,49	tr	7,34	0,15
PB Potato pectin	n.d.	6,67	14,40	70,95	0,27	0,48	tr	7,23	tr
SuA Sugarbeet pectin	n.d.	4,26	63,32	11,87	12,95	2,14	1,00	3,09	1,39
SuB Sugarbeet pectin	n.d.	4,32	65,08	12,35	10,68	2,23	0,98	3,05	1,32

	mean mo%		Fucose	Rhamnose	Arabinose	Galactose	Glucose	Xylose	Mannose	GalA	GlcA	
A	**L1 Lemon pectin**		tr	7,37	7,95	26,70	1,89	0,69	0,61	54,78	tr	
B	**L1 Lemon pectin**		tr	6,88	7,33	27,25	1,98	0,56	0,54	55,45	tr	
		**mean**		**7,1**	**7,6**	**27,0**	**1,94**	**0,63**	**0,58**	**55,1**		
		range/2		0,2	0,3	0,3	0,05	0,07	0,04	0,3		
A	**L2 Lemon pectin**		tr	6,73	7,33	25,04	1,83	0,56	0,51	58,00	tr	
B	**L2 Lemon pectin**		tr	7,60	8,55	30,17	1,50	0,63	0,50	51,05	tr	
		**mean**		**7,2**	**7,9**	**27,6**	**1,7**	**0,60**	**0,50**	**54,5**		
		range/2		0,4	0,6	2,6	0,2	0,03	0,00	3,47		
A	**Orange pectin**		tr	7,42	13,81	19,55	4,07	0,89	0,55	53,69	tr	
B	**Orange pectin**		tr	7,31	13,39	19,32	4,01	0,91	0,58	54,48	tr	
		**mean**		**7,4**	**13,6**	**19,4**	**4,04**	**0,90**	**0,57**	**54,1**		
		range/2		0,1	0,2	0,1	0,03	0,01	0,01	0,4		
A	**Soy pectin**		9,93	12,80	4,76	13,31	0,23	25,23	n.d.	33,22	0,52	
B	**Soy pectin**		10,04	13,07	4,81	13,46	0,24	24,98	n.d.	32,86	0,54	
		**mean**	**10,0**	**12,9**	**4,78**	**13,4**	**0,236**	**25,1**		**33,0**	**0,53**	
		range/2	0,1	0,1	0,03	0,1	0,001	0,13		0,2	0,01	
A	**Potato pectin**		n.d.	6,41	14,21	71,13	0,27	0,49	tr	7,34	0,15	GlcA only one value
B	**Potato pectin**		n.d.	6,67	14,40	70,95	0,27	0,48	tr	7,23		
		**mean**		**6,5**	**14,3**	**71,0**	**0,273**	**0,48**		**7,3**	**0,15**	
		range/2		0,1	0,1	0,1	0,001	0,01		0,1		
A	**Sugar beet pectin**		n.d.	4,26	63,32	11,87	12,95	2,14	1,00	3,09	1,39	
B	**Sugar beet pectin**		n.d.	4,32	65,08	12,35	10,68	2,23	0,98	3,05	1,32	
		**mean**		**4,29**	**64,2**	**12,1**	**11,8**	**2,18**	**0,99**	**3,07**	**1,35**	
		range/2		0,03	0,9	0,2	1,1	0,04	0,01	0,02	0,04	
